# Supplementary material for: Revealing evolution of tropane alkaloid biosynthesis by analyzing two genomes in the Solanaceae family
Source: Nat Commun. 2023 Mar 15;14:1446. doi: 10.1038/s41467-023-37133-4 (PMC10017790; doi:10.1038/s41467-023-37133-4)
Supplement: Supplementary file 1 — Supplementary Information [file 41467_2023_37133_MOESM1_ESM.pdf]

# Revealing evolution of tropane alkaloid biosynthesis by analyzing two genomes in the Solanaceae family

Zhang *et al.*

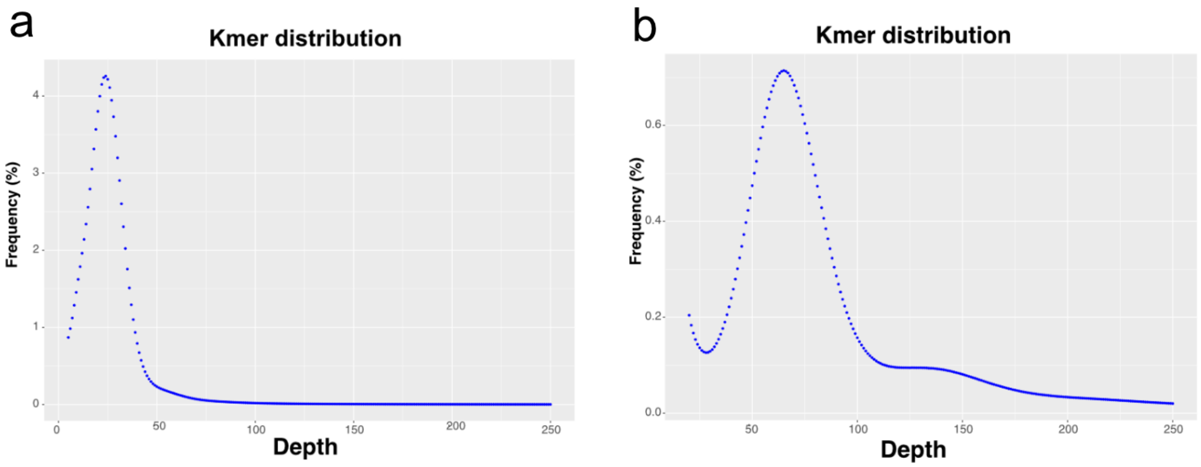


**Supplementary Fig. 1. 17 *K*-mer frequency distribution of *Atropa belladonna* and *Datura stramonium* using Illumina short reads. a** The *K*-mer distribution of *A. belladonna*. **b** The *K*-mer distribution of *D. stramonium*. The *K*-mers were counted using Jellyfish with the parameter "-C -m 51 -s 10000000000 -t 50" and imported into R to plot the frequency by ggplot2 package (https://ggplot2.tidyverse.org/). We identified 43,266,675,948 *K*-mers and the peak of *K*-mer depth is 26 in *A. belladonna*, and 115,136,234,620 *K*-mers and the peak of *K*-mer depth is 64 in *D. stramonium*. Genome size can be estimated as (total *K*-mer number) / (the volume peak). The genome sizes of *A. belladonna* and *D. stramonium* was thus estimated as ~1.65 Gb and ~1.80 Gb, respectively.

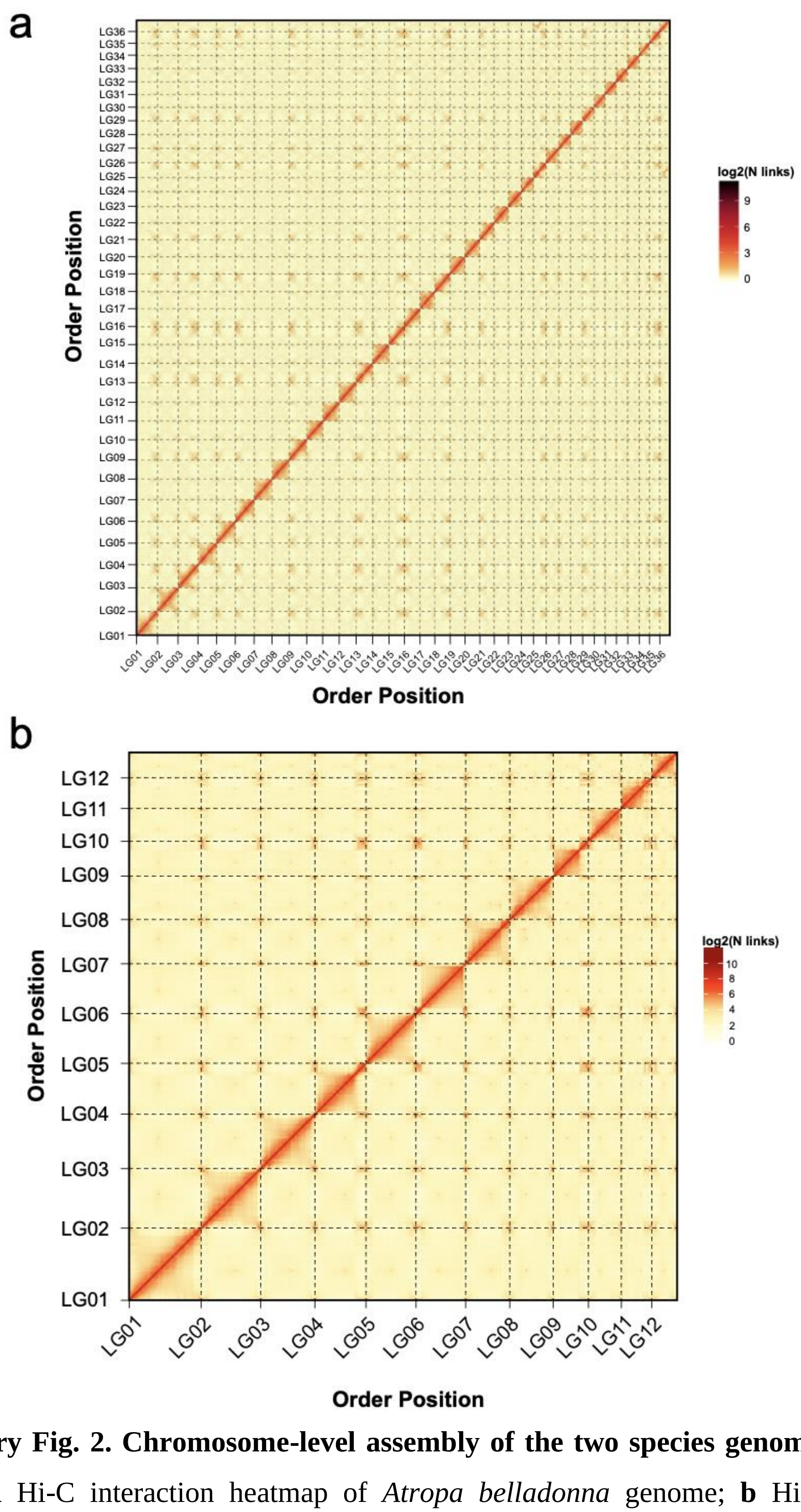


**Supplementary Fig. 2. Chromosome-level assembly of the two species genome using Hi-C technology**. **a** Hi-C interaction heatmap of *Atropa belladonna* genome; **b** Hi-C interaction heatmap of *Datura stramonium* genome.

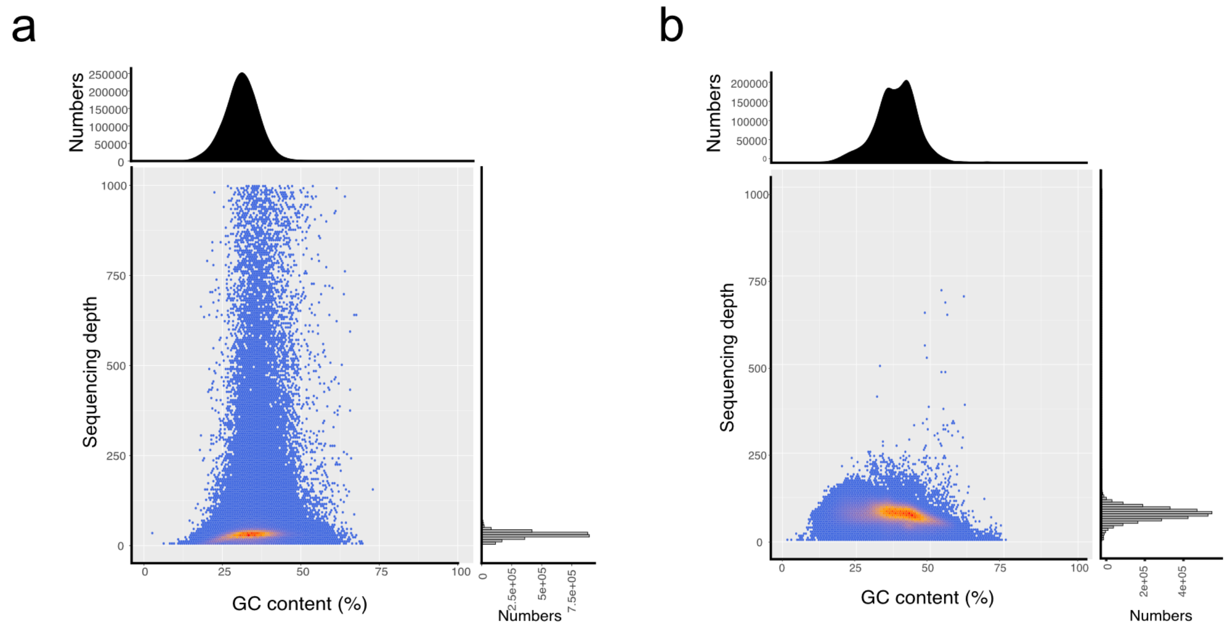


**Supplementary Fig. 3.** ***The genome-wide sequencing depth and GC depth distribution of Atropa belladonna* and *Datura stramonium*.** For the genome-wide analysis, the 500 bp non-overlapping sliding windows were generated to calculate GC content and sequencing depth of *A. belladonna* (**a**) and *D. stramonium* (**b**). The sequencing depth was calculated by BWA using short reads and assembly genomes of *A. belladonna* and *D. stramonium*. For each plot, the center is binhex plot of GC content and sequencing depth (bins = 150); the top is GC content density plot which shared the x axis of binhex plot; the right is the histogram of sequencing depth which used the y axis of binhex as x axis. The ggplot2 package (https://ggplot2.tidyverse.org/) in R was employed to plot.

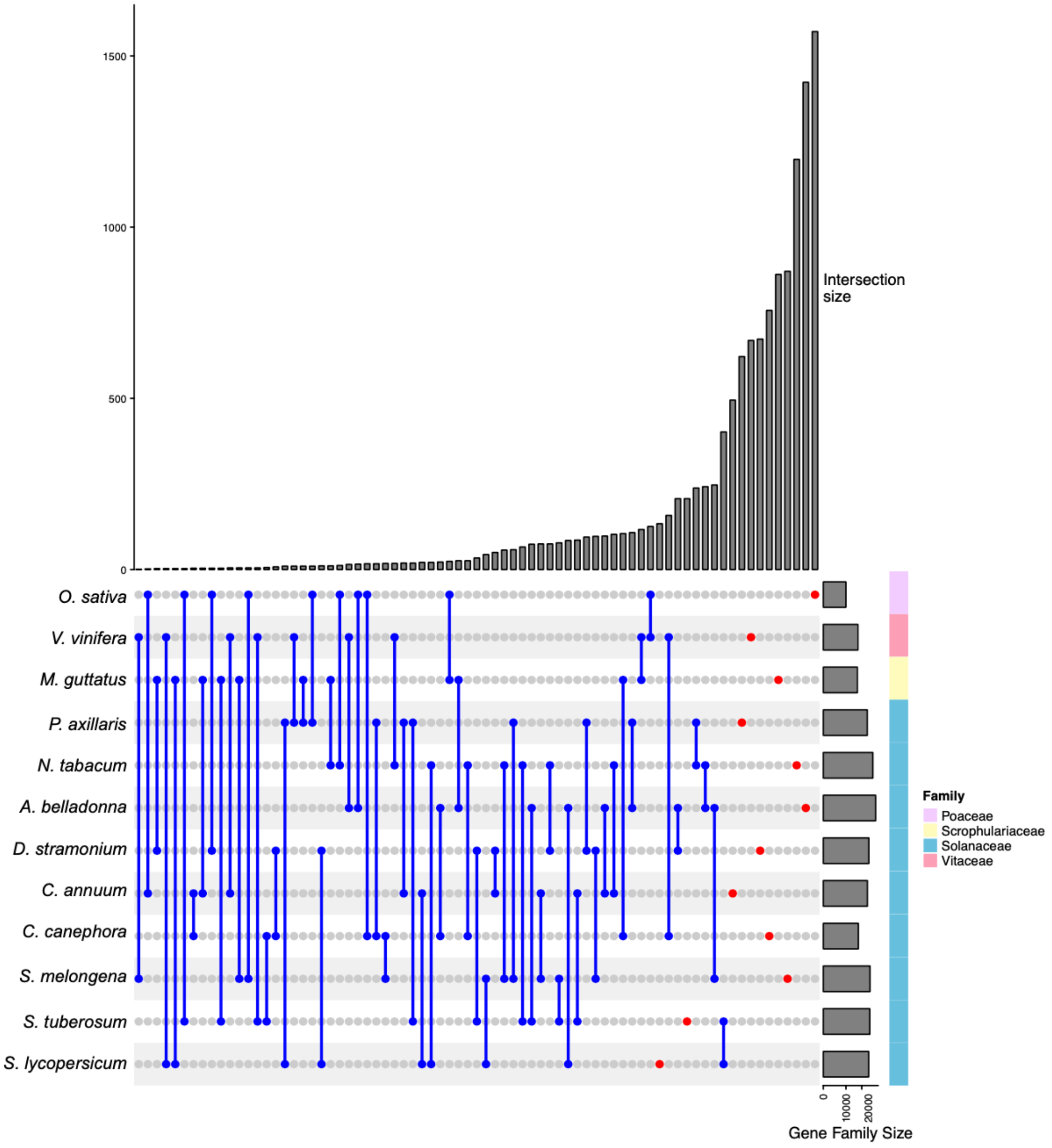


**Supplementary Fig. 4. Sharing of gene families by *A. belladonna* and other genomes.** The UpSet plot shows the intersections of the set of orthogroups from the twelve genomes. Each column corresponds to an orthogroup, and each row corresponds to species. Cells are either empty (grey black), indicating that this set is not part of that intersection, or filled, showing that the set is participating in the intersection and that the species share that orthogroup. The red cell represented the unique orthogroup of the species. The raw data for this plot is generated by OrthoMCL (v 2.0.9) and plotted by ComplexHeatmap package[1] in R with parameters (comb_degree = c(1, 2), mode = intersection). The raw data was provided as Supplementary Table 19.

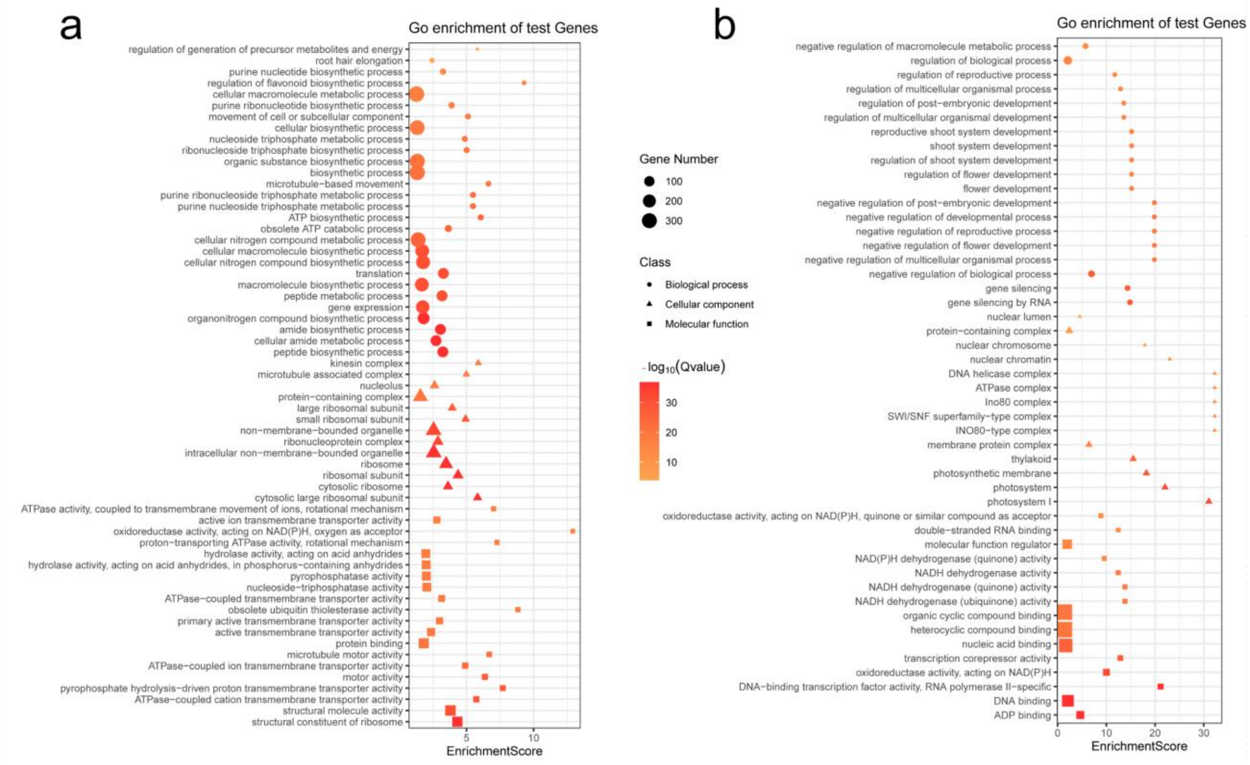


**Supplementary Fig. 5. The Gene Ontology (GO) enrichment analysis of expansion genes in *A. belladonna* and *D. stramonium*. a** The GO enrichment analysis of expansion genes in *A. belladonna*. **b** the GO enrichment analysis of expansion genes in *D. stramonium*. The GO enrichment analysis was performed using an online platform, OmicShare (https://www.omicshare.com/). GO terms with a *P*-value < 0.01 and a Q-value < 0.05 were defined as the significant terms. The *P*-values are estimated by two-sided hypergeometric test, and the Q-value are estimated by Benjaminiand Hochberg (BH) method. The raw data was provided in Supplementary Data 1 and 2.

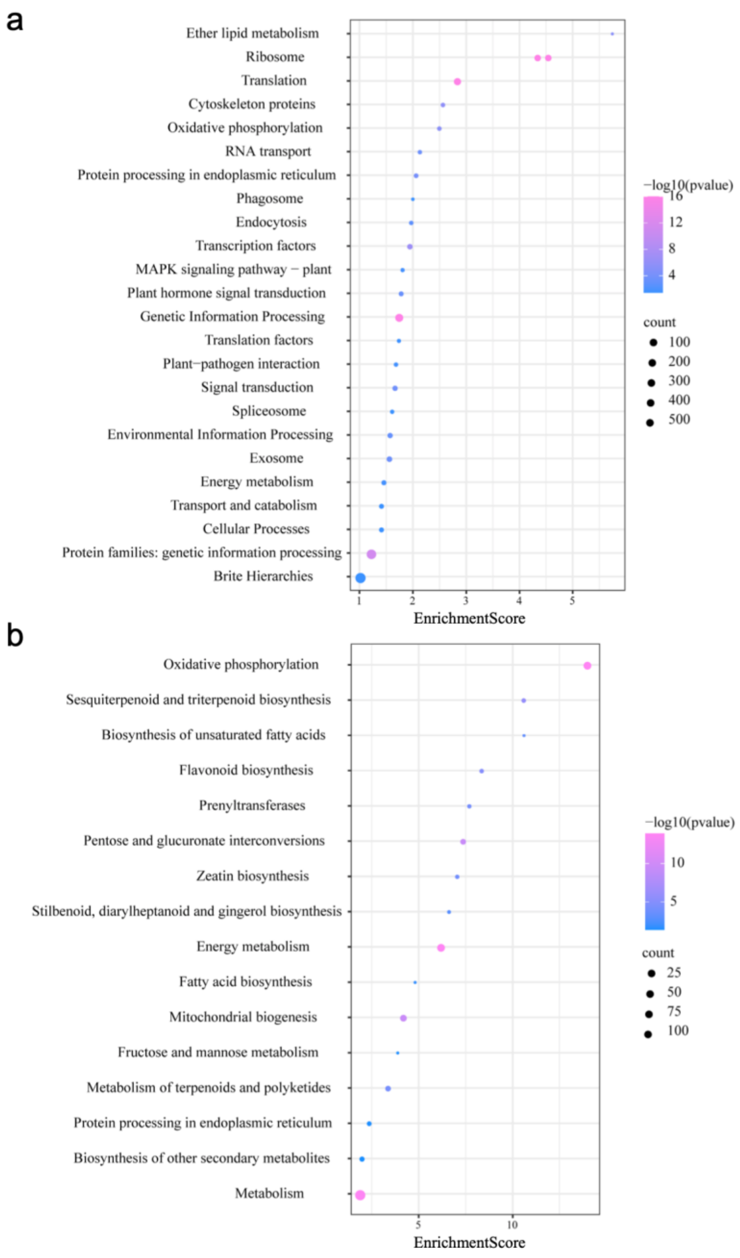


**Supplementary Fig. 6. The Kyoto Encyclopedia of Genes and Genomes (KEGG) enrichment analysis of expansion genes in *A. belladonna* and *D. stramonium*. a** The KEGG enrichment analysis of expansion genes in *A. belladonna*. **b** the KEGG enrichment analysis of expansion genes in *D. stramonium*. KEGG terms with a *P*-value < 0.01 and an Adjusted *P*-value < 0.05 were defined as the significant terms. The *P*-values are estimated by two-sided hypergeometric test, and the adjusted *P*-value are estimated by Benjaminiand Hochberg (BH) method. The KEGG enrichment analysis were performed using an online platform, OmicShare (https://www.omicshare.com/). The raw data was provided in Supplementary Tables 20 and 21.

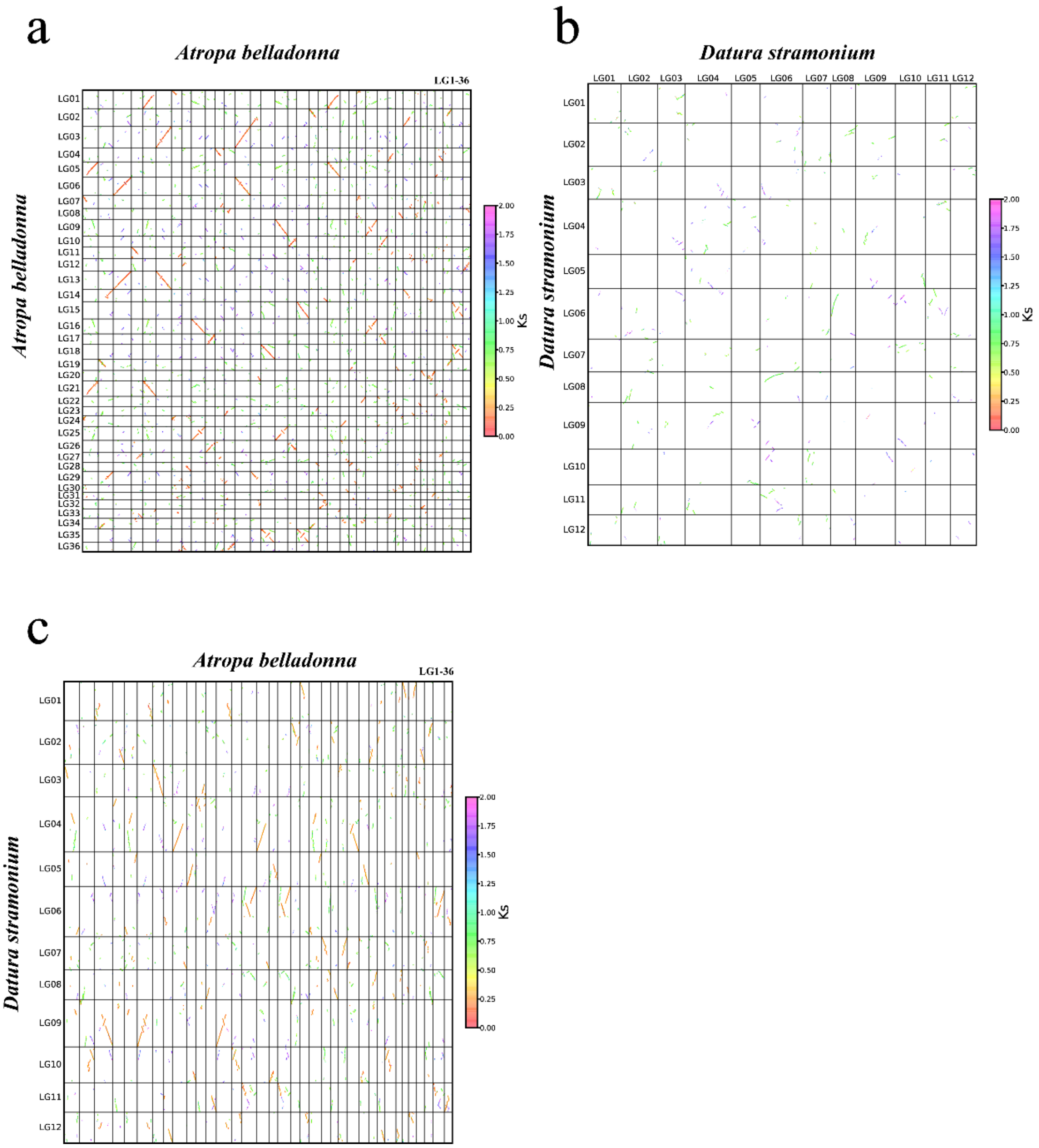


**Supplementary Fig. 7. Syntenic block dot plot between the *A. belladonna* and *D. stramonium* genomes.** **a** Dot plot of syntenic blocks of *A. belladonna* against itself; **b** Dot plot of syntenic blocks of *D. stramonium* against itself; **c** Dot plot of syntenic blocks between *A. belladonna* and *D. stramonium*.

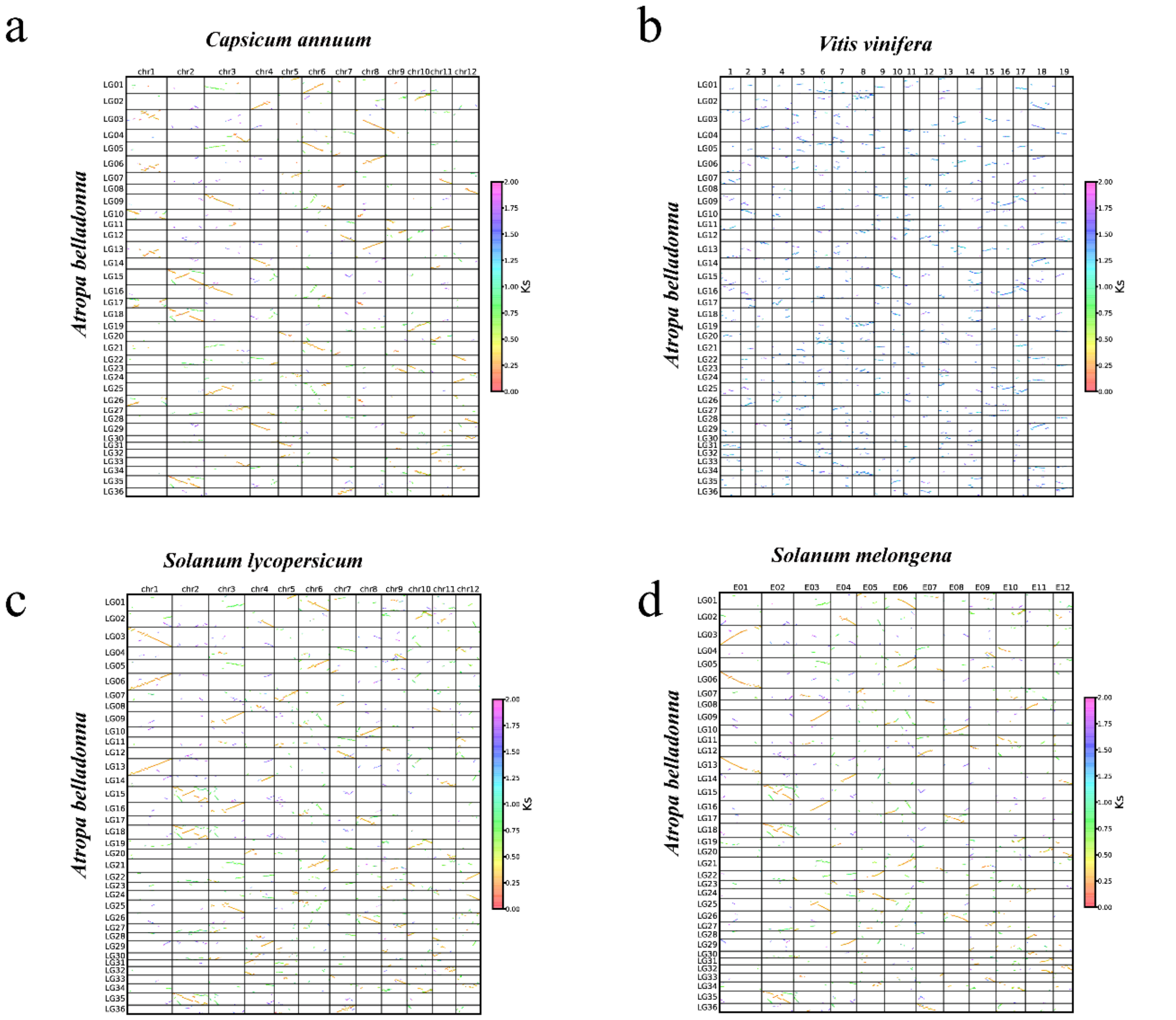


**Supplementary Fig. 8. Syntenic block dot plot between *A. belladonna* and other species.**

**a** Dot plot of syntenic blocks between *A. belladonna* and *C. annuum*; **b** Dot plot of syntenic blocks between *A. belladonna* and *V. vinifera;* **c** Dot plot of syntenic blocks between *A. belladonna* and *S. lycopersicum*; **d** Dot plot of syntenic blocks between *A. belladonna* and *S. melongena*.

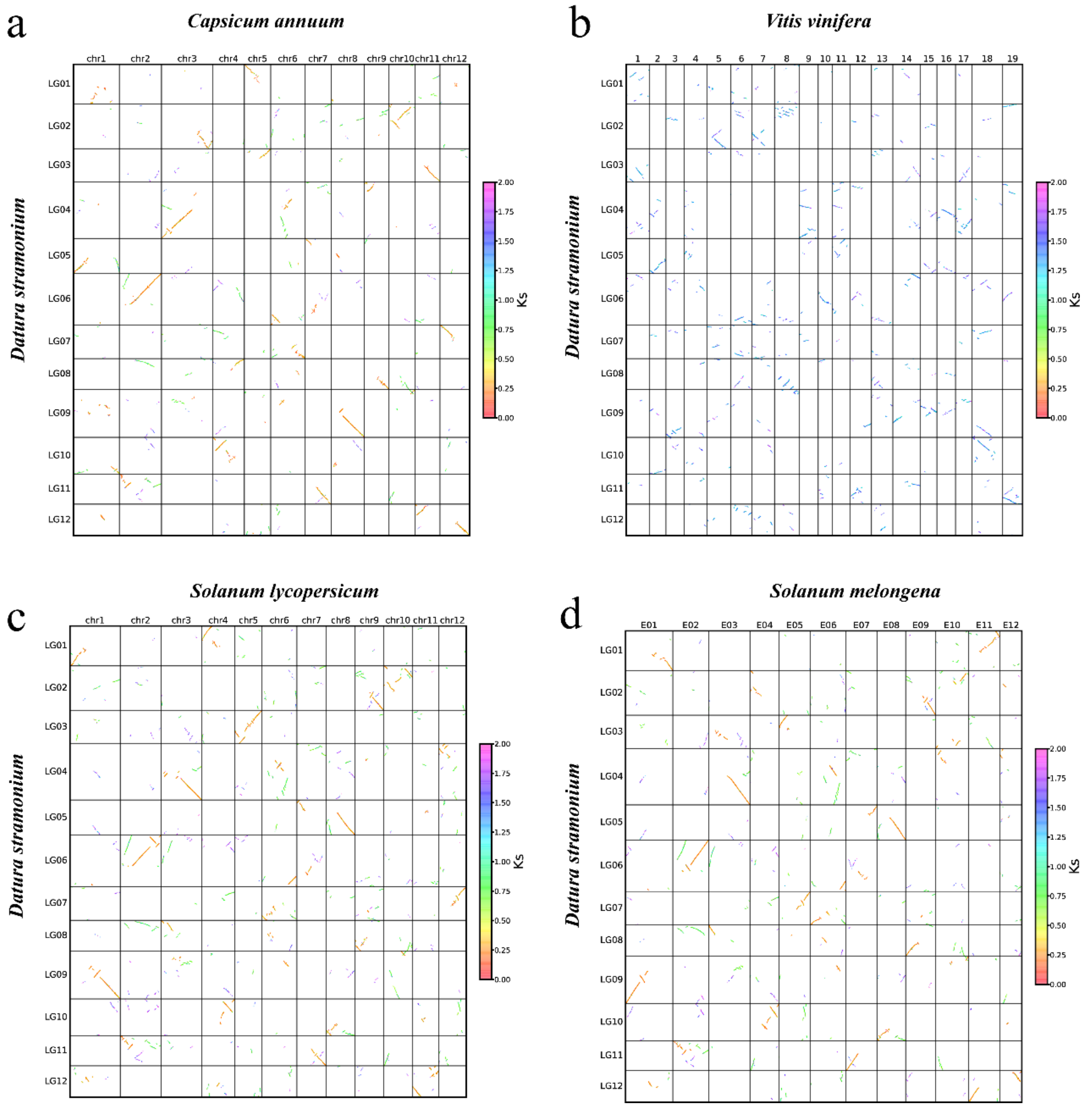


**Supplementary Fig. 9. Syntenic block dot plot between *D. stramonium* and other species.**

**a** Dot plot of syntenic blocks between *D. stramonium* and *C. annuum*; **b** Dot plot of syntenic blocks between *D. stramonium* and *V. vinifera*; **c** Dot plot of syntenic blocks between *D. stramonium* and *S. lycopersicum*; **d** Dot plot of syntenic blocks between *D. stramonium* and *S. melongena*.

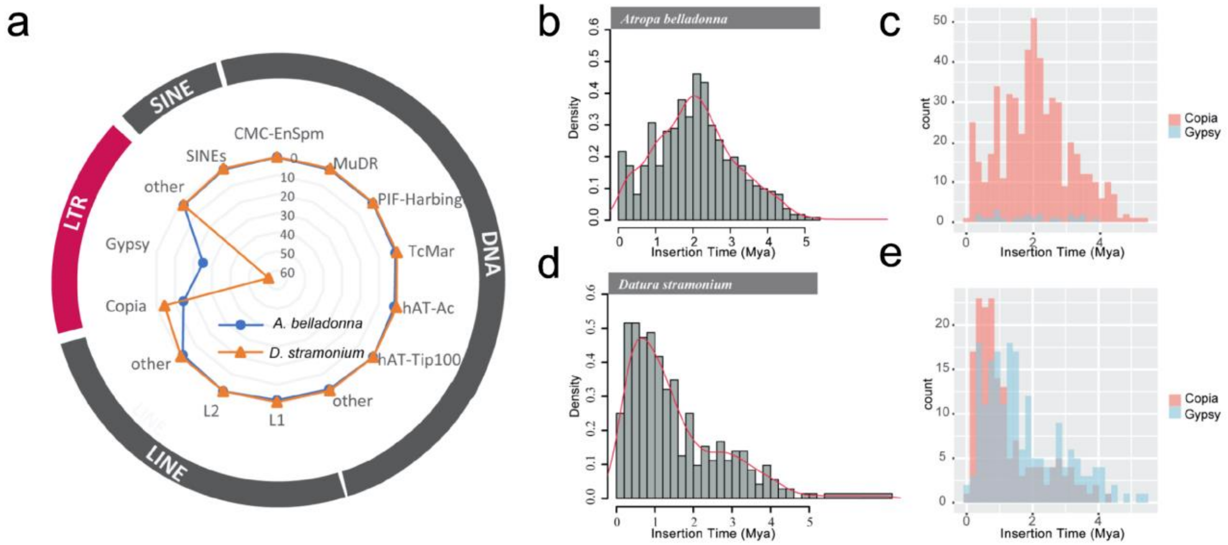


**Supplementary Fig. 10. Genome size variation and evolution of retrotransposon families in the *A. belladonna* and *D. stramonium* genomes. a** The great differences in the composition of repetitive sequences in *A. belladonna* and *D. stramonium*. The coordinates represent the ratio of each category to the total length of the corresponding genome assembly. **b** Insertion times of long terminal repeat (LTR) retrotransposons in *A. belladonna*. **c** Insertion times of Copia and Gypsy retrotransposons in *A. belladonna*. **d** Insertion times of LTR retrotransposons in *D. stramonium*. **e** Insertion times of Copia and Gypsy retrotransposons in *D. stramonium*. Mya, million years ago.

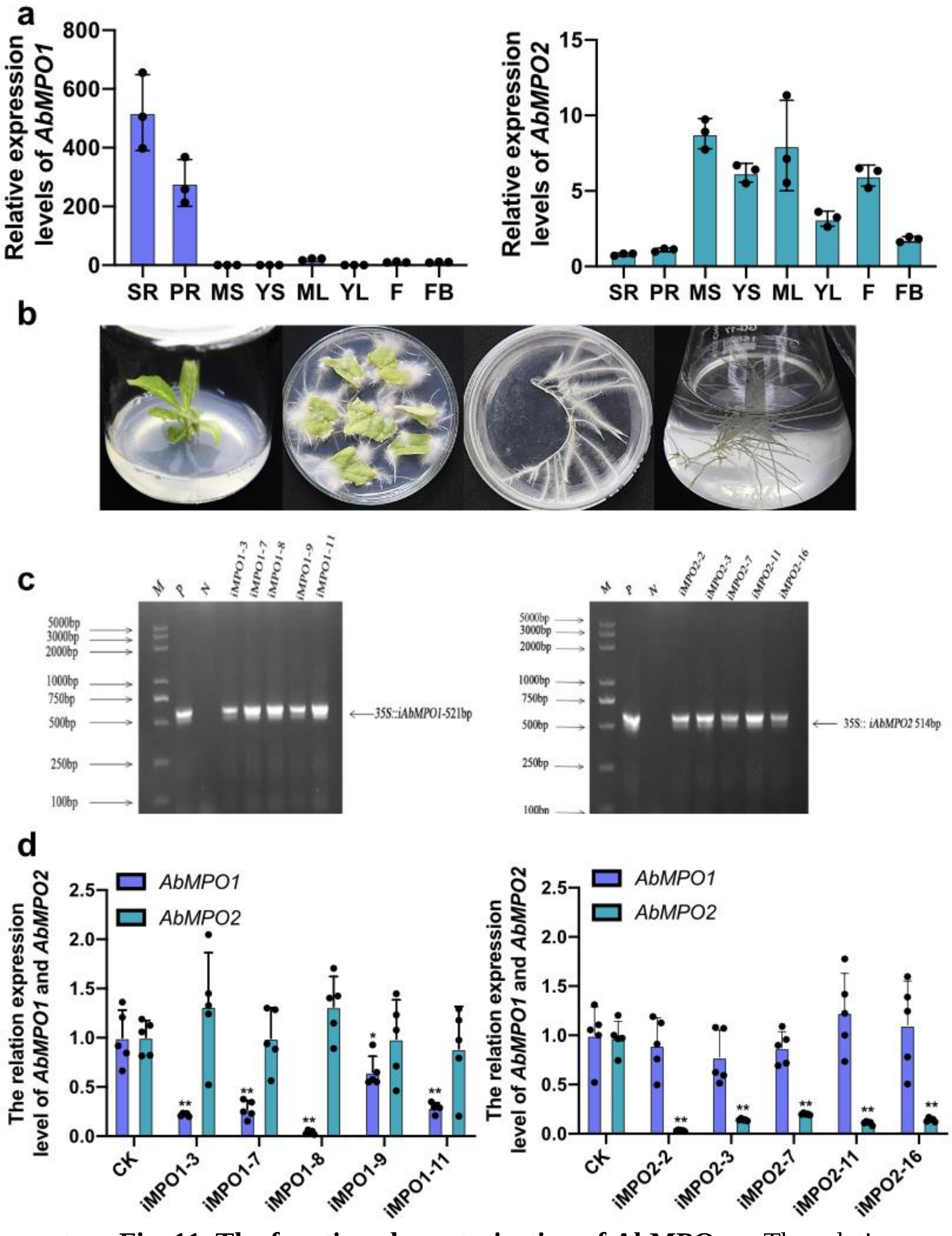


**Supplementary Fig. 11. The function characterization of AbMPOs**. **a** The relative expression levels of *AbMPO1* (left) and *AbMPO2* (right) in tissues of *A. belladonna*. SR: secondary root; PR: primary root; MS: mature stem; YS: young stem; ML: mature leaf; YL: young leaf; F: flower; FB: flower bud. The data are shown as means ± s.d. (n = 3 biologically independent samples). **b** Establishment of hairy root of *A. belladonna*. From the left to right, the figures represented seedlings, transformed roots induced from wounded leaves, a monoclony hairy root line, and root

cultures in liquid medium. **c** Detecting the target genes in transformed hairy root of *A. belladonna* by genomic DNA PCR. Left: the PCR results by using genomic DNA exacted from *AbMPO1* RNAi transformed hairy roots; right: the PCR results by using genomic DNA exacted from *AbMPO2* RNAi transformed hairy roots. M: marker; P: positive control (the constructed plasmid); N, negative control (the empty plasmid). This experiment was repeated independently three times with similar results. The unit for DNA molecular weight marker is base pair (bp). **d** The relative expression levels of *AbMPO1* and *AbMPO2* in hairy root cultures of *AbMPO1* (iMPO1, on the left) or *AbMPO2* (iMPO2, on the right) suppressed *A. belladonna*. CK, control root cultures, in which neither *AbMPO1* nor *AbMPO2* was suppressed. The data are shown as means ± s.d. (n = 5 biologically independent samples). * represents significant difference from control line (CK) analyzed by two-sided Student's *t*-test at the level of $p < 0.05$; ** represents significant difference from control line (CK) analyzed by two-sided Student's *t*-test at the level of $p < 0.01$. For relative expression of genes in iMPO1, ***P* = 0.0002 (iMPO1-3), ***P* = 0.0005 (iMPO1-7), ***P* = 0.0001 (iMPO1-8), **P* = 0.0349 (iMPO1-9), ***P* = 0.0004 (iMPO1-11). For relative expression of genes in iMPO2, ***P* = 0.0000 (iMPO2-2), ***P* = 0.0000 (iMPO2-3), ***P* = 0.0000 (iMPO2-7), **P* = 0.0000 (iMPO2-11), ***P* = 0.0000 (iMPO2-16). Source data are provided as a Source Data file.

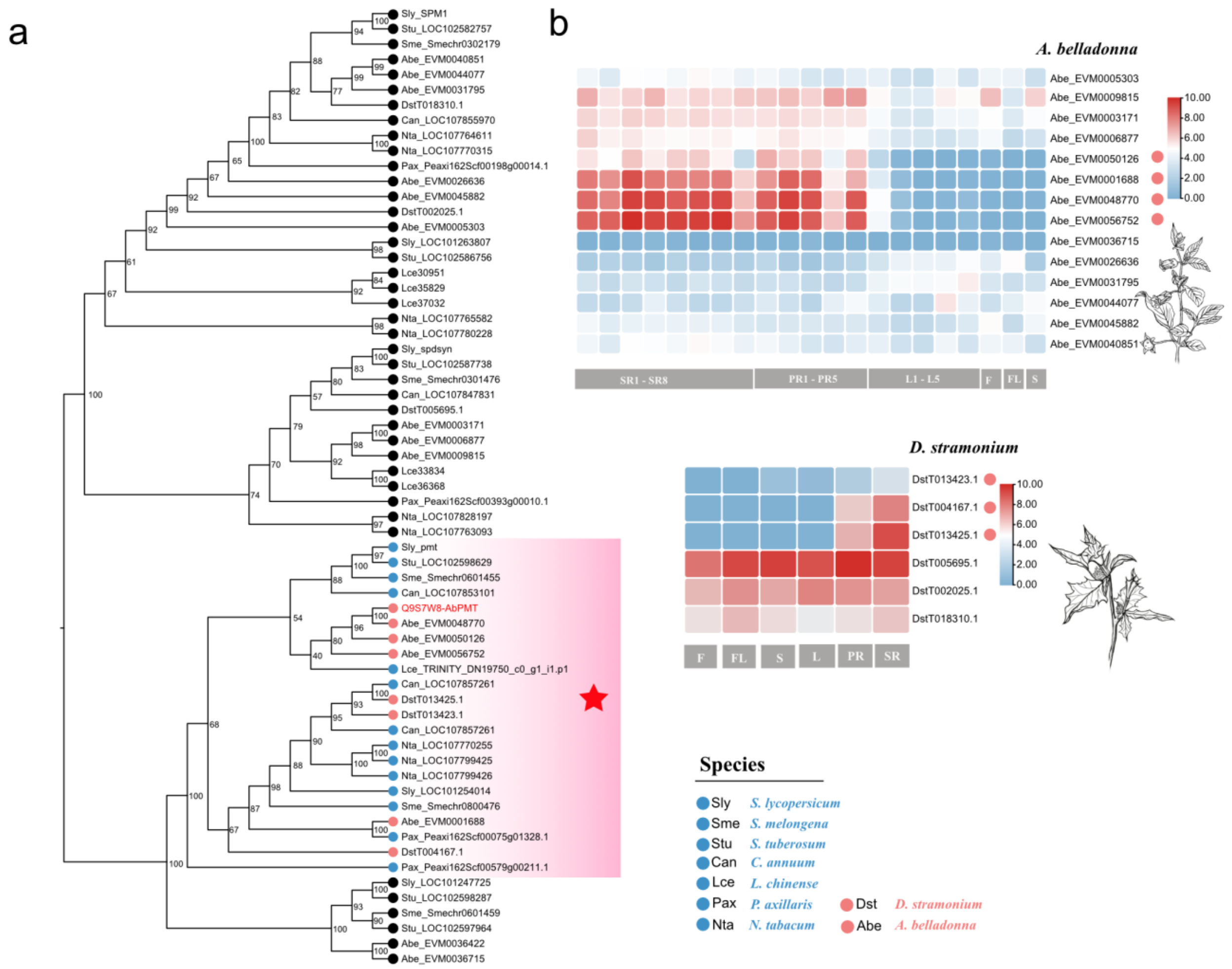


**Supplementary Fig. 12. Maximum-likelihood phylogeny of putrescine *N*-methyltransferase (PMT). a** Maximum likelihood phylogenetic tree of PMT family. Red dots highlight the genes from species with mTAs. Blue dots highlight the genes in species without mTAs. The red font indicates the functional characterized gene downloaded from NCBI. The support value was placed on the branch with bootstrap (n=1000). **b** Gene expression profiles (in normalized TPMs) of different tissues in two species are presented in the heatmap alongside the gene names (SR: secondary roots; PR: primary roots; L: leaf; F: fruit; FL: flower; S: stem). Source data are provided as a Source Data file.

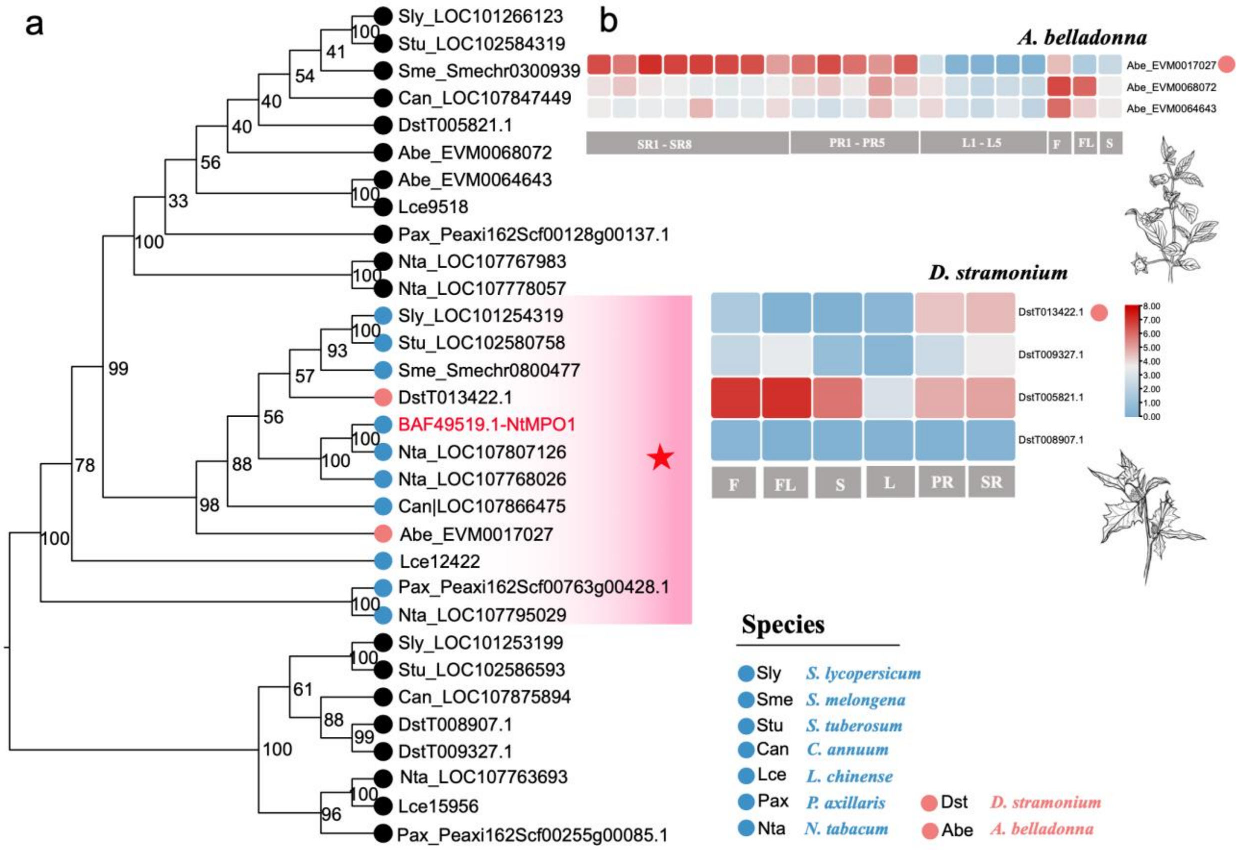


**Supplementary Fig. 13. Maximum-likelihood phylogeny of *N*-methylputrescine oxidase (MPO). a** Maximum likelihood phylogenetic tree of MPO family. Red dots highlight the genes from species with mTAs. Blue dots highlight the genes from species without mTAs. The red font indicates the functional characterized gene downloaded from NCBI. The support value was placed on the branch with bootstrap (n=1000). **b** Gene expression profiles (in normalized TPMs) of different tissues in two species are presented in the heatmap alongside the gene names (SR: secondary roots; PR: primary roots; L: leaf; F: fruit; FL: flower; S: stem). Source data are provided as a Source Data file.

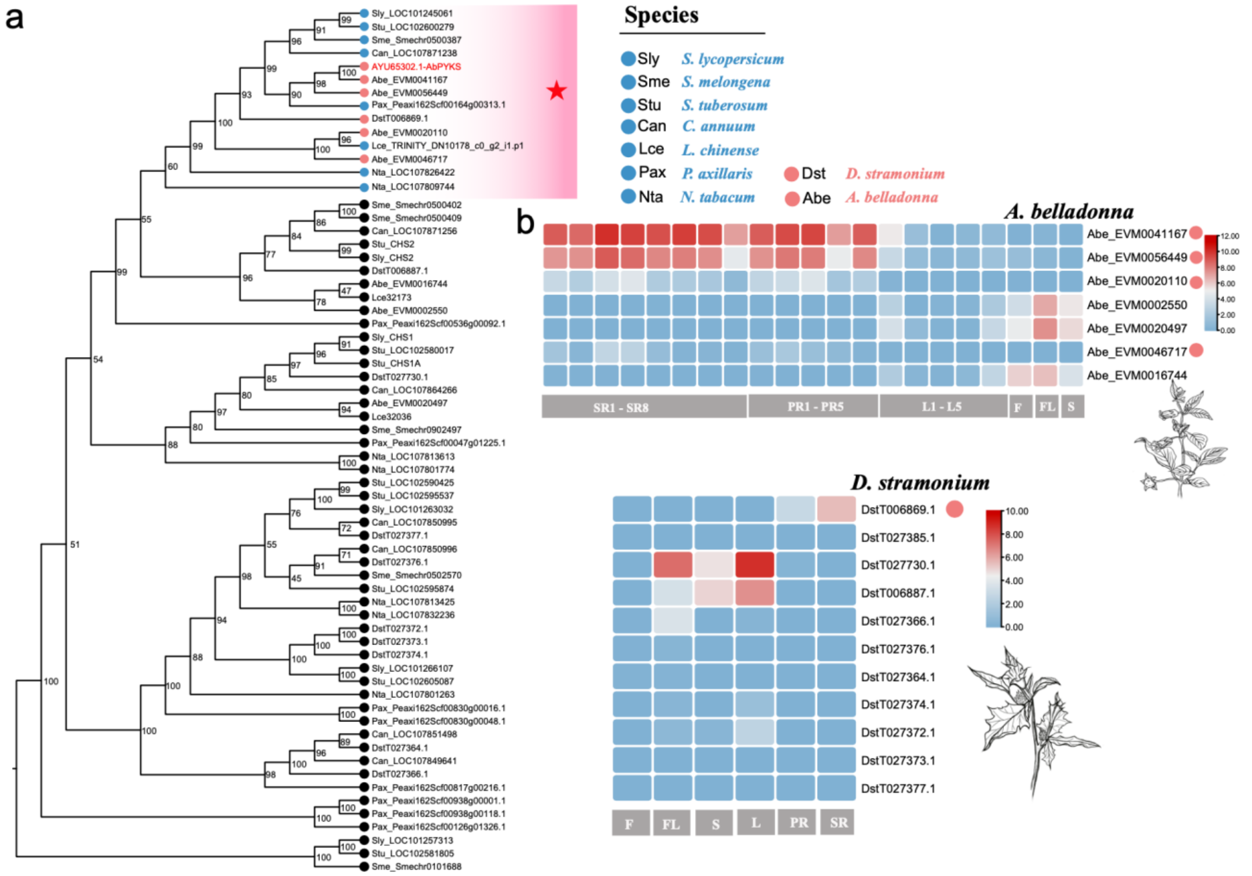


**Supplementary Fig. 14. Maximum-likelihood phylogeny of type III polyketide synthase (PYKS). a** Maximum likelihood phylogenetic tree of PYKS family. Red dots highlight the genes from species with mTAs. Blue dots highlight the genes from species without mTAs. The red font indicates the functional characterized gene downloaded from NCBI. The support value was placed on the branch with bootstrap (n=1000). **b** Gene expression profiles (in normalized TPMs) of different tissues in two species are presented in the heatmap alongside the gene names (SR: secondary roots; PR: primary roots; L: leaf; F: fruit; FL: flower; S: stem). Source data are provided as a Source Data file.

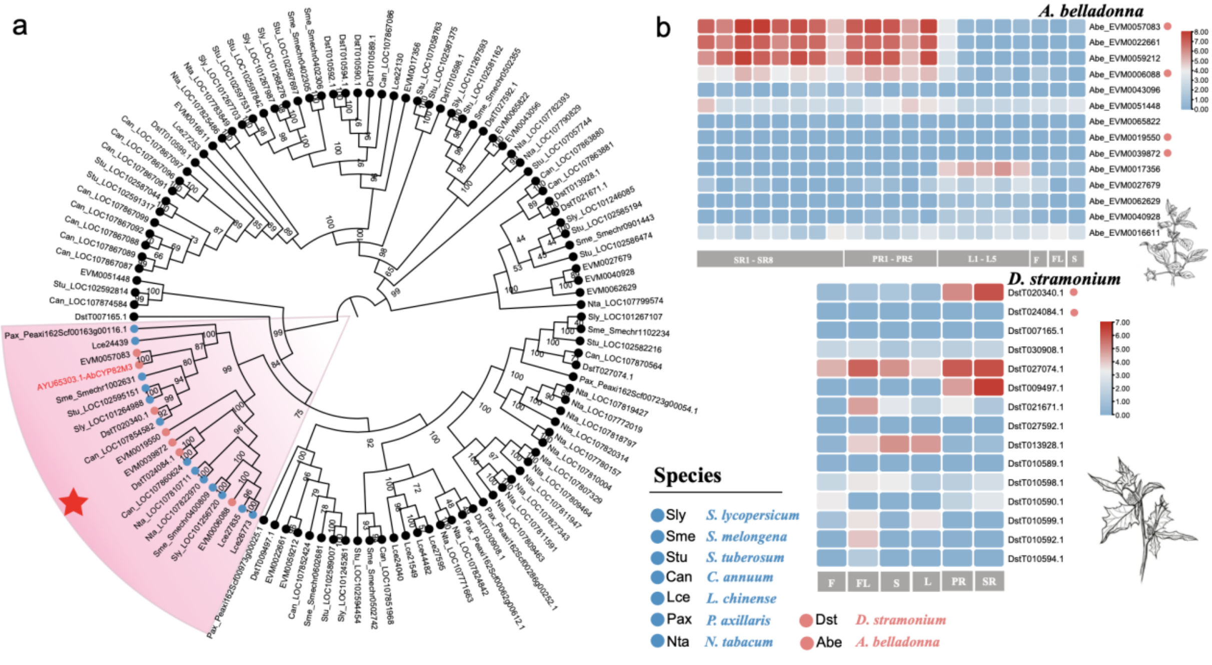


**Supplementary Fig. 15. Maximum-likelihood phylogeny of tropinone synthase (CYP82M3).** **a** Maximum likelihood phylogenetic tree of CYP82M3 family. Red dots highlight the genes from species with mTAs. Blue dots highlight the genes from species without mTAs. The Red font indicates the functional characterized gene downloaded from NCBI. The support value was placed on the branch with bootstrap (n=1000). **b** Gene expression profiles (in normalized TPMs) of different tissues in two species are presented in the heatmap alongside the gene names (SR: secondary roots; PR: primary roots; L: leaf; F: fruit; FL: flower; S: stem). Source data are provided as a Source Data file.

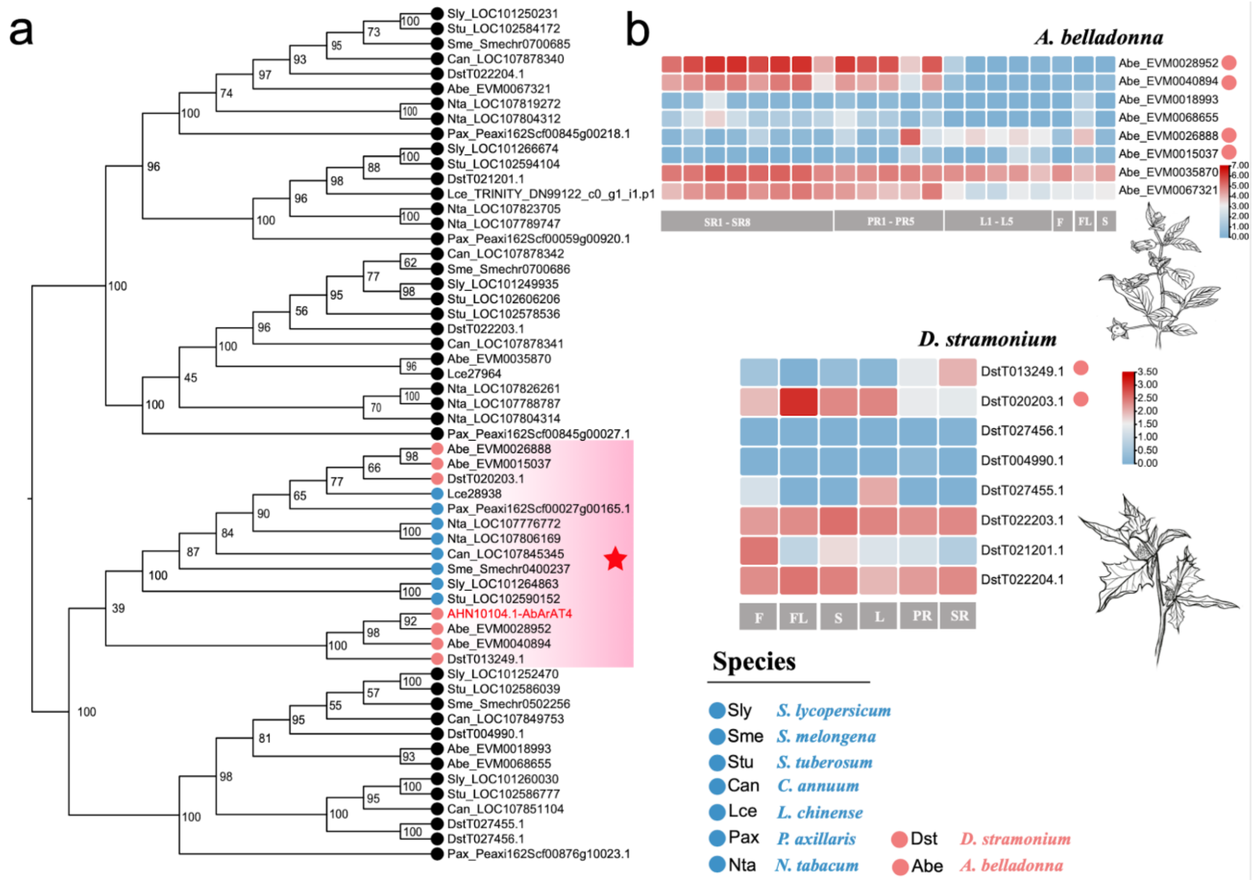


**Supplementary Fig. 16. Maximum-likelihood phylogeny of aromatic amino acid aminotransferase 4 (AT4). a** Maximum likelihood phylogenetic tree of AT4 family. Red dots highlight genes from species with mTAs. Blue dots highlight the genes from species without mTAs. The red font indicates the functional characterized gene downloaded from NCBI. The support value was placed on the branch with bootstrap (n=1000). **b** Gene expression profiles (in normalized TPMs) of different tissues in two species are presented in the heatmap alongside the gene names (SR: secondary roots; PR: primary roots; L: leaf; F: fruit; FL: flower; S: stem). Source data are provided as a Source Data file.

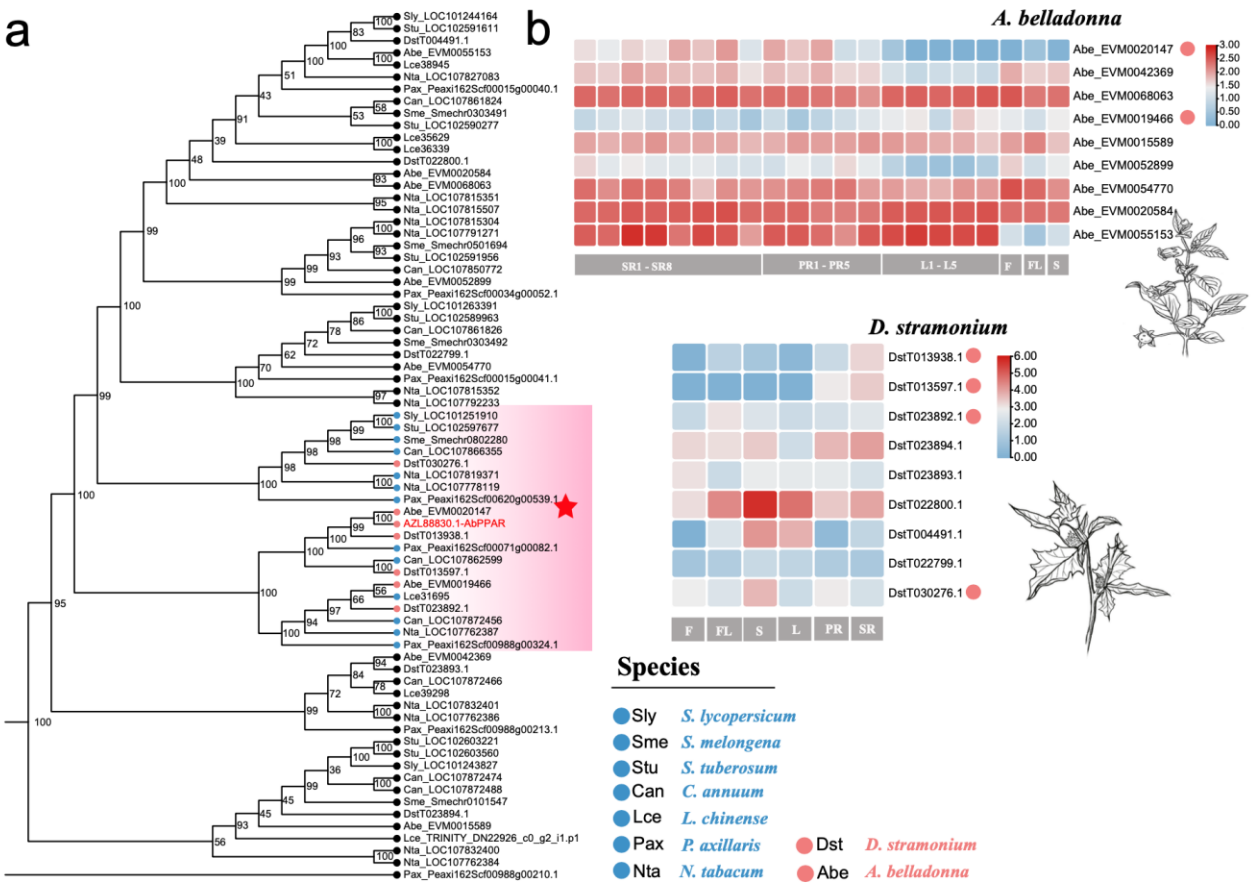


**Supplementary Fig. 17. Maximum-likelihood phylogeny of phenylpyruvic acid reductase (PPAR). a** Maximum likelihood phylogenetic tree of PPAR family. Red dots highlight the genes from species with mTAs. Blue dots highlight genes from species without mTAs. The red font indicates the functional verification gene downloaded from NCBI. The support value was placed on the branch with bootstrap (n=1000). **b** Gene expression profiles (in normalized TPMs) of different tissues in two species are presented in the heatmap alongside the gene names (SR: secondary roots; PR: primary roots; L: leaf; F: fruit; FL: flower; S: stem). Source data are provided as a Source Data file.

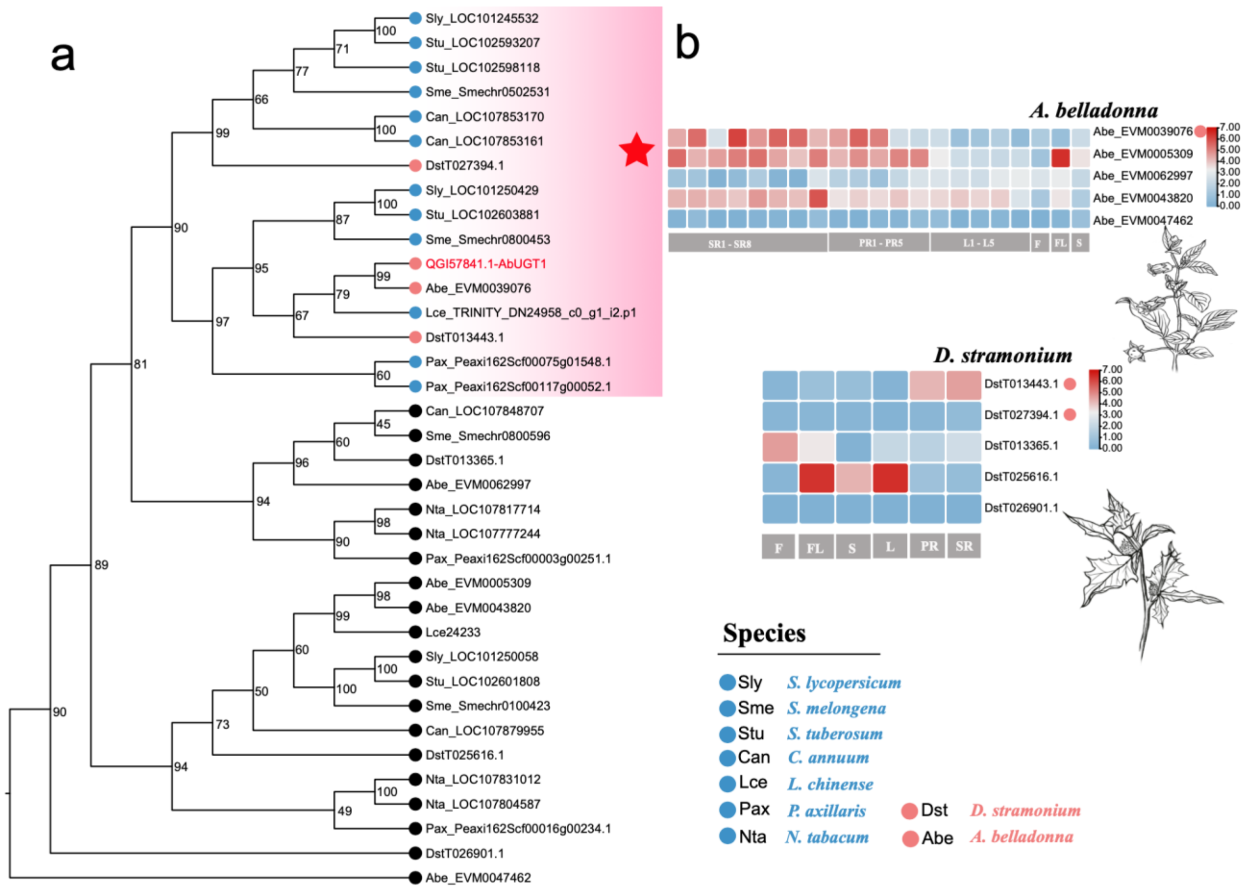


**Supplementary Fig. 18. Maximum-likelihood phylogeny of phenyllactate UDP-glycosyltransferase (UGT). a** Maximum likelihood phylogenetic tree of UGT1 family. Red dots highlight the genes from species with mTAs. Blue dots highlight the genes from species without mTAs. The red font indicates the functional characterized gene downloaded from NCBI. The support value was placed on the branch with bootstrap (n=1000). **b** Gene expression profiles (in normalized TPMs) of different tissues in two species are presented in the heatmap alongside the gene names (SR: secondary roots; PR: primary roots; L: leaf; F: fruit; FL: flower; S: stem). Source data are provided as a Source Data file.

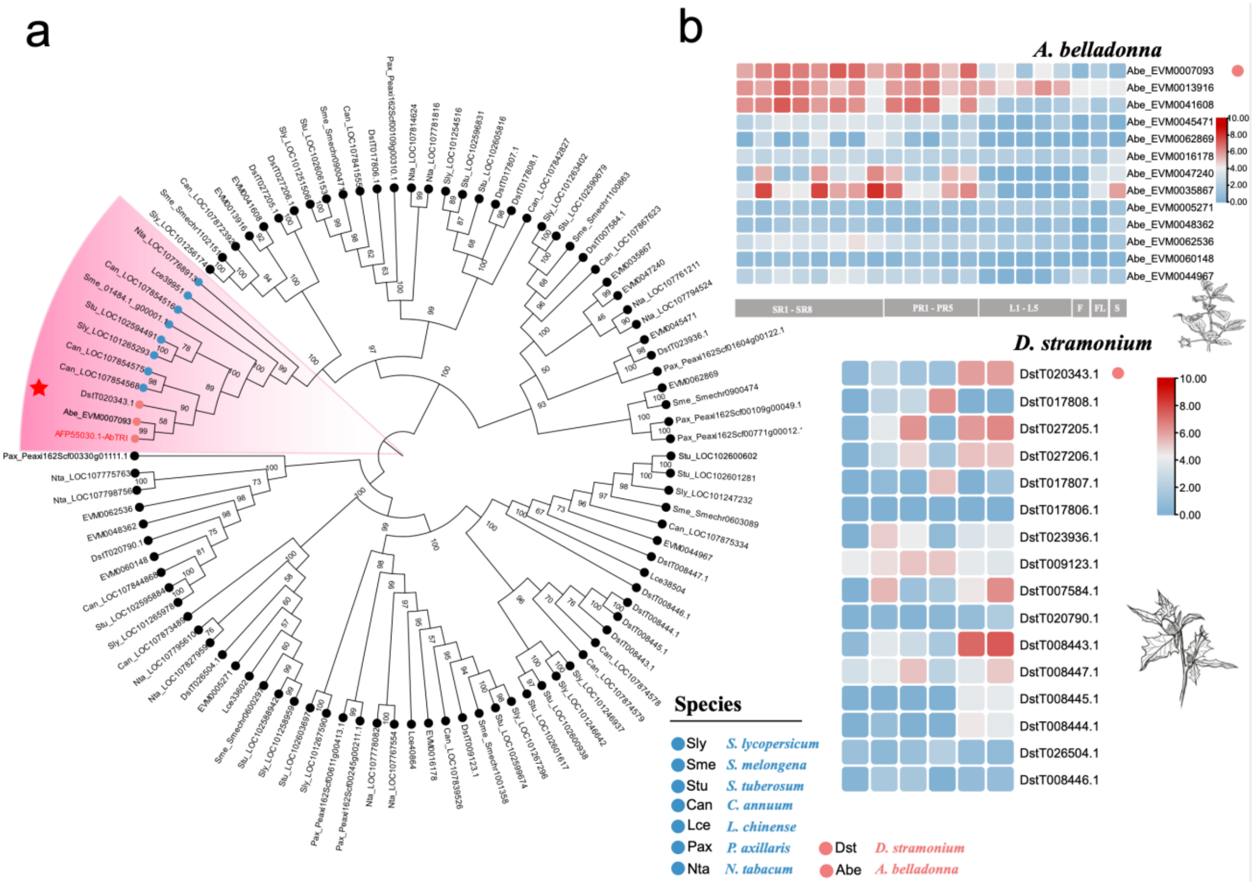


**Supplementary Fig. 19**. **Maximum-likelihood phylogeny of tropine reductase (TRI).**

**a** Maximum likelihood phylogenetic tree of TRI family. Red dots highlight the genes from species with mTAs. Blue dots highlight the genes from species without mTAs. The red font indicates the functional characterized gene downloaded from NCBI. The support value was placed on the branch with bootstrap (n=1000). **b** Gene expression profiles (in normalized TPMs) of different tissues in two species are presented in the heatmap alongside the gene names (SR: secondary roots; PR: primary roots; L: leaf; F: fruit; FL: flower; S: stem). Source data are provided as a Source Data file.

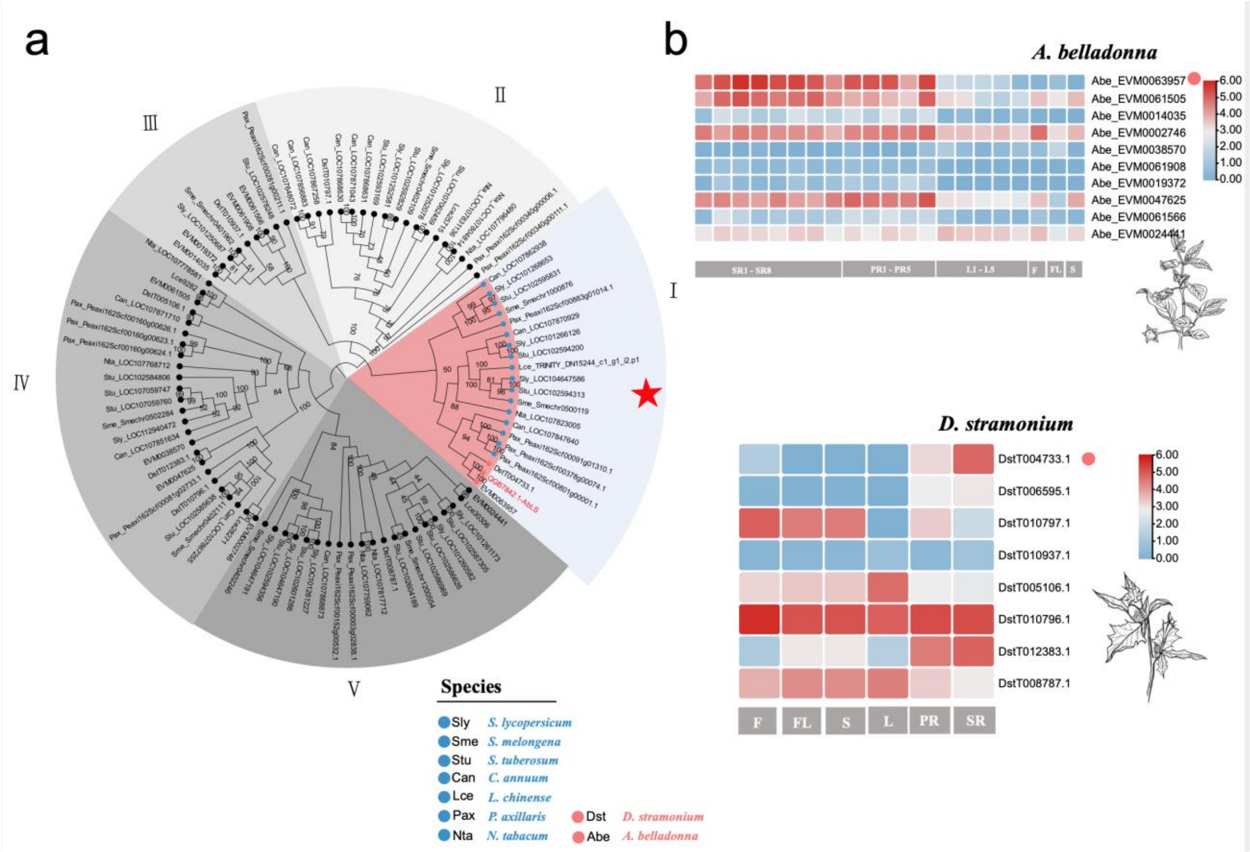


**Supplementary Fig. 20**. **Maximum-likelihood phylogeny of littorine synthase (LS).**

**a** Maximum likelihood phylogenetic tree of LS family. Red dots highlight the genes from species with mTAs. Blue dots highlight the genes from species without mTAs. The red font indicates the functional verification gene downloaded from NCBI. The support value was placed on the branch with bootstrap (n=1000). **b** Gene expression profiles (in normalized TPMs) of different tissues in two species are presented in the heatmap alongside the gene names (SR: secondary roots; PR: primary roots; L: leaf; F: fruit; FL: flower; S: stem). Source data are provided as a Source Data file.

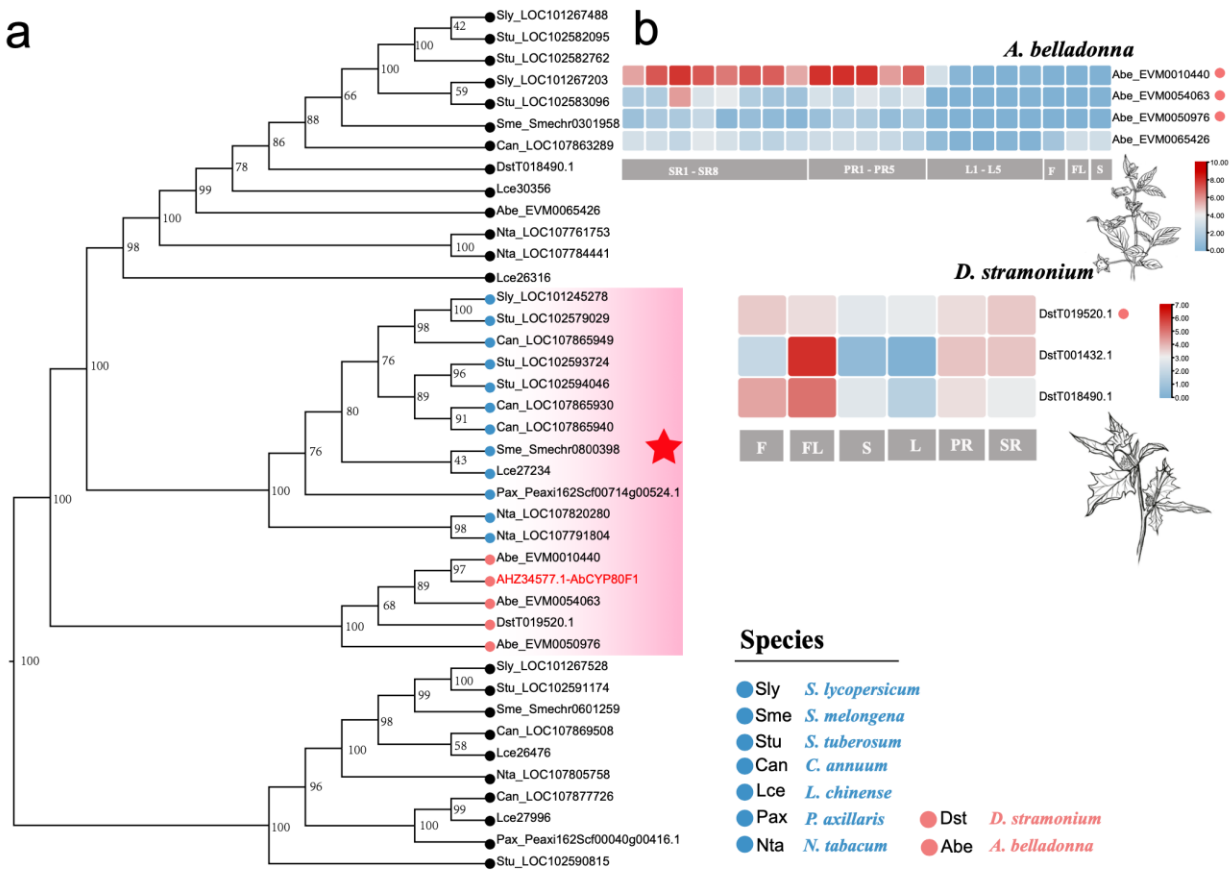


**Supplementary Fig. 21**. **Maximum-likelihood phylogeny of littorine mutase (CYP80F1).**

**a** Maximum likelihood phylogenetic tree of CYP80F1 family. Red dots highlight the genes from species with mTAs. Blue dots highlight the genes from species without mTAs. The red font indicates the functional characterized gene downloaded from NCBI. The support value was placed on the branch with bootstrap (n=1000). **b** Gene expression profiles (in normalized TPMs) of different tissues in two species are presented in the heatmap alongside the gene names (SR: secondary roots; PR: primary roots; L: leaf; F: fruit; FL: flower; S: stem). Source data are provided as a Source Data file.

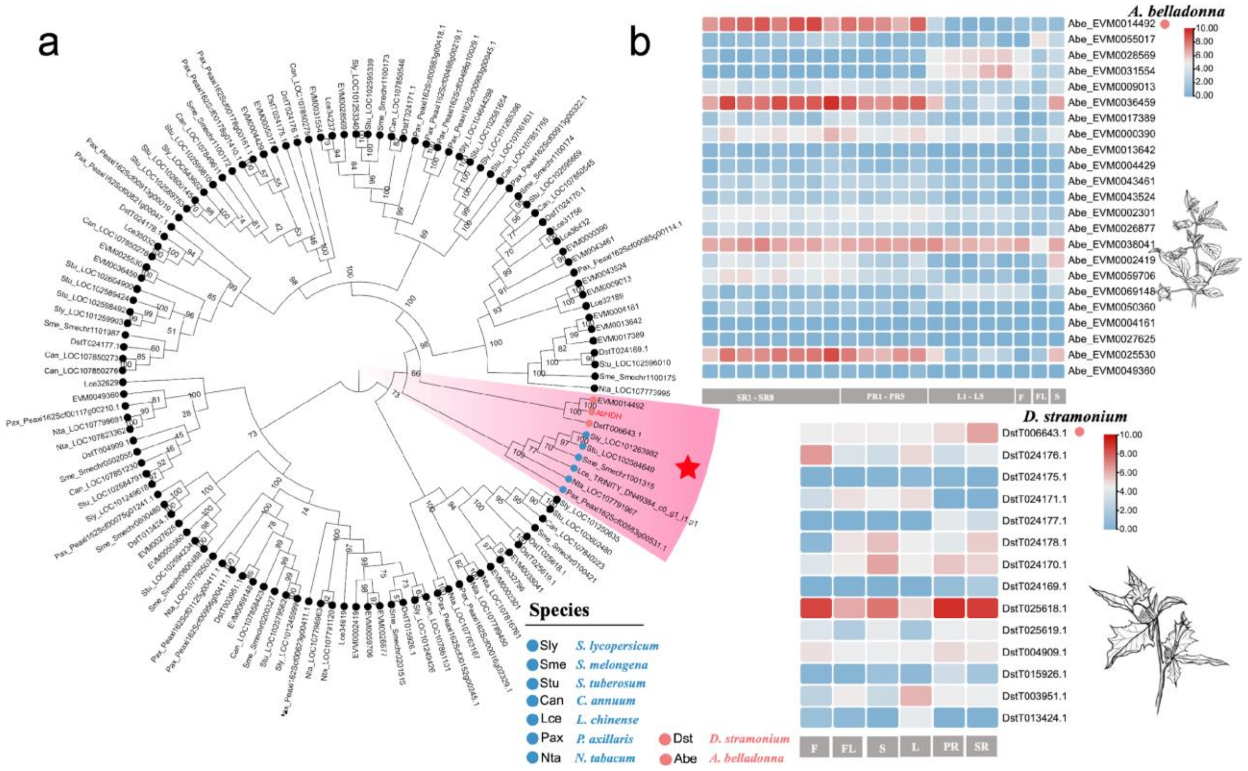


**Supplementary Fig. 22. Maximum-likelihood phylogeny of hyoscyamine dehydrogenase (HDH). a** Maximum likelihood phylogenetic tree of HDH family. Red dots highlight the genes from species with mTAs. Blue dots highlight the genes from species without mTAs. The red font indicates the functional characterized gene downloaded from NCBI. **b** Gene expression profiles (in normalized TPMs) of different tissues in two species are presented in the heatmap alongside the gene names (SR: secondary roots; PR: primary roots; L: leaf; F: fruit; FL: flower; S: stem). Source data are provided as a Source Data file.

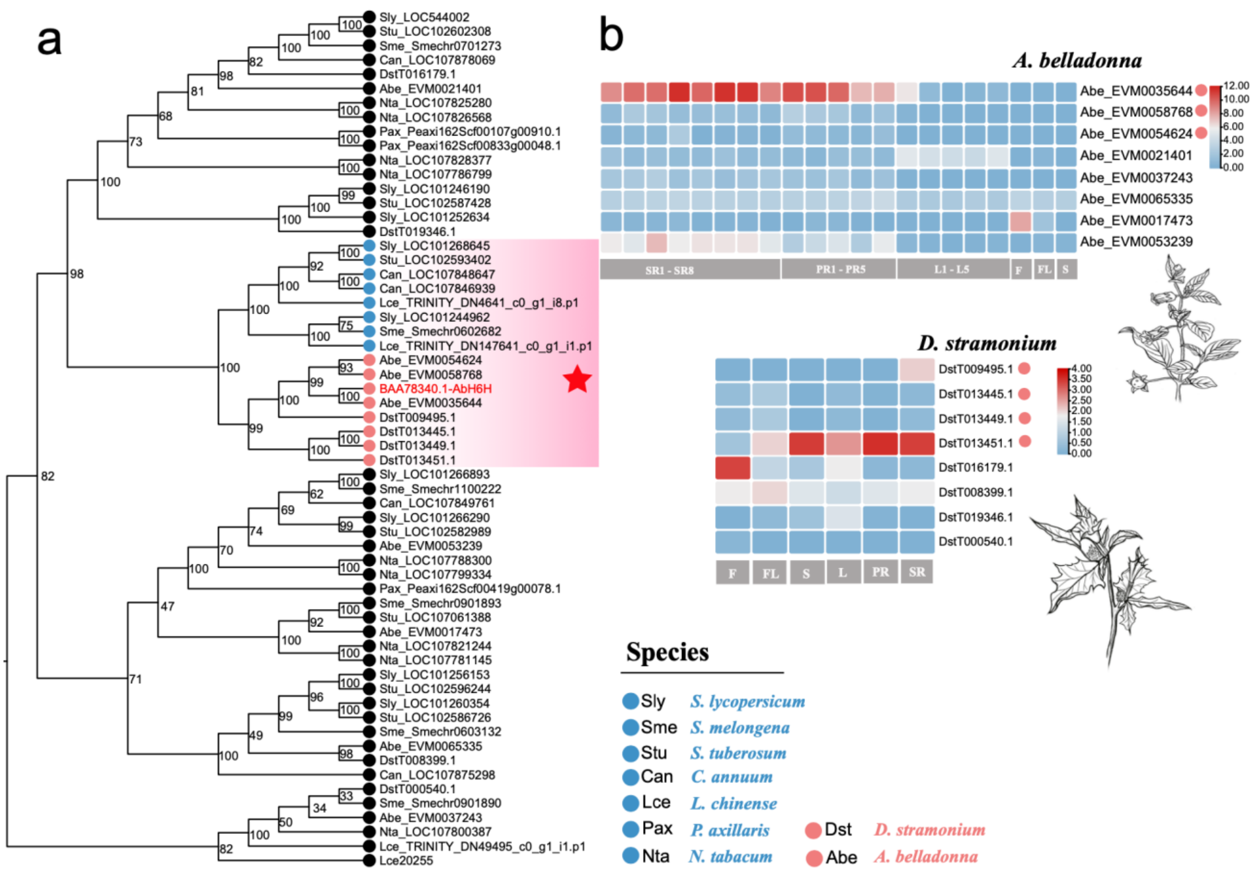


**Supplementary Fig. 23. Maximum-likelihood phylogeny of hyoscyamine 6b-hydroxylase (H6H). a** Maximum likelihood phylogenetic tree of H6H family. Red dots highlight the genes from species with mTAs. Blue dots highlight the genes showing species without mTAs. The red font indicates the functional characterized gene downloaded from NCBI. The support value was placed on the branch with bootstrap (n=1000). **b** Gene expression profiles (in normalized TPMs) of different tissues in two species are presented in the heatmap alongside the gene names (SR: secondary roots; PR: primary roots; L: leaf; F: fruit; FL: flower; S: stem). Source data are provided as a Source Data file.

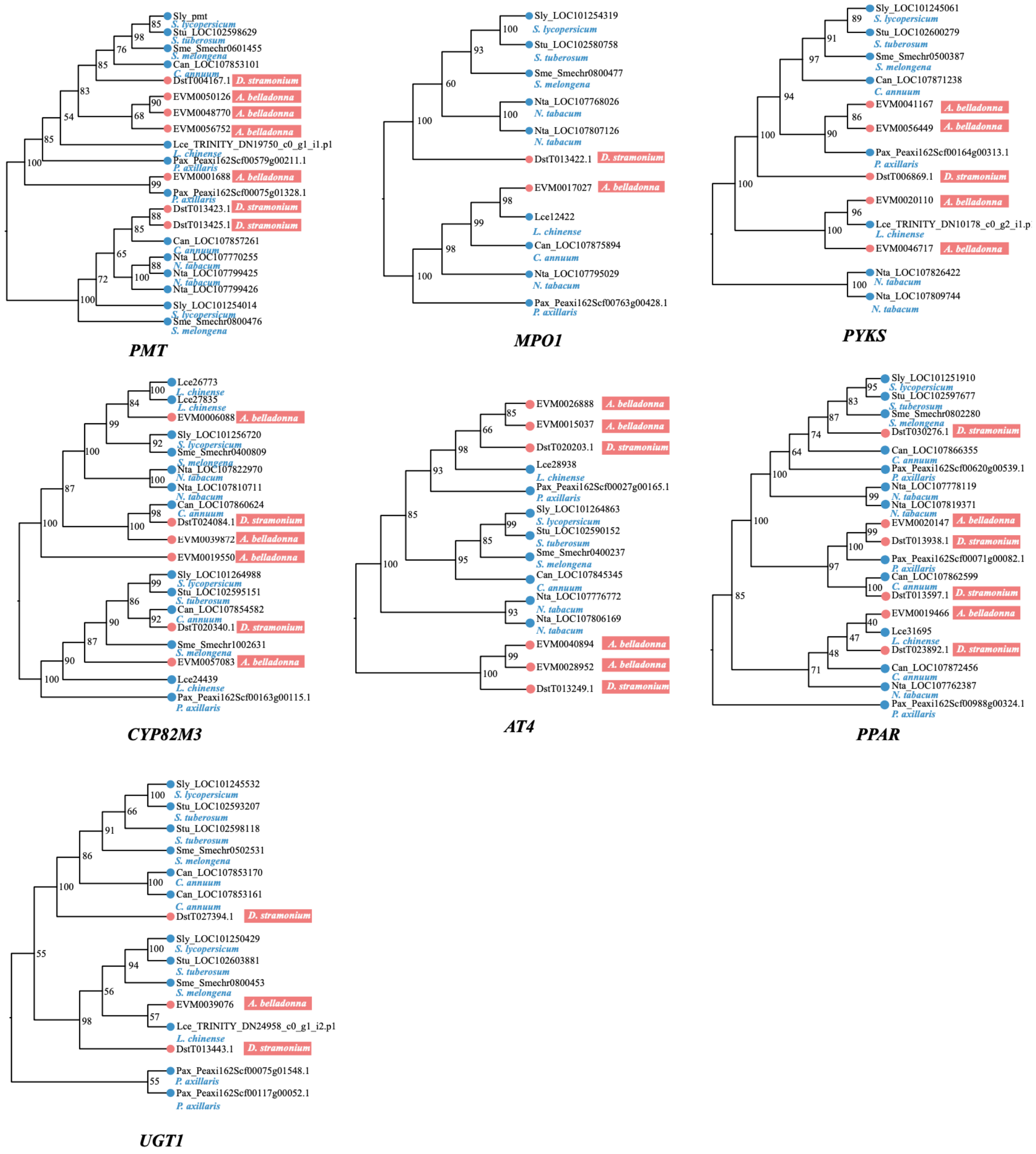


**Supplementary Fig. 24. Gene trees of TAs pathway genes in nine Solanaceae species.** Maximum likelihood phylogenetic tree of each TAs biosynthetic gene in nine Solanaceae species, including *N. tabacum*, *P. axillaris*, *A. belladonna*, *L. chinense*, D. *stramonium*, *C. annuum*, *S. lycopersicum*, *S. tuberosum*, and *S. melongena*. The support value was placed on the branch with bootstrap (n=1000). The genes from *A. belladonna* and *D. stramonium* were highlighted with red.

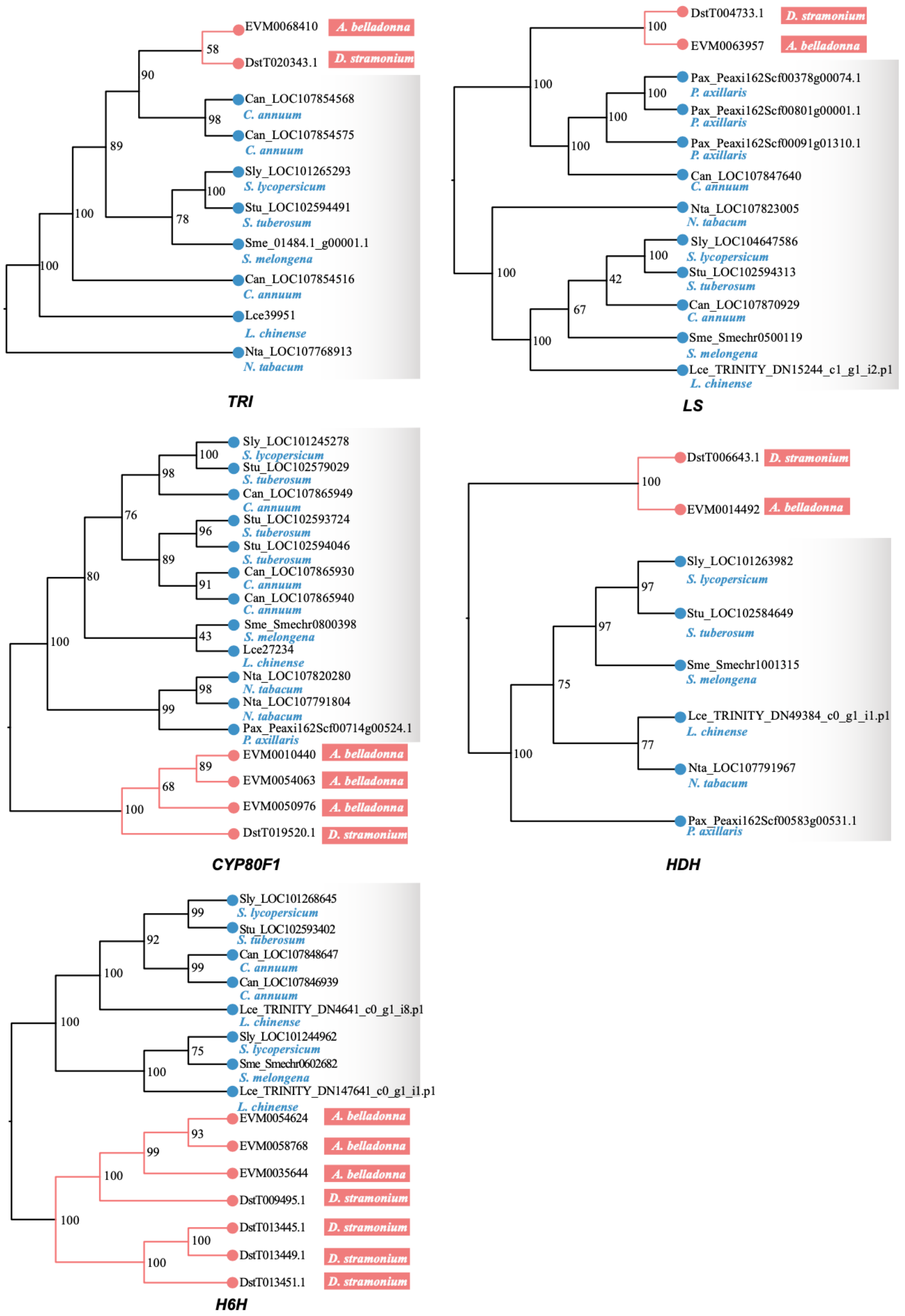


**Supplementary Fig. 25. Gene trees of mTAs pathway genes in nine Solanaceae species.** Maximum likelihood phylogenetic tree of each mTAs biosynthetic genes in nine Solanaceae species, including *N. tabacum*, *P. axillaris*, *A. belladonna*, *L. chinense*, *D. stramonium*, *C. annuum*, *S. lycopersicum*, *S. tuberosum*, and *S. melongena*. The support value was placed on the branch with bootstrap (n=1000). The genes from *A. belladonna* and *D. stramonium* were highlighted with red.

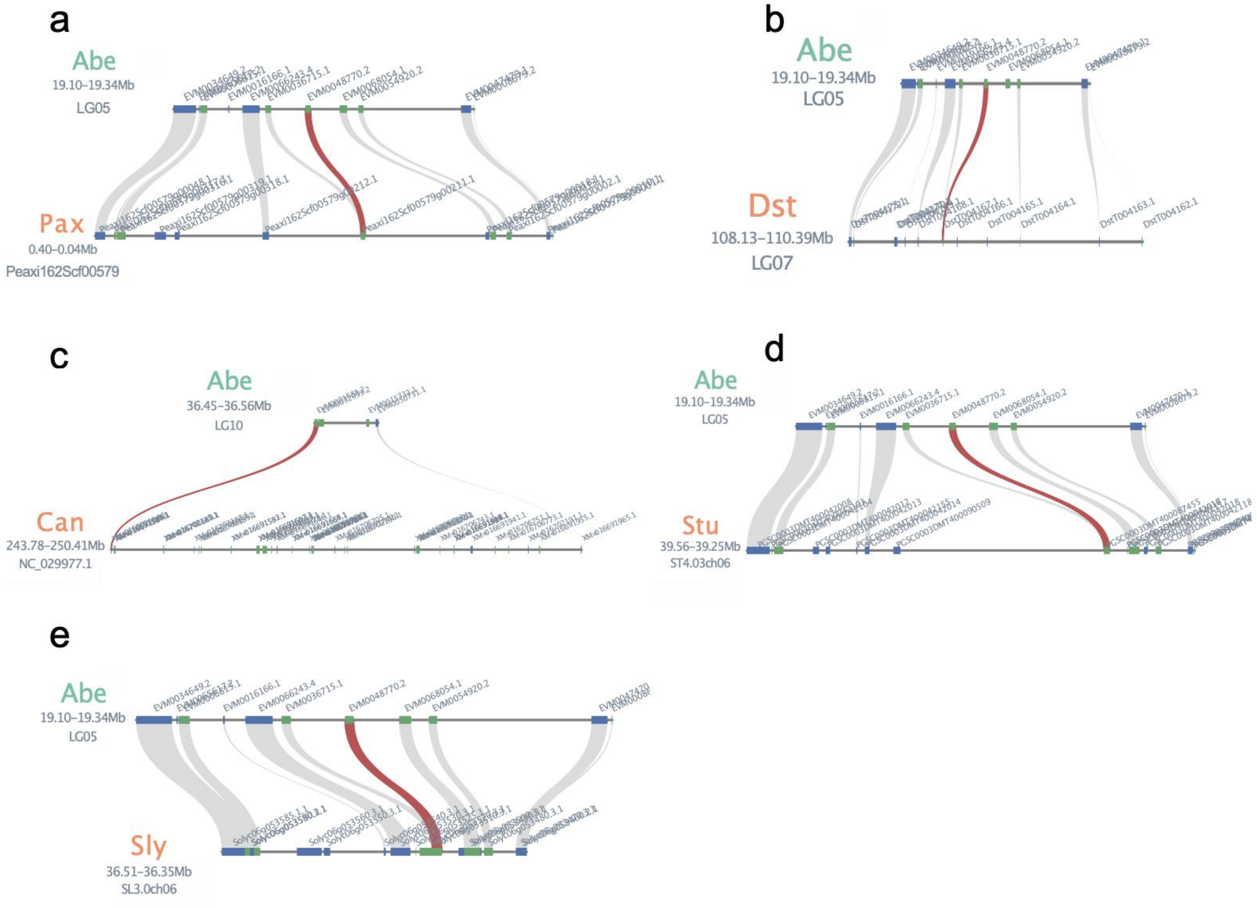


**Supplementary Fig. 26. Microsynteny analysis of *PMT* genes between *A. belladonna* and other species in Solanaceae**. The species used in this analysis were *P. axillaris* (Pax, **a**), *D. stramonium* (Dst, **b**), *C. annuum* (Can, **c**), *S. tuberosum* (Stu, **d**), *S. lycopersicum* (Sly, **e**). The syntenic *PMT* gene were highlighted with red. The genomic region and chromosome ID were placed under the abbreviation of species name.

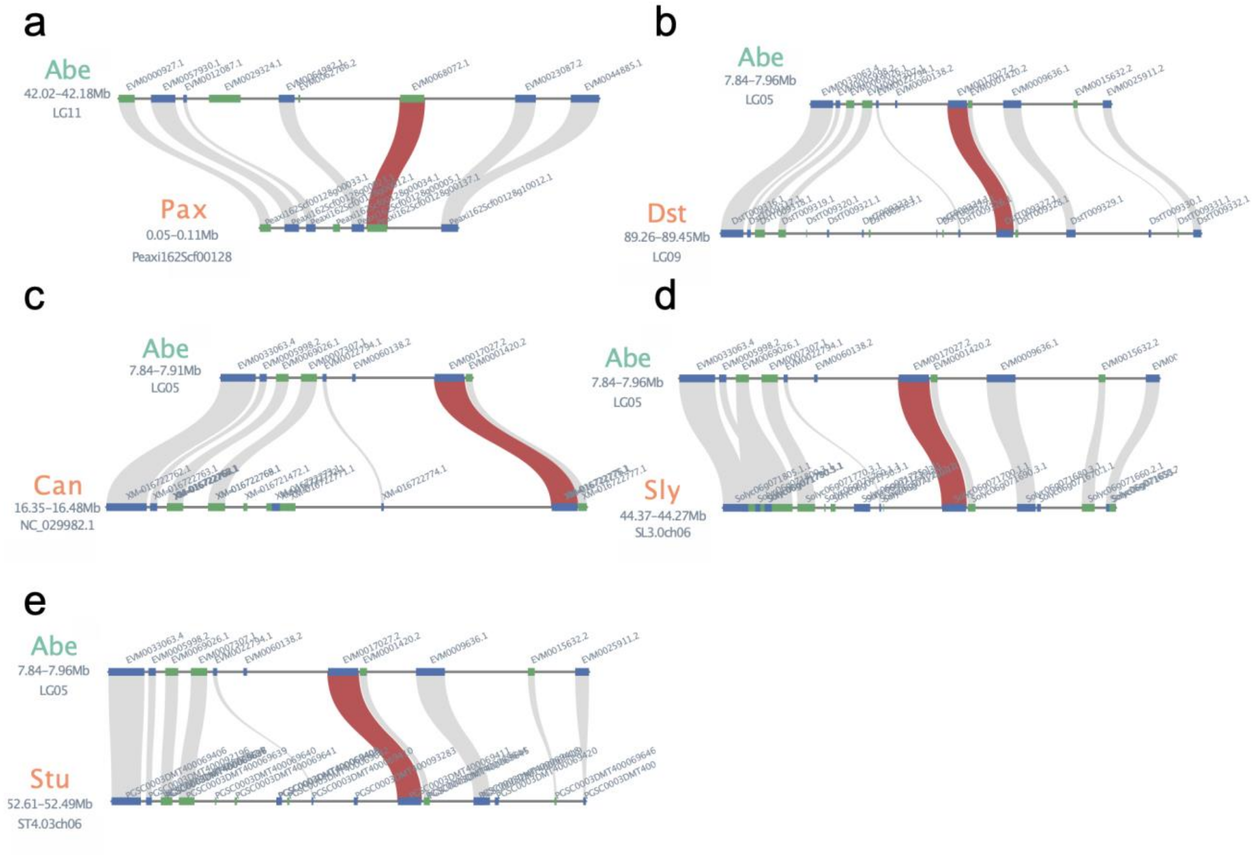


**Supplementary Fig. 27. Microsynteny analysis of *MPO* genes between *A. belladonna* and other species in Solanaceae**. The species used in this analysis were *P. axillaris* (Pax, **a**), *D. stramonium* (Dst, **b**), *C. annuum* (Can, **c**), *S. lycopersicum* (Sly, **d**), *S. tuberosum* (Stu, **e**). The syntenic *MPO* genes were highlighted with red. The genomic region and chromosome ID were placed under the abbreviation of species name.

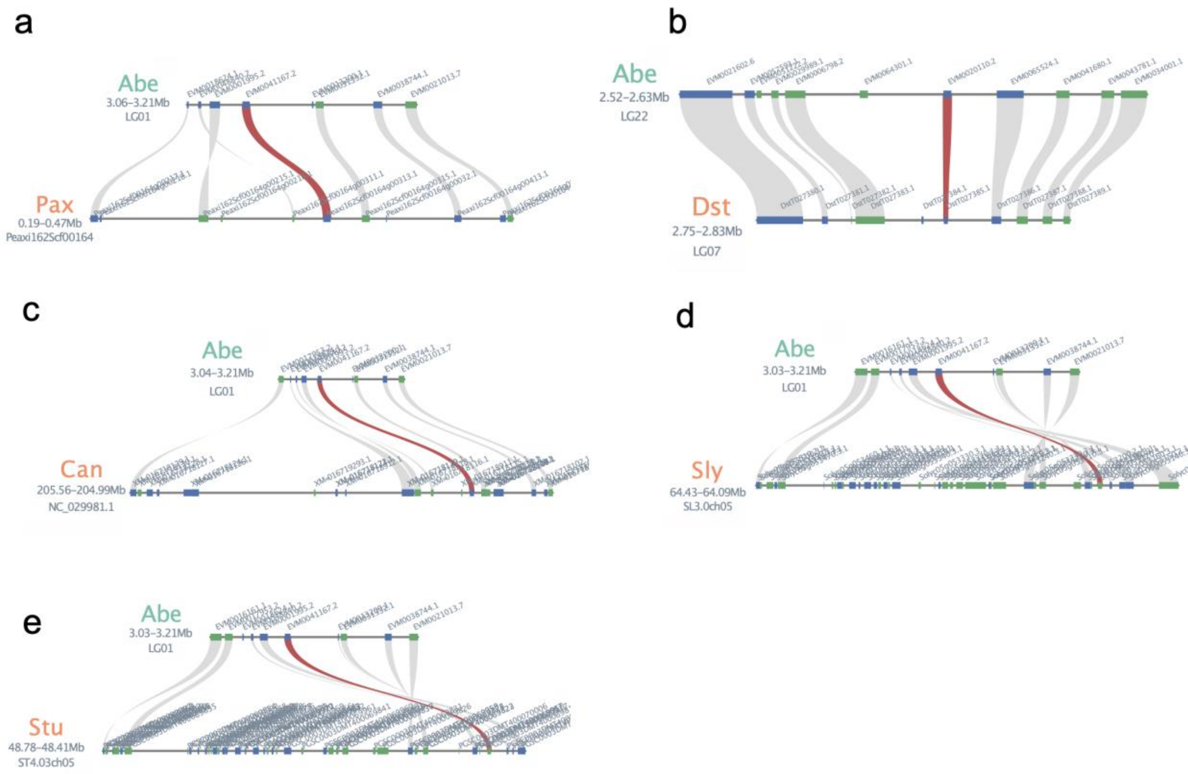


**Supplementary Fig. 28. Microsynteny analysis of *PYKS* genes between *A. belladonna* and other species in Solanaceae**. The species used in this analysis were *P. axillaris* (Pax, **a**), *D. stramonium* (Dst, **b**), *C. annuum* (Can, **c**), *S. lycopersicum* (Sly, **d**), *S. tuberosum* (Stu, **e**). The syntenic *PYKS* genes were highlighted with red. The genomic region and chromosome ID were placed under the abbreviation of species names.

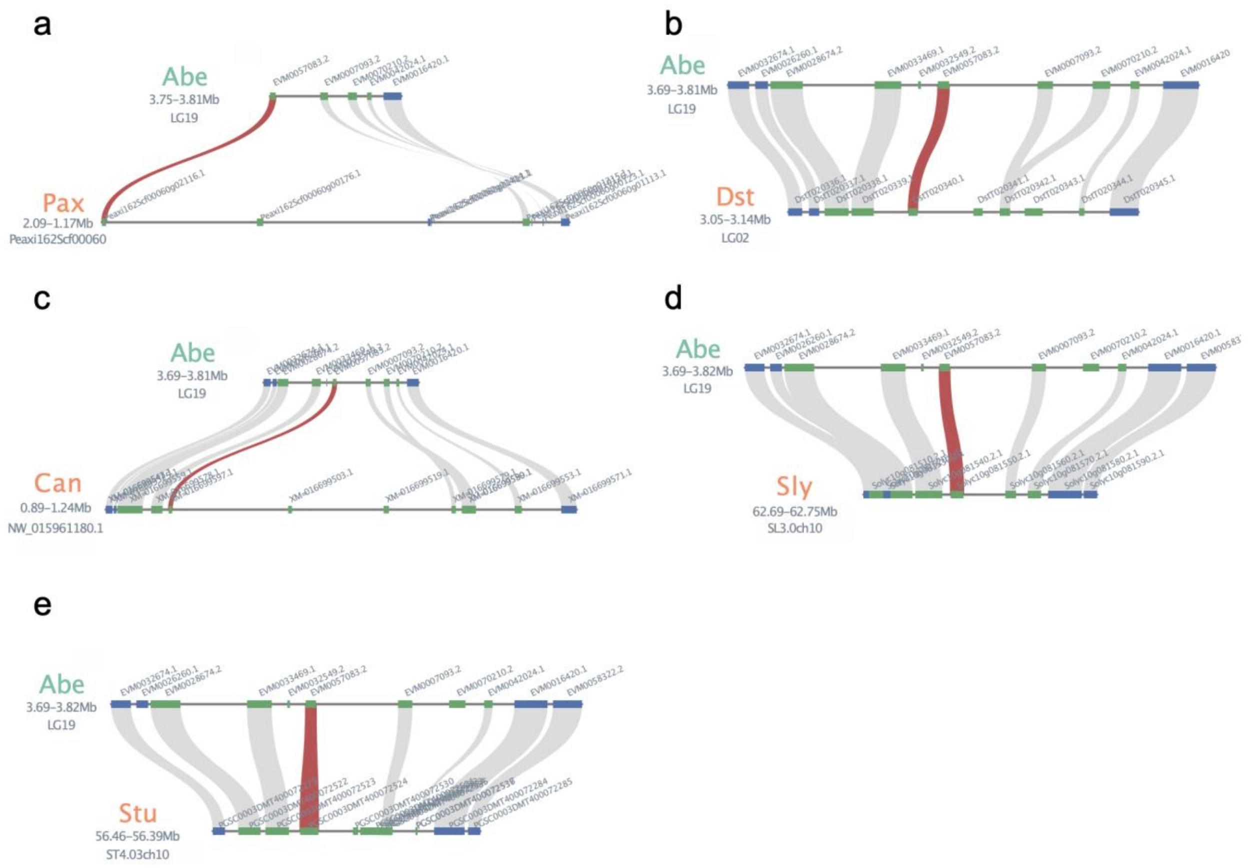


**Supplementary Fig. 29. Microsynteny analysis of *CYP82M3* genes between *A. belladonna* and other species in Solanaceae**. The species used in this analysis were *P. axillaris* (Pax, **a**), *D. stramonium* (Dst, **b**), *C. annuum* (Can, **c**), *S. lycopersicum* (Sly, **d**), *S. tuberosum* (Stu, **e**). The syntenic *CYP82M3* genes were highlighted with red. The genomic region and chromosome ID were placed under the abbreviation of species names.

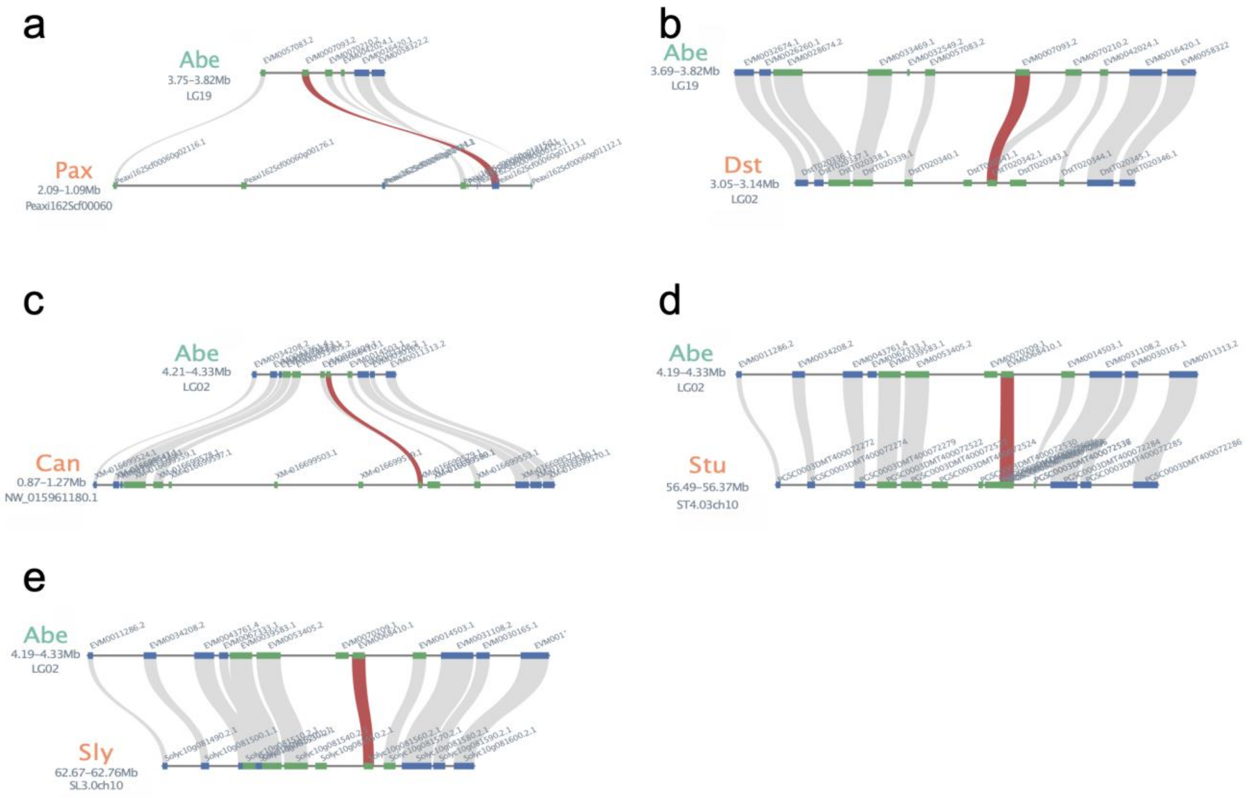


**Supplementary Fig. 30. Microsynteny analysis of *TRI* genes between *A. belladonna* and other species in Solanaceae**. The species used in this analysis were *P. axillaris* (Pax, **a**), *D. stramonium* (Dst, **b**), *C. annuum* (Can, **c**), *S. tuberosum* (Stu, **d**), *S. lycopersicum* (Sly, **e**). The syntenic *TRI* genes were highlighted with red. The genomic region and chromosome ID were placed under the abbreviation of species names.

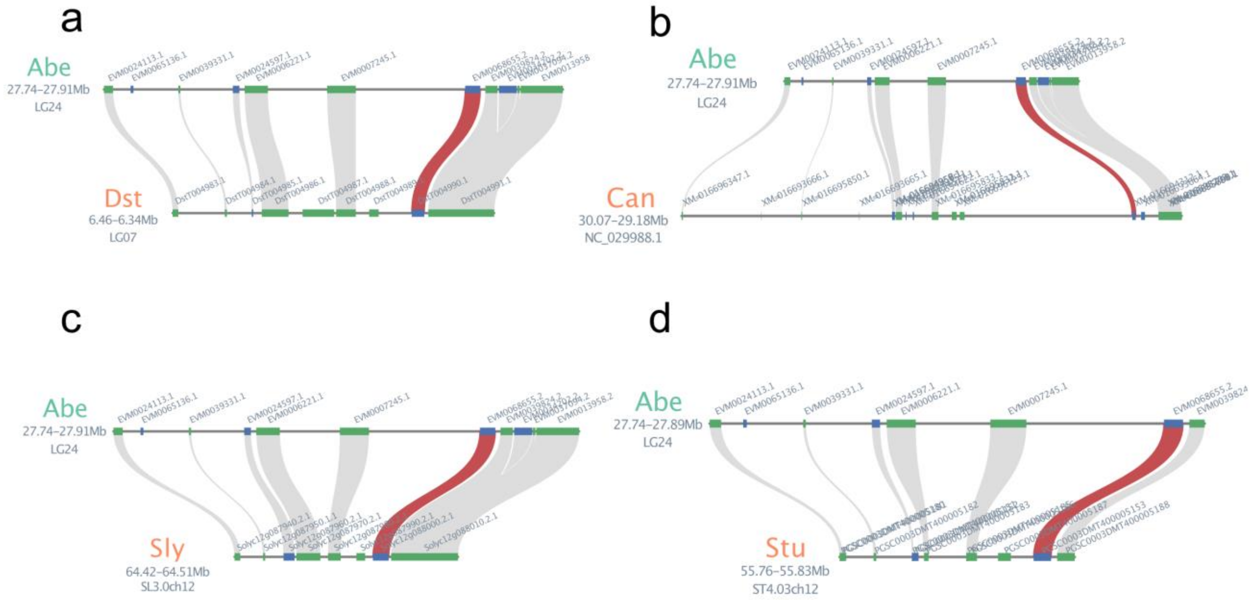


**Supplementary Fig. 31. Microsynteny analysis of *AT4* genes between *A. belladonna* and other species in Solanaceae**. The species used in this analysis were *D. stramonium* (Dst, **a**), *C. annuum* (Can, **b**), *S. lycopersicum* (Sly, **c**) and *S. tuberosum* (Stu, **d**). The syntenic *AT4* genes were highlighted with red. The genomic region and chromosome ID were placed under the abbreviation of species names.

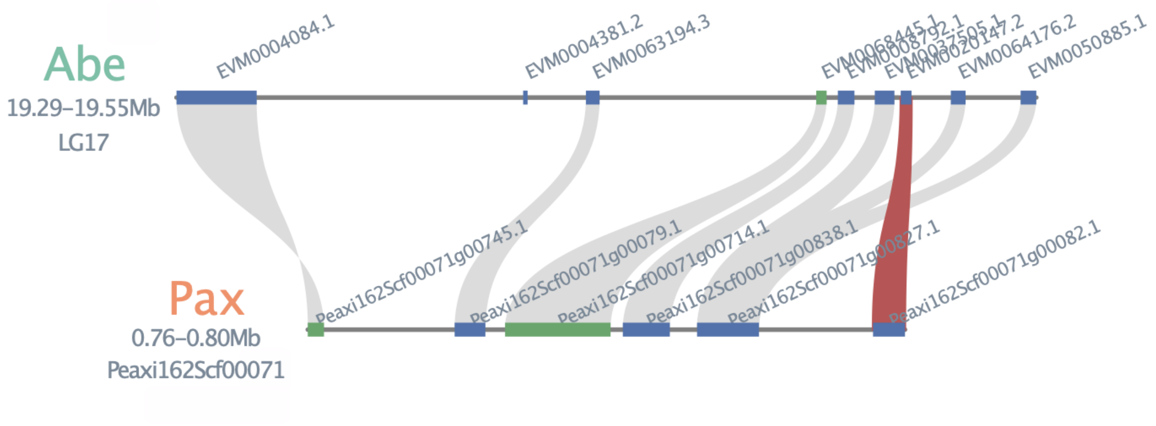


**Supplementary Fig. 32. Microsynteny analysis of *PPAR* genes between *A. belladonna* (Abe) and *P. axillaris* (Pax).** The syntenic *PPAR* genes were highlighted with red. The genomic region and chromosome ID were placed under the abbreviation of species names.

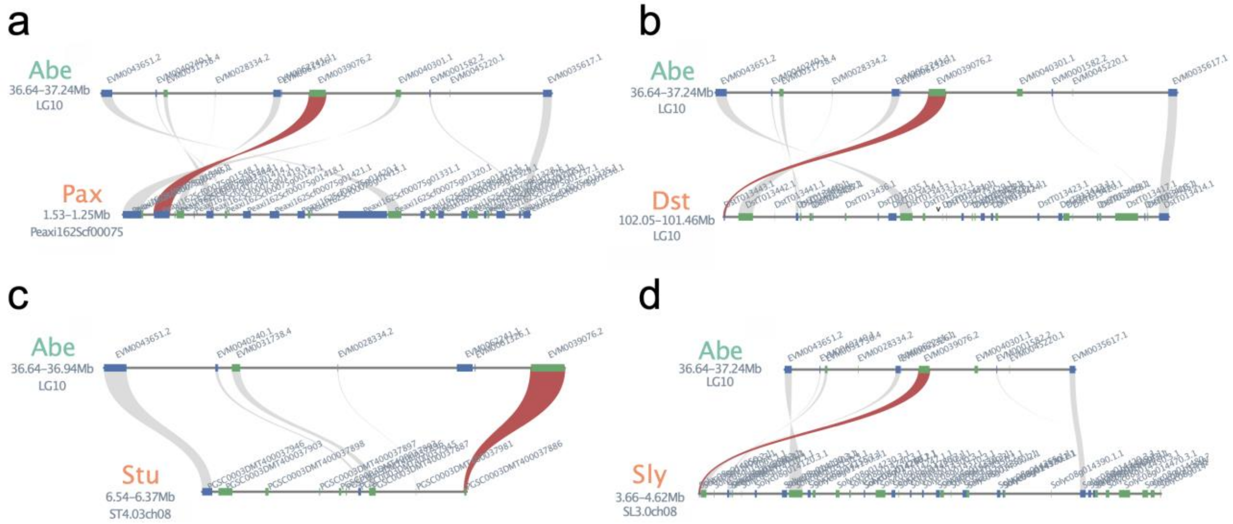


**Supplementary Fig. 33. Microsynteny analysis of *UGT* genes between *A. belladonna* and other species in Solanaceae**. The species used in this analysis are *P. axillaris* (Pax, **a**), *D. stramonium* (Dst, **b**), *S. tuberosum* (Stu, **c**), *S. lycopersicum* (Sly, **d**). The syntenic *UGT* genes were highlighted with red. The genomic region and chromosome ID were placed under the abbreviation of species names.

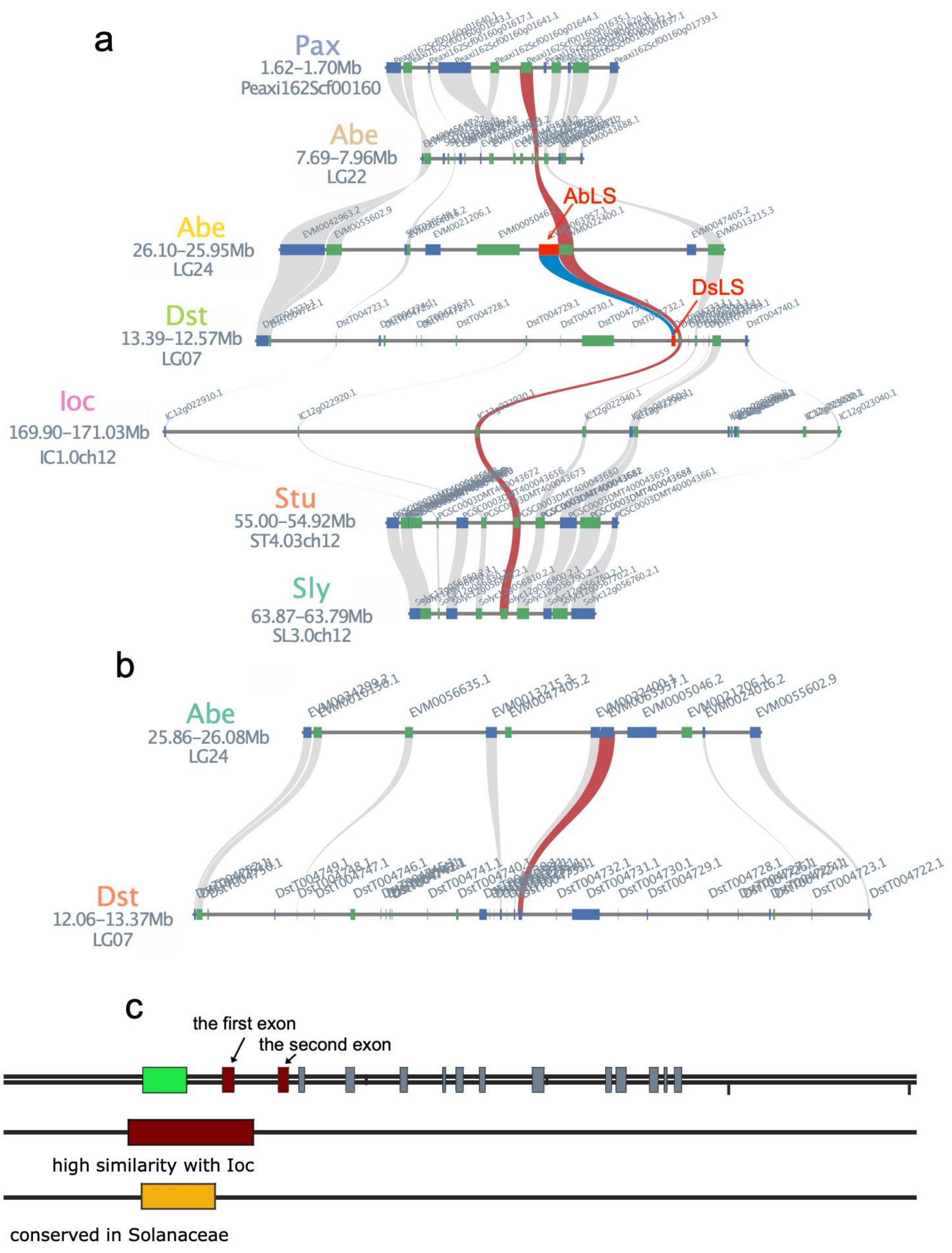


**Supplementary Fig. 34. Microsynteny analysis of *LS* genes between *A. belladonna* and other species in Solanaceae**. **a** the microsynteny analysis of *LS* genes in Solanaceae, the red lines represent a gene, *EVM0022400.1*, that is very close to the *AbLS* gene in *A. belladonna* chromosome (495 bp up-stream of the translation initiation site of *AbLS*). **b** The microsynteny analysis of *LS* genes between *A. belladonna* and *D. stramonium*. **c** The gene structure and syntenic region of

*AbLS*. The green rectangle represents the second exon of *EVM0022400.1*; the red rectangles represent the syntenic region between *A. belladonna* and *Iochroma cyaneum*; the yellow rectangle represents syntenic region between *A. belladonna* and other species in Solanaceae, including *P. axillaris*, *P. inflata*, *D. stramonium*, *S. tuberosum*, and *S. lycopersicum*.

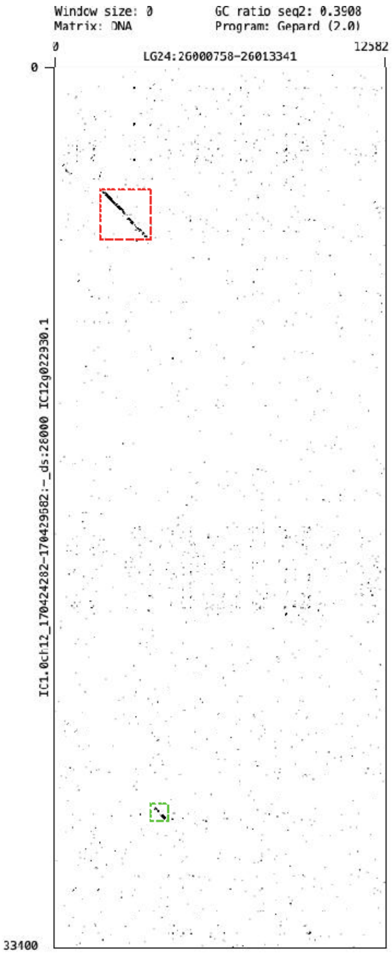


**Supplementary Fig. 35. The dot plot of *LS* region between *Atropa belladonna* and *Iochroma cyaneum***. The red box represented the portion of *AbLS* promoter region and the first exon of *AbLS*; the green box represented the second exon of *AbLS*.

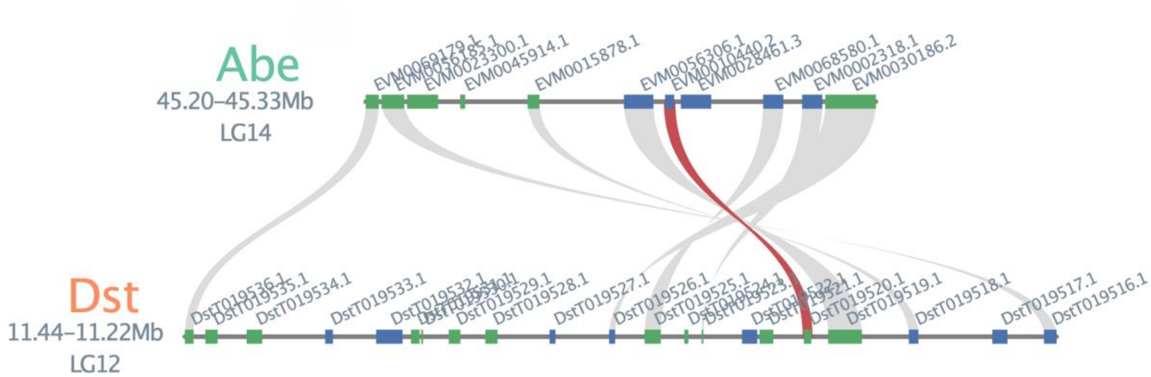


**Supplementary Fig. 36. Microsynteny analysis of *CYP80F1* genes between *A. belladonna* and *D. stramonium* (Dst)**. The syntenic *CYP80F1* genes were highlighted with red. The genomic region and chromosome ID were placed under the abbreviation of species names.

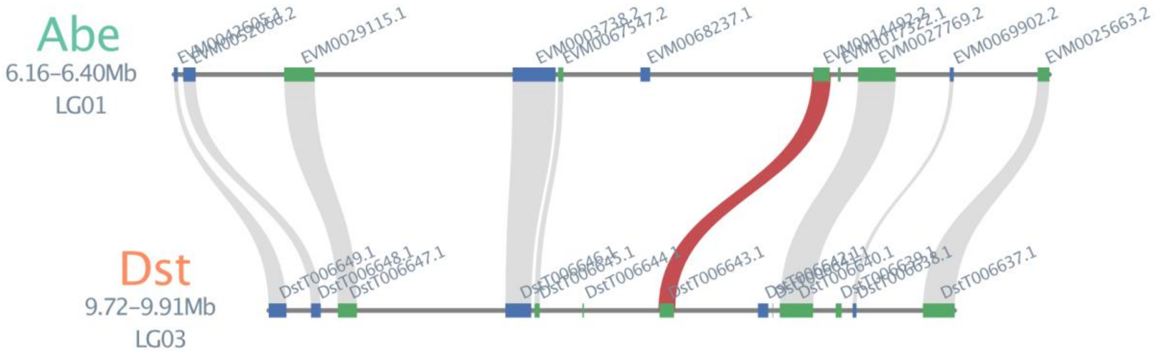


**Supplementary Fig. 37. Microsynteny analysis of *HDH* genes between *A. belladonna* and *D. stramonium* (Dst)**. The syntenic *HDH* genes were highlighted with red. The genomic region and chromosome ID were placed under the abbreviation of species names.

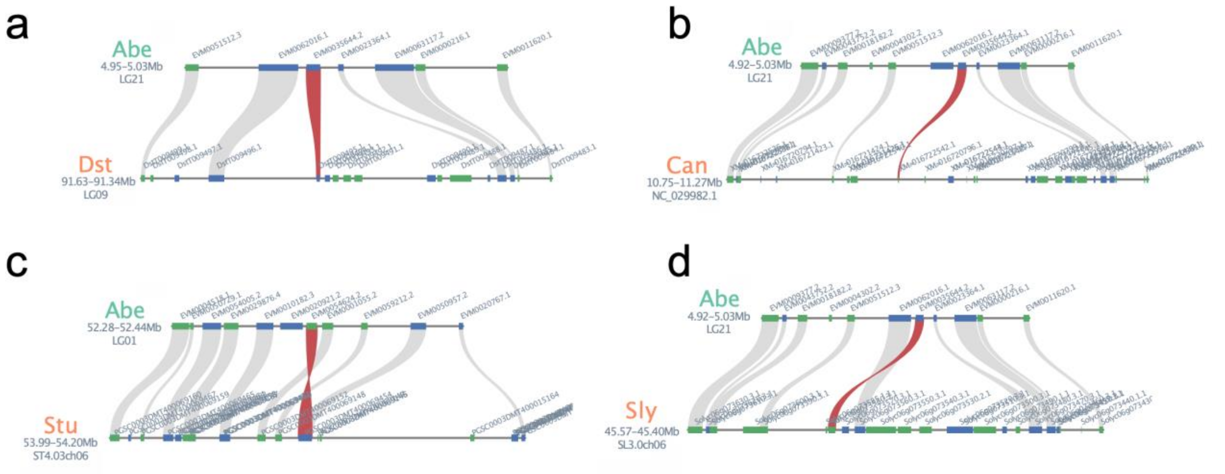


**Supplementary Fig. 38. Microsynteny analysis of *H6H* genes between *A. belladonna* and other species in Solanaceae**. The species used in this analysis were *D. stramonium* (Dst, **a**), *C. annuum* (Can, **b**), *S. tuberosum* (Stu, **c**), *S. lycopersicum* (Sly, **d**). The syntenic *H6H* genes were highlighted with red. The genomic region and chromosome ID were placed under the abbreviation of species names.

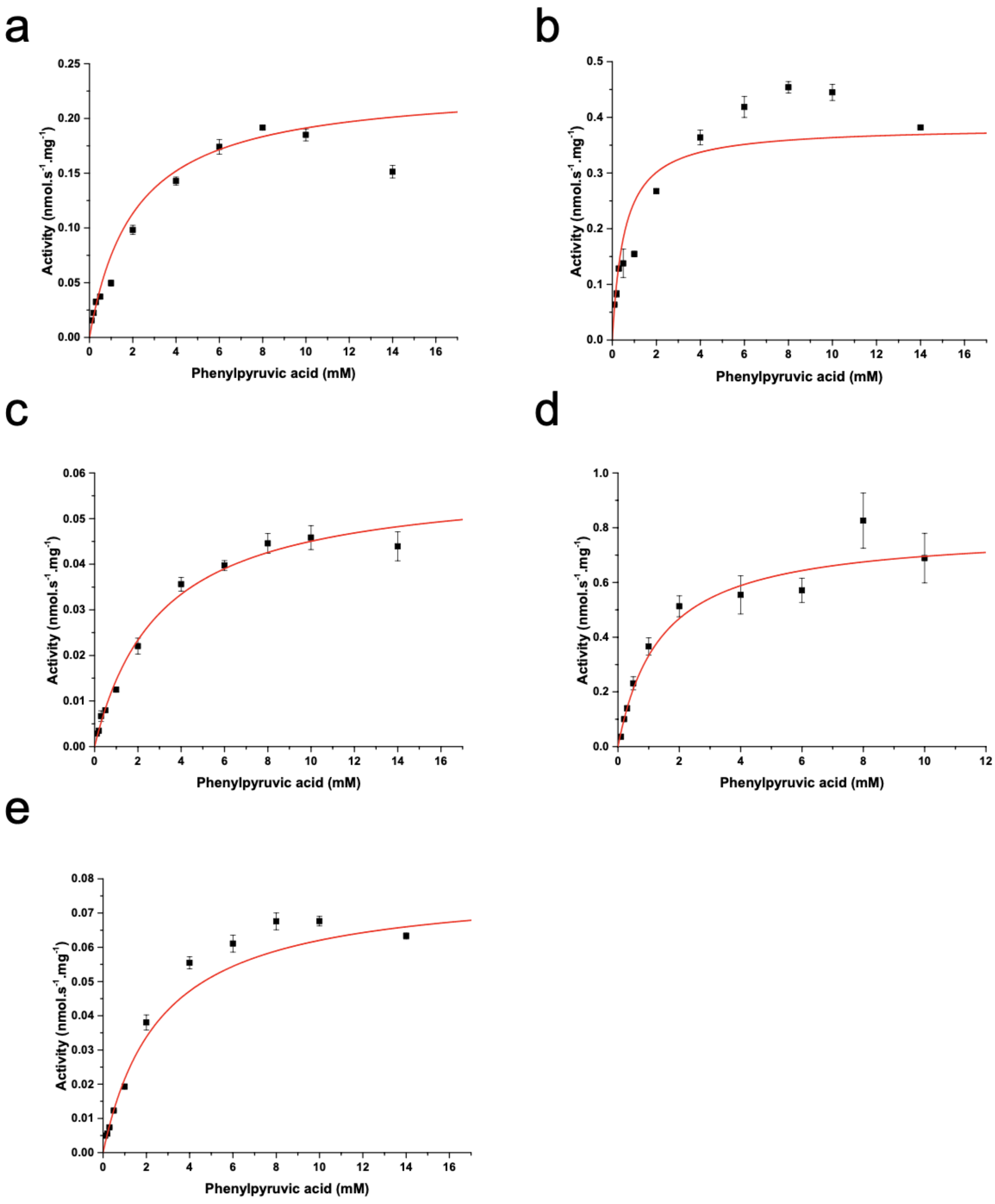


**Supplementary Fig. 39. Michaelis–Menten curves for PPARs.** The Michaelis–Menten curves of the AbPPAR (**a**), DsPPAR (**b**), CaPPAR (**c**), SlPPAR (**d**), and PaPPAR (**e**) were presented. The buffer for the reduction reaction was potassium phosphate (50 mM, pH 8.0). Phenylpyruvic acid was used as substrates. The data are presented as means values +/- s.d. (n = 3 biologically independent samples). Source data are provided as a Source Data file.

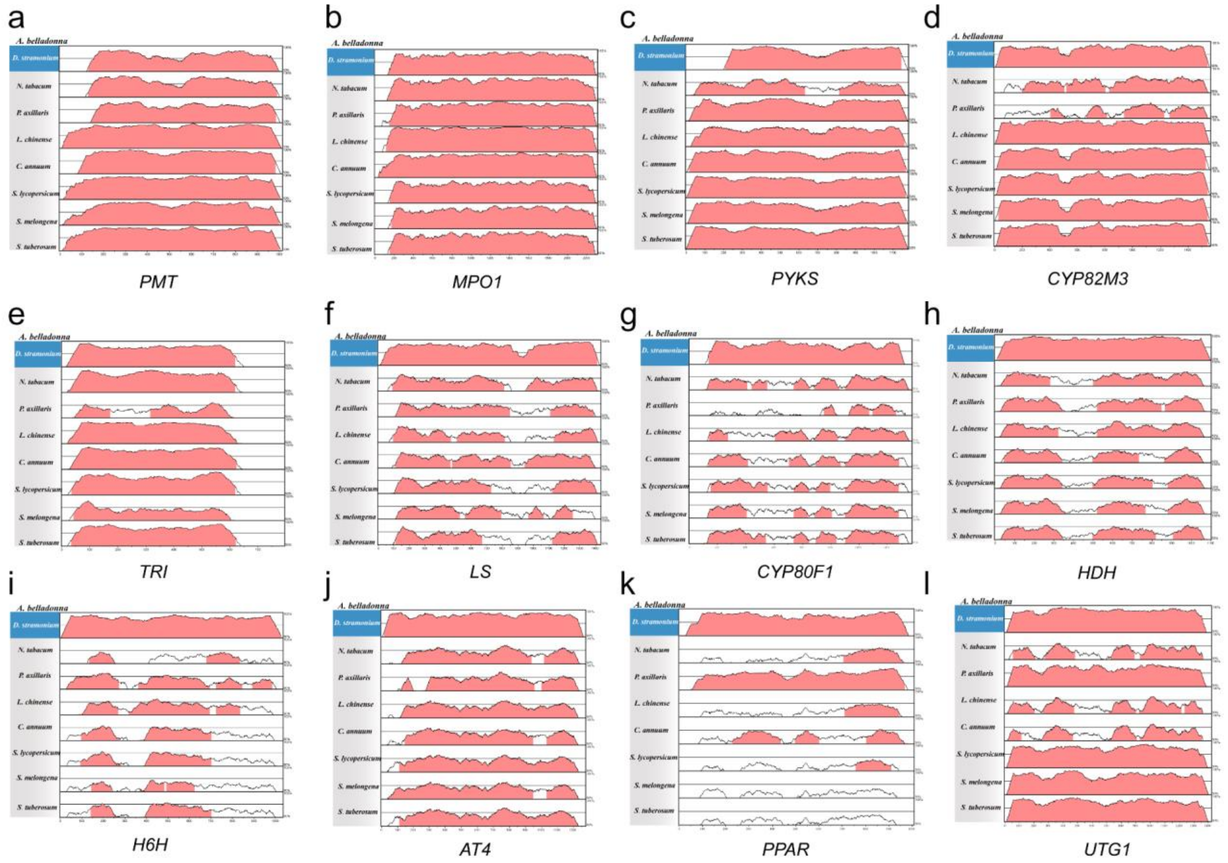


**Supplementary Fig. 40. Pairwise comparisons and sequence identity between mTAs biosynthetic genes from *A. belladonna* and that from other eight Solanaceae using VISTA[2].** The genes analyzed in this figure include *PMT* (**a**), *MPO1* (**b**), *PYKS* (**c**), *CYP82M3* (**d**), *TRI* (**e**), *LS* (**f**), *CYP80F1* (**g**), *HDH* (**h**), *H6H* (**i**), *AT4* (**j**), *PPAR* (**k**) and *UGT1* (**l**). The species used in this figure include *D. stramonium*, *N. tabacum*, *P*, *axillaris*, *L*, *Chinense*, *C. annum*, *S. lycopersicum*, *S. melongena*, and *S. tuberosum*. *D. stramonium* were highlighted with blue.

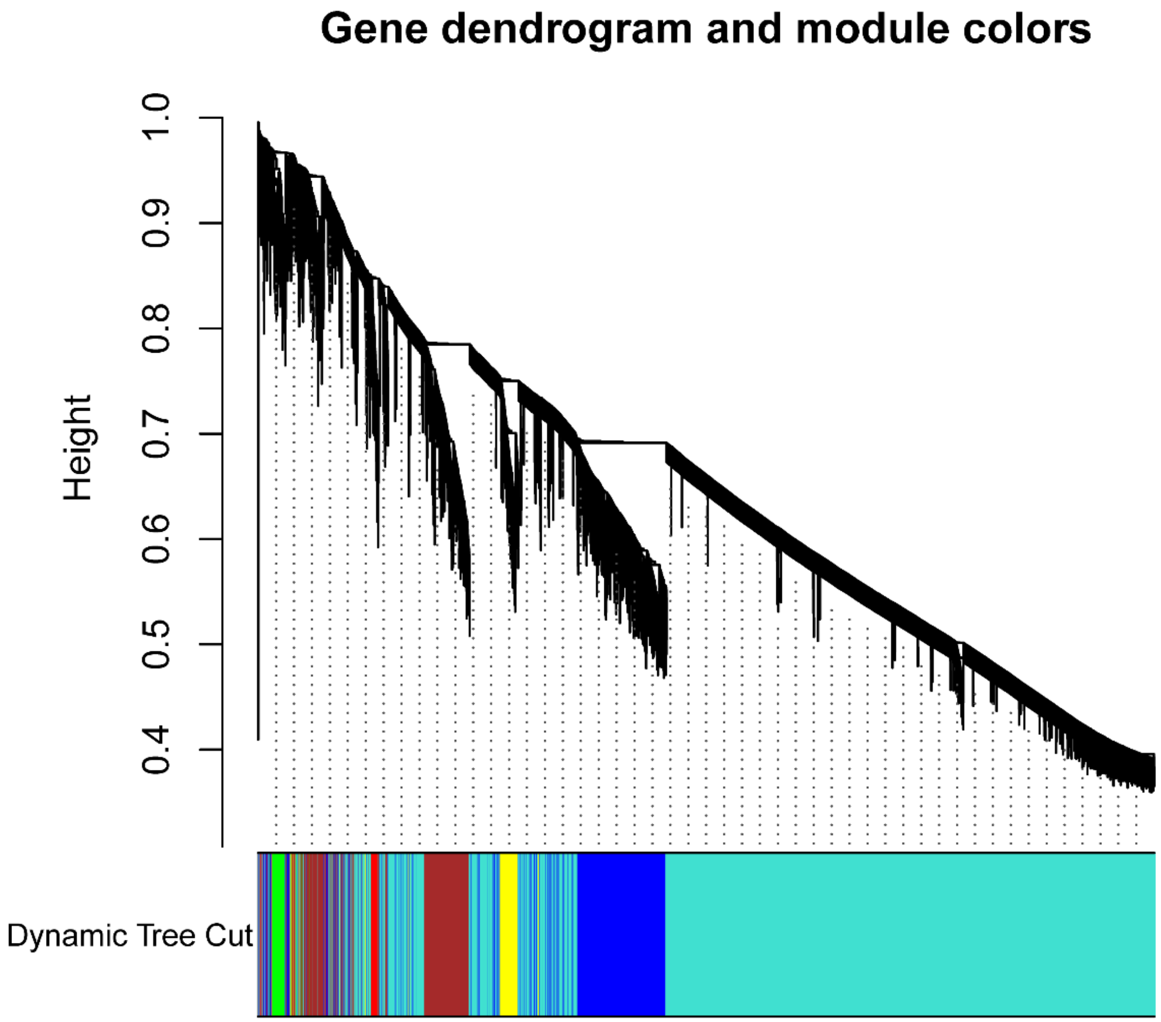


**Supplementary Fig. 41. WGCNA analysis of differential gene expression in *A. belladonna*.** The different colours under the dendrogram show co-expressed modules identified using WGCNA.

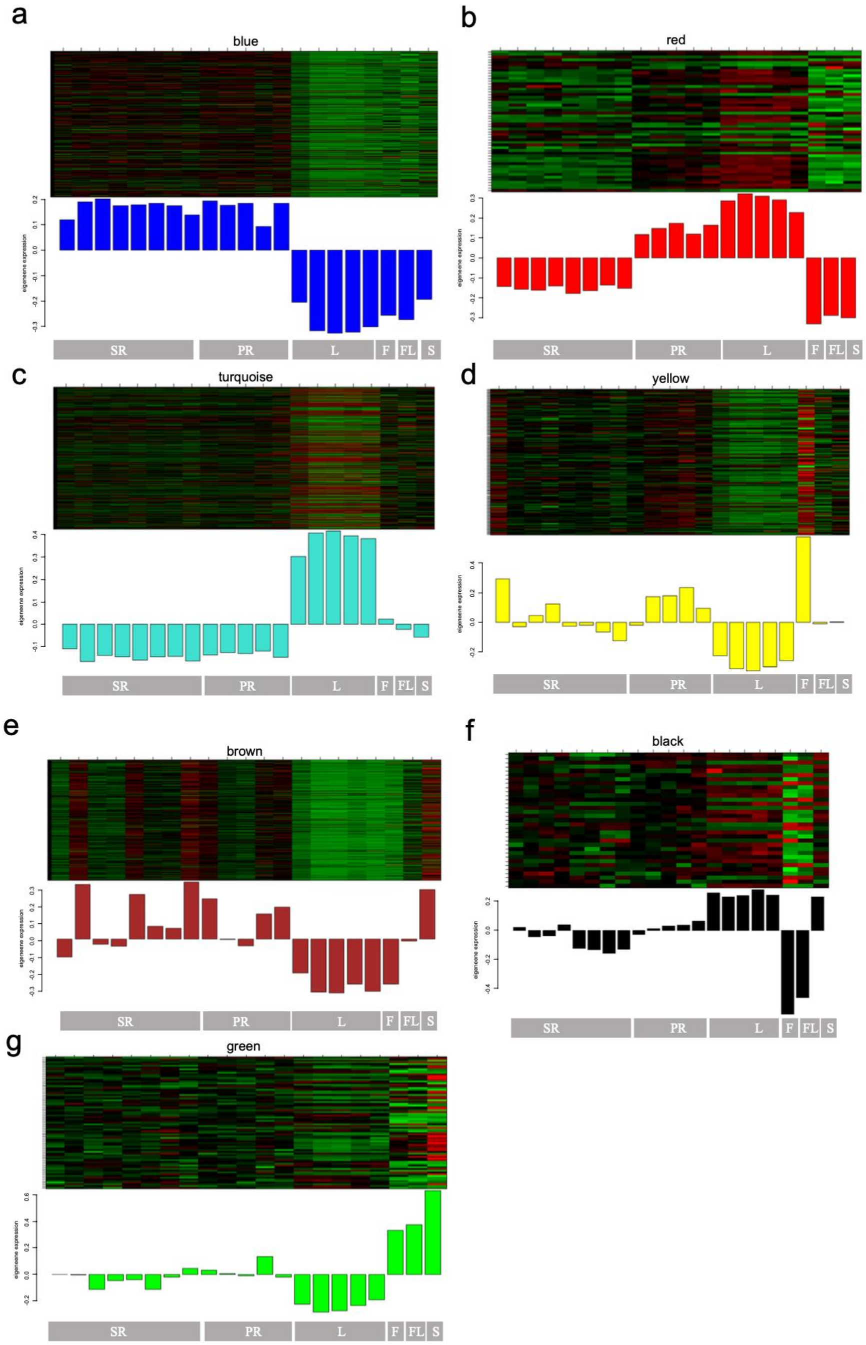


**Supplementary Fig. 42. Gene expression pattern of genes in the seven modules in the six different tissues if *A. belladonna*.** Clustering heatmap and bar plot represent expression levels of genes in blue module (**a**), red module (**b**), turquoise module (**c**), yellow module (**d**), brown module (**e**), black module (**f**) and green module (**g**). In the heatmaps, the colors range from green to red, indicating low to high expression levels, respectively. (SR: secondary roots; PR: primary roots; L: leaf; F: fruit; FL: flower; S: stem). The IDs and annotations of genes in each module were provided as Supplementary Data 3-9.

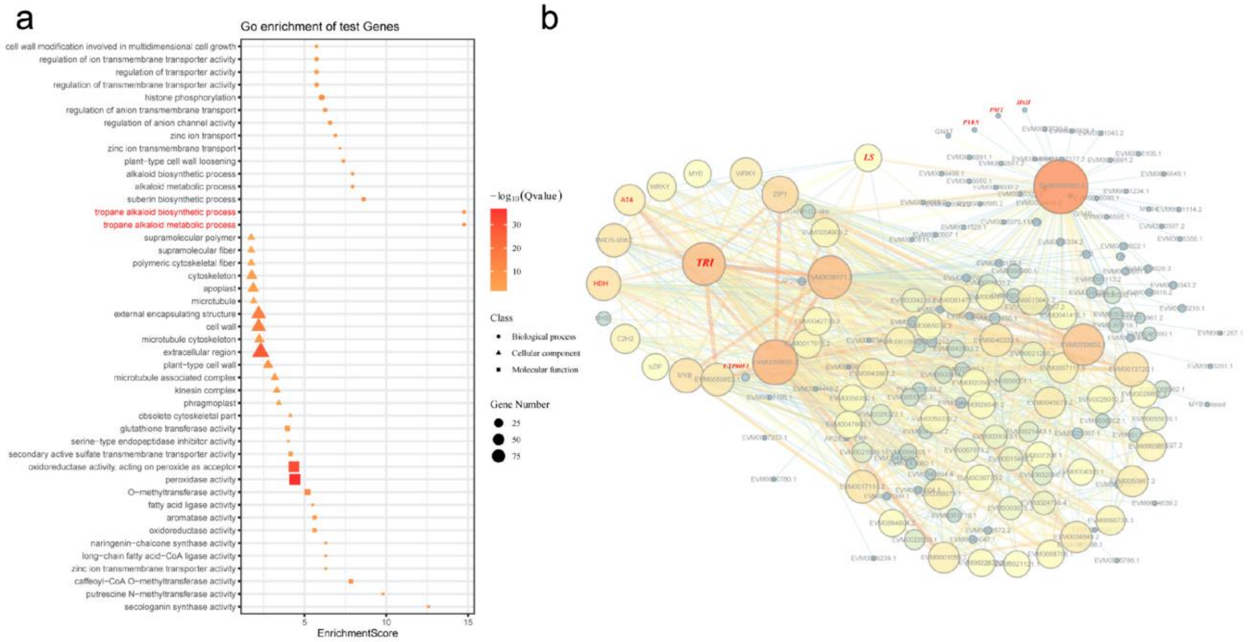


**Supplementary Fig. 43. Genes co-expressed with mTAs pathway genes in the 'blue' module.** **a** The GO enrichment analysis of genes in the WGCNA "blue" module; The GO terms of "tropane alkaloid biosynthetic process" and "tropane alkaloid metabolic process" are labelled by red. **b** The WGCNA "blue" module represented by a node and edge graph using Cytoscape, identified TA biosynthetic genes were labelled by red. All the gene IDs and annotations in this module were provided as Supplementary Data 3-9.

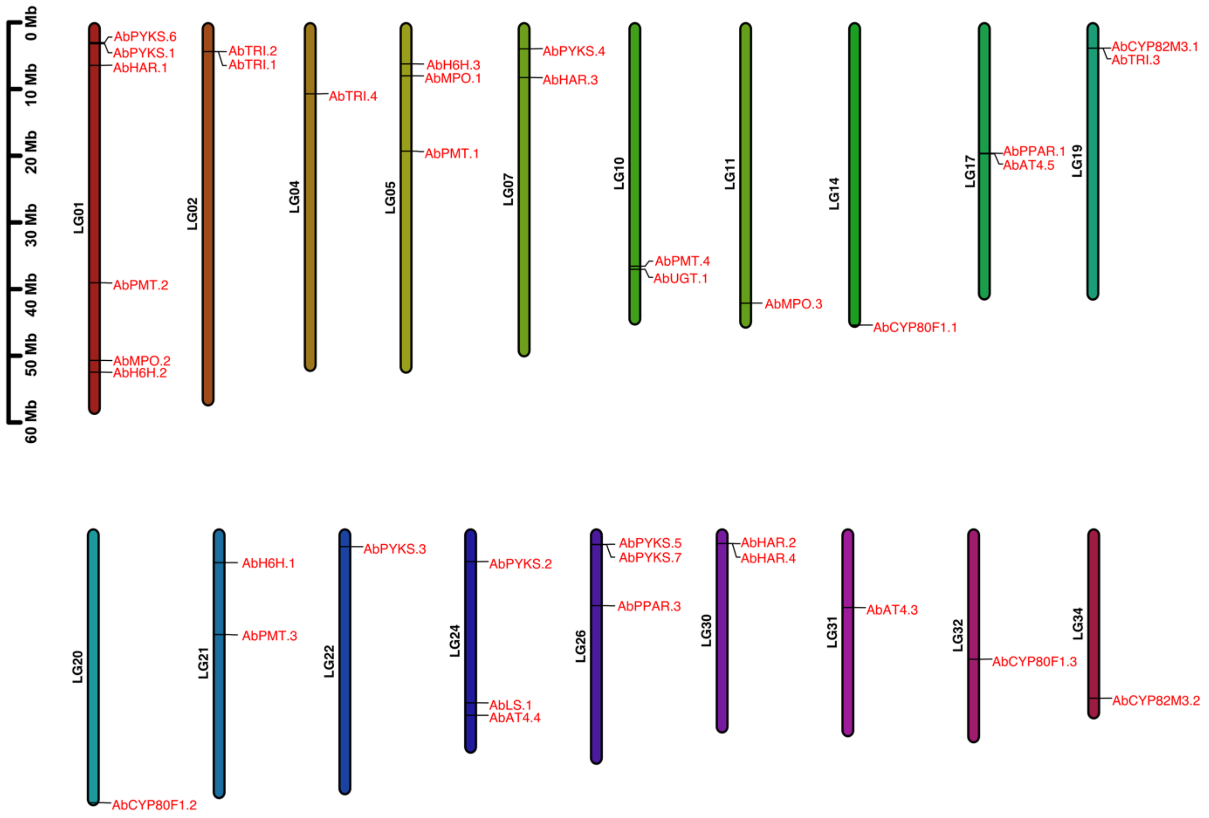


**Supplementary Fig. 44. The positions of mTAs biosynthetic genes on the chromosomes of *A. belladonna*.** The corresponding IDs and chromosomal positions of mTAs biosynthetic genes were provided in Supplementary Table 23.

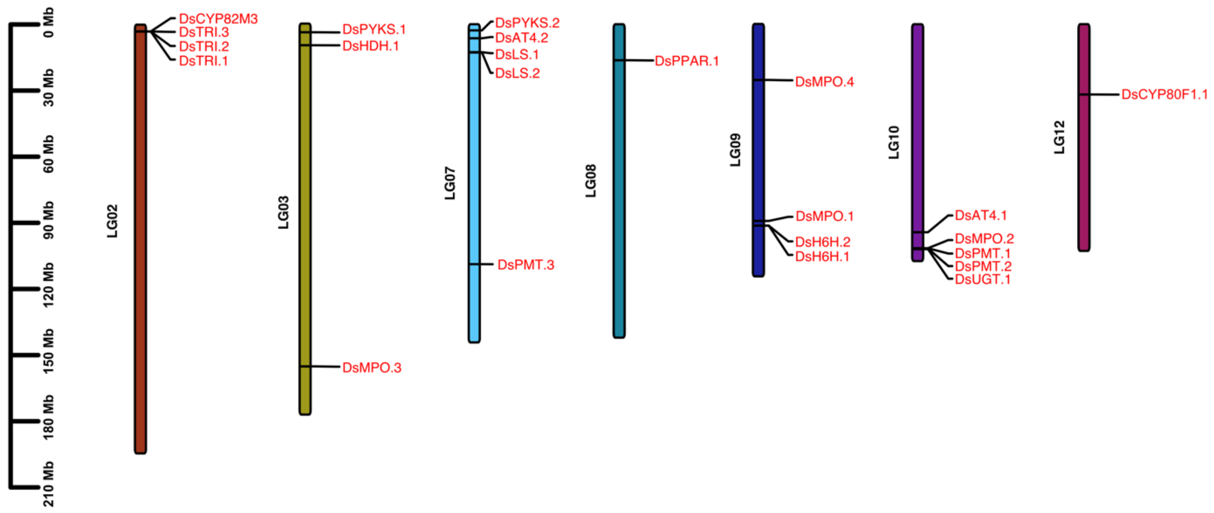


**Supplementary Fig. 45. The positions of mTAs biosynthetic genes on the chromosomes of *D. stramonium*.** The corresponding IDs and chromosomal positions of mTAs biosynthetic genes were provided in Supplementary Table 24.

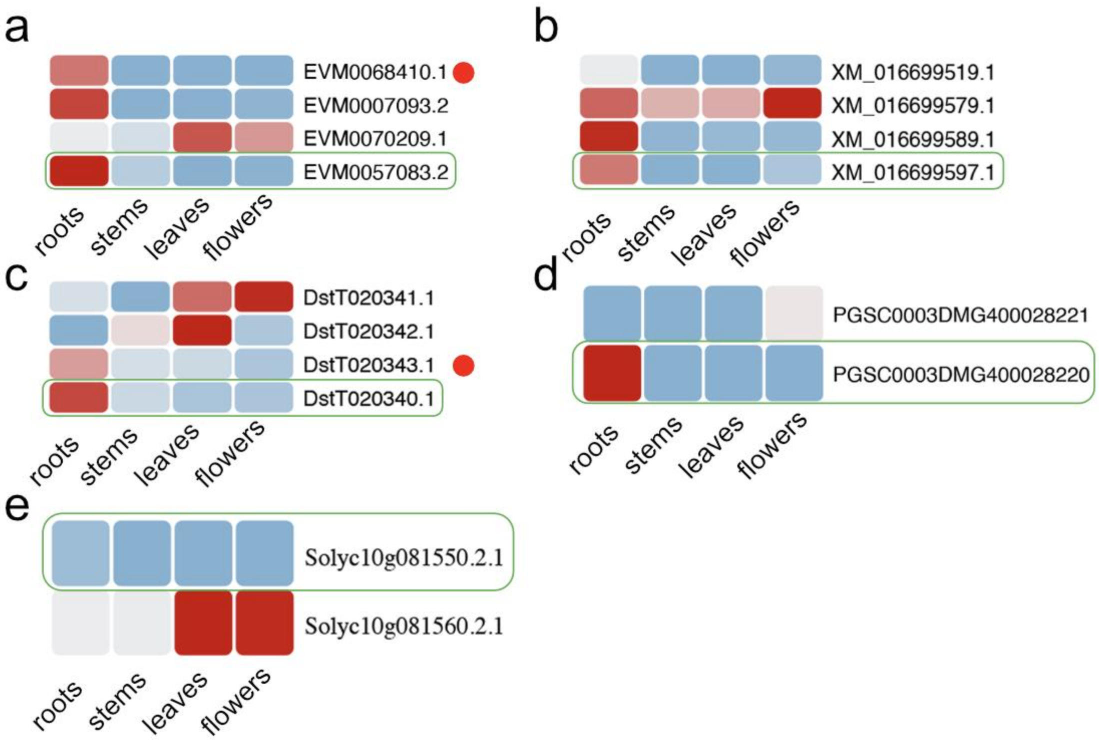


**Supplementary Fig. 46. Relative expression levels of *CYP82M3* and *TRI* in roots, stems, leaves, and flowers of *A. belladonna*, *C. annuum*, *D. stramonium*, *S. tuberosum* and *S. lycopersicum*.** **a** The relative expression levels of *CYP82M3* and *TRI* in tissues of *A. belladonna*. **b** The relative expression levels of *CYP82M3* and *TRI* in tissues of *C. annuum*. **c** The relative expression levels of *CYP82M3* and *TRI* in tissues of *D. stramonium*. **d** The relative expression levels of *CYP82M3* and *TRI* in tissues of *S. tuberosum*. **e** The relative expression levels of *CYP82M3* and *TRI* in tissues of *S. lycopersicum*. The row in the green box represents the expression profiles of *CYP82M3*, and the red dot represents the functional verification *TRI* gene. The raw data used here was downloaded from NCBI SRA database with accession number SRP019256 for *C. annuum* [3]., SRA030516 for *S. tuberosum* [4], and SRP229637 for *S. lycopersicum* [5].All the transcript per million values (TPM) were generated by HISAT2 and StringTie as the protocol in the manuscript. Source data are provided as a Source Data file.

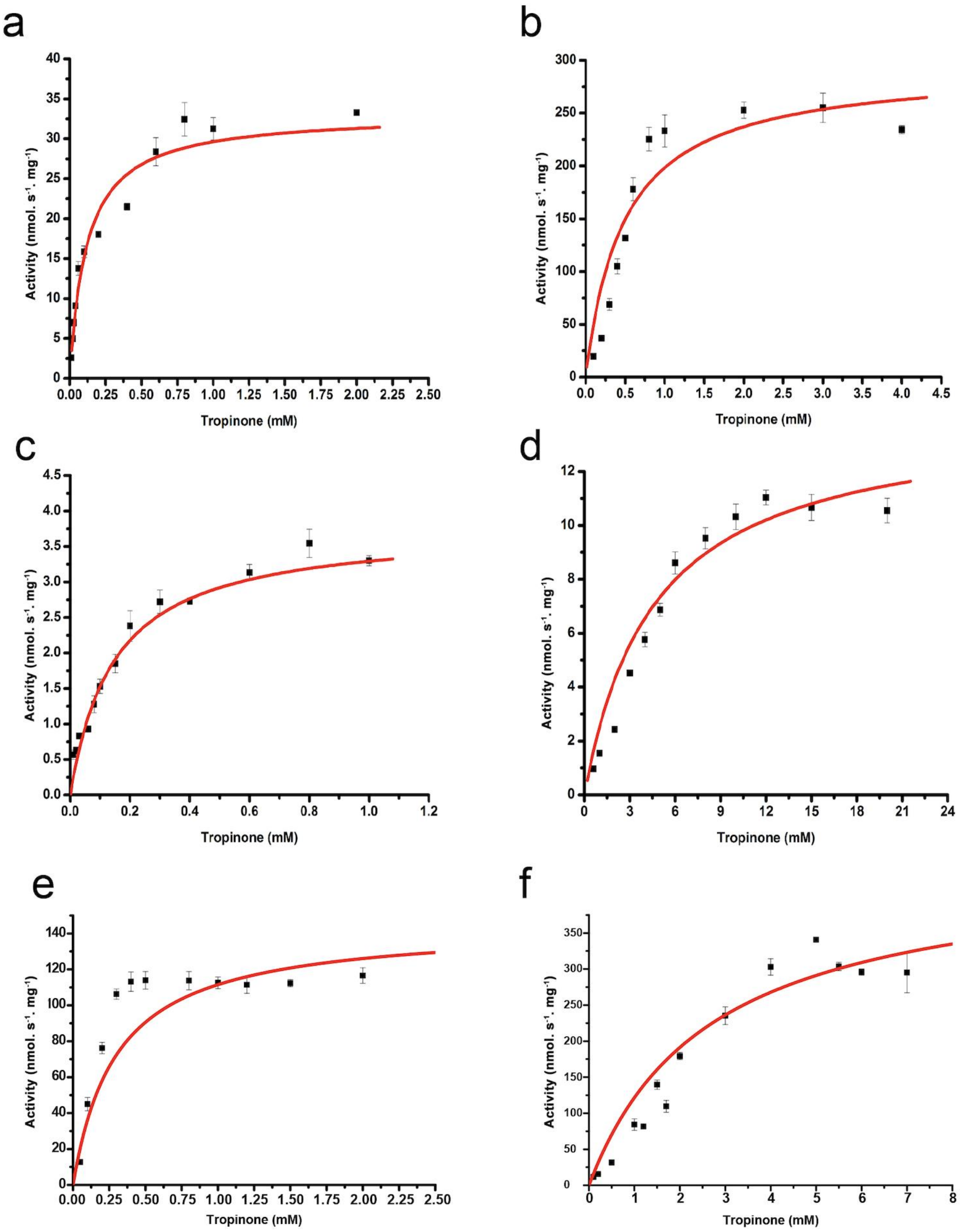


**Supplementary Fig. 47. Michaelis–Menten curves for the NADPH-dependent reduction reaction of tropinone of TRIs.** The Michaelis–Menten curves of AbTRI (**a**), DsTRI (**b**), CaTRI (**c**), SlTRI (**d**), SlTRI-L159V (**e**), StTRI-L159V (**f**). The buffer for the reduction reaction was potassium phosphate (0.1 M, pH 6.4). The data are shown as means ± s.d. (n = 3 biologically independent samples). Source data are provided as a Source Data file.

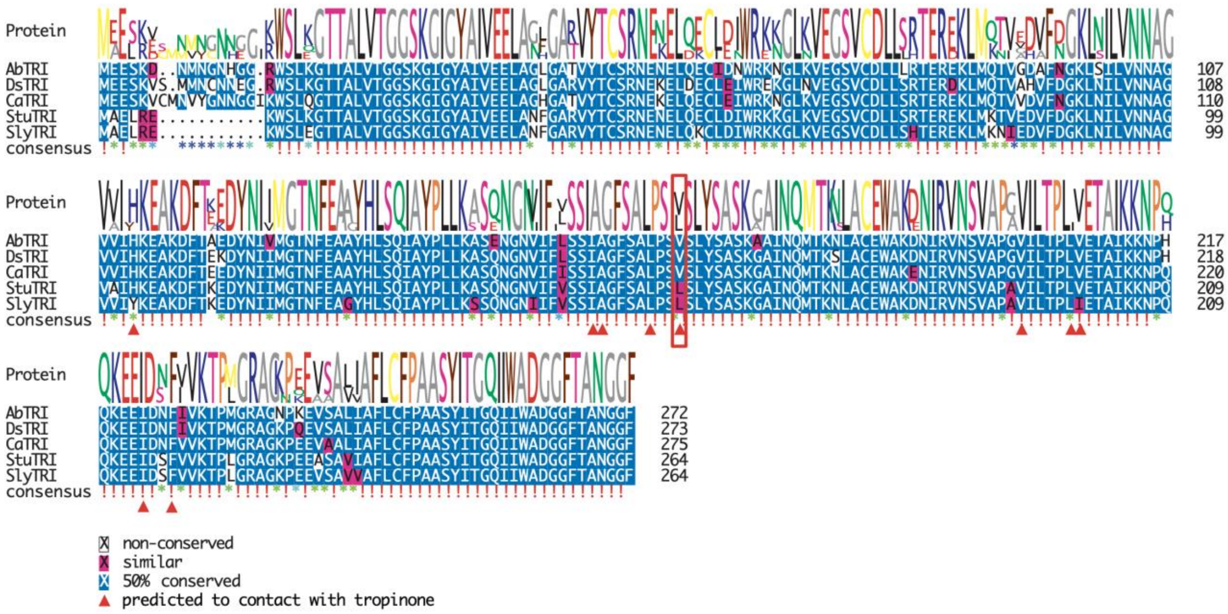


**Supplementary Fig. 48. The alignment of TRI amino acid sequence from *A. belladonna* (AbTRI), *C. annuum* (CaTRI), *D. stramonium* (DsTRI), *S. lycopersicum* (SlTRI) and *S. tuberosum* (StTRI)**. Amino acids predicted to be in contact with the tropinone substrate were indicted by red arrowheads, among which the common substitutions of SlTRI and StTRI were indicated by red box. The alignment was performed in R using msa package[6].

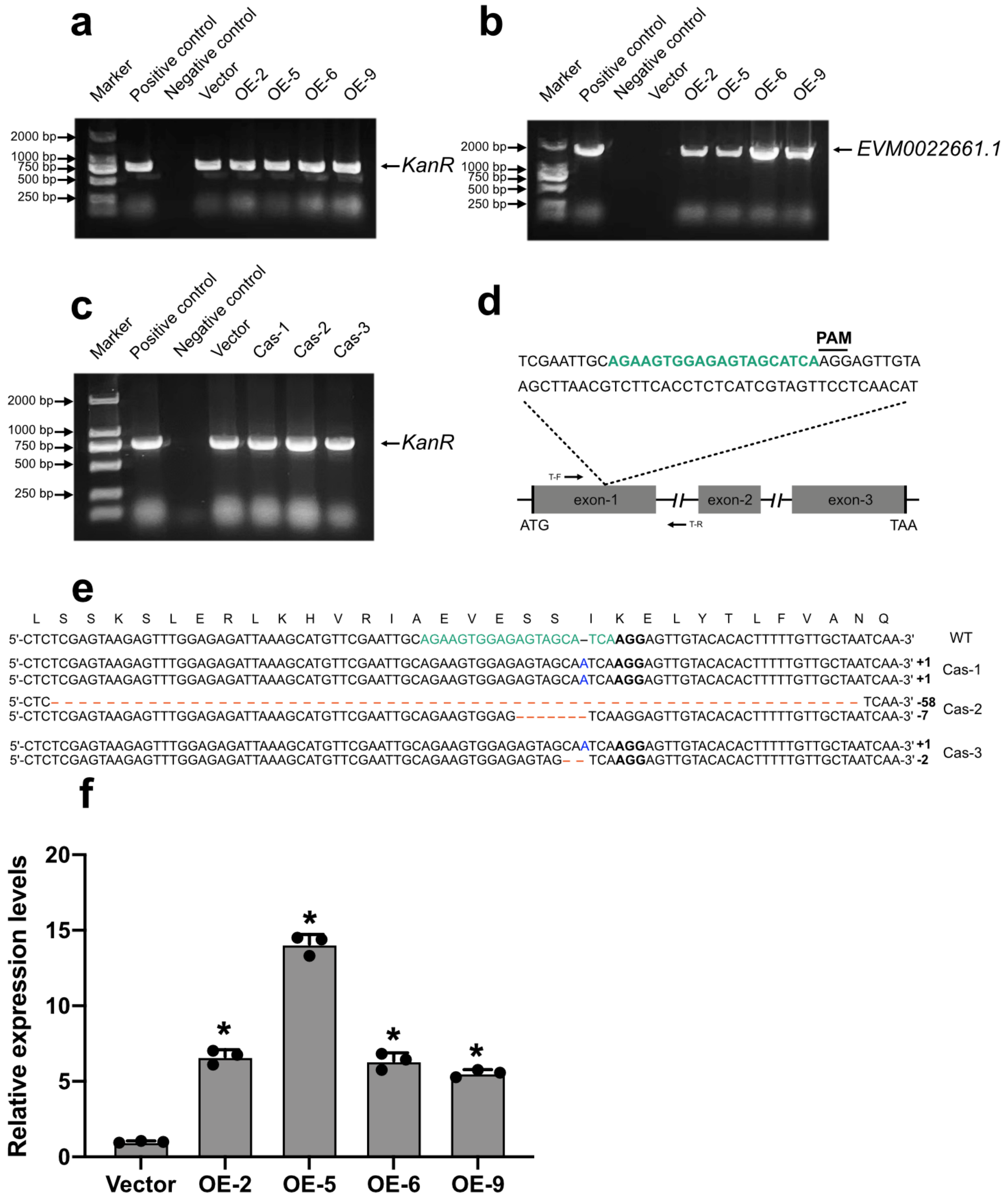


**Supplementary Fig. 49. The establishment of transgenic hairy roots of *A. belladonna* to validate the function of EVM0022661.1. a** Detecting the kanamycin resistant gene (*KanR*) in EVM0022661.1 overexpressed hairy roots of *A. belladonna* by genomic DNA PCR. This experiment was repeated independently three times with similar results. The unit for molecular

weight marker is base pair (bp) **b** Detecting the *EVM0022661.1* in EVM0022661.1 overexpressed hairy roots of *A. belladonna* by genomic DNA PCR. For **a** and **b**, Positive control: plasmid used for overexpression of *EVM0022661.1*; Negative control: Sterilized water was used as template instead of plasmid or genomic DNA. Vector: genomic DNA from hairy roots of *A. belladonna* which transformed with empty vector pBI121. This experiment was repeated independently three times with similar results. The unit for molecular weight marker is base pair (bp). **c** Detecting the *KanR* gene in *EVM0022661.1* edited hairy roots of *A. belladonna* by genomic DNA PCR. Positive control: plasmid used for editing *EVM0022661.1*; Negative control: Sterilized water was used as template instead of plasmid or genomic DNA. Vector: genomic DNA from hairy roots of *A. belladonna* which transformed with empty vector p1300-Cas9N[7]. This experiment was repeated independently three times with similar results. The unit for molecular weight marker is base pair (bp). **d** The structure of *EVM0022661.1* gene. Exons are indicated in grey boxes; sgRNAs sequence was showed by green letters. The Protospacer Adjacent Motif (PAM) was highlighted with a black line. Primers used for genotyping and sequencing are noted with black arrows. **e** Genotyping of gene editing events by Sanger Sequencing. The DNA sequence of each allele was aligned to wild type (WT) allele and deletions are shown with red hyphens and insertions marked with blue color. PAM was shown in bold. **f** The relative expression levels of *EVM0022661.1* in its overexpressed lines. *AbPGK* was used as inter a reference gene. The data are presented as means values +/- s.d. (n = 3 biologically independent samples). * represents significant difference from control line (Vector) analyzed by two-sided Student's *t*-test at the level of $P < 0.01$. *$P = 0.0000$ (OE-2), **$P = 0.0000$ (OE-5), **$P = 0.0001$ (OE-6), *$P = 0.0000$ (OE-9). Source data are provided as a Source Data file.

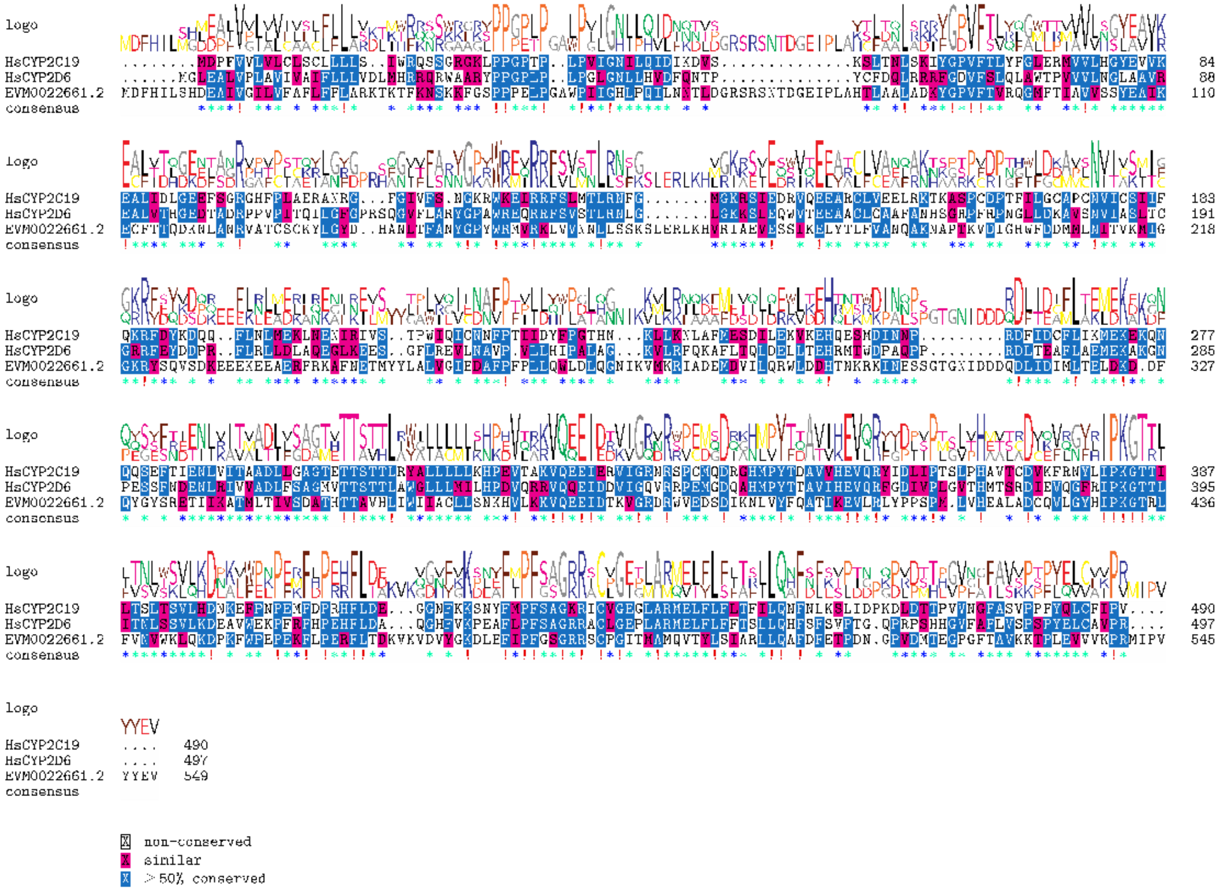


**Supplementary Fig. 50. The alignment of HsCYP2C19, HsCYP2D6 and EVM0022661.2.** The amino acid sequence of HsCYP2C19 and HsCYP2D6 were downloaded from UniPort by accession P33261 and P10635, respectively. The alignment was performed in R using msa package[6].

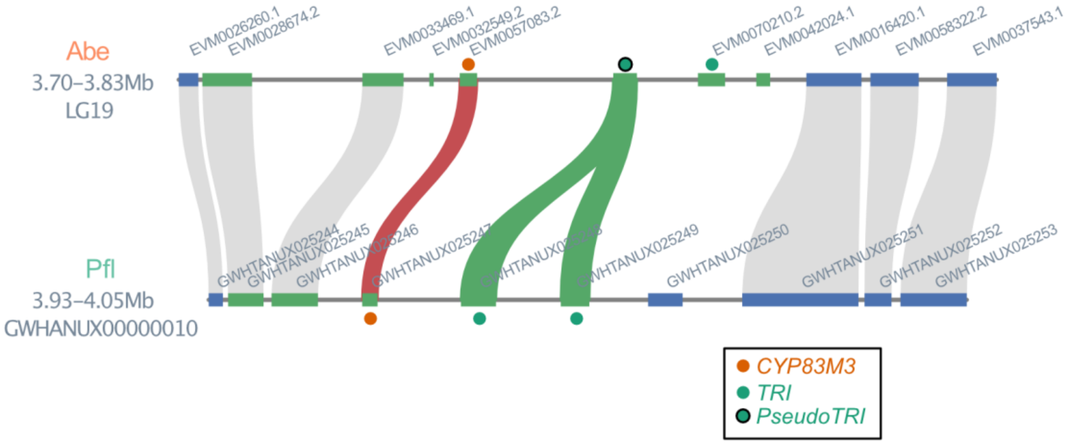


**Supplementary Fig. 51. Microsynteny analysis of *TRI* and *CYP82M3* genes between *A. belladonna* (Abe) and *P. floridana* (Pfl).** The syntenic *CYP82M3* genes were highlighted with red and the syntenic *TRI* genes were highlighted with green. The genomic region and chromosome ID were placed under the abbreviation of species names.

**Supplementary Table 1. Total Sequencing Data for *A. belladonna* and *D. stramonium*.**

| Species | Type | Platform | Library type | Reads number | Data size (Gb) | Mean read length (bp) | Read N50 (bp) |
|---|---|---|---|---|---|---|---|
| *A. belladonna* | Illumina short reads | HiSeq X (clean)[1] | Paired | 721,213,404 | 53.88 | 150 | - |
| | ONT long reads | Nanopore (raw)[1] | Single | - | 181.77 | - | - |
| | | Nanopore (clean) | Single | 9,121,916 | 170.64 | 18,706 | 20,732 |
| | Hi-C reads | HiSeq X (clean) | Paired | 815,475,452 | 244.16 | 150 | - |
| *D. stramonium* | Illumina short reads | HiSeq X (raw) | Paired | 993,083,816 | 148.96 | 150 | - |
| | | HiSeq X (clean) | Paired | 721,213,404 | 145.27 | 150 | - |
| | PacBio long reads | PicBio(clean) | Single | | 828.66 | | |
| | HiFi | | | 1,315 | 52.47 | | 105,166,977 |
| | Hi-C reads | HiSeq X (clean) | | 2,167,631,806 | 320.93 | 150 | - |

[1]The clean data means the raw data after quality control. For HiSeq reads, we used fastp software with those commands: -q 20 -5 -3; for long reads, we used NextDenovo to perform the error correction and obtain the final consensus sequences with the default parameters.

**Supplementary Table 2. Summary of *A. belladonna* and *D. stramonium* contig leveled assembly.**

| | ***A. belladonna*** | | ***D. stramonium*** | |
|---|---|---|---|---|
| | **Size (bp)** | **Number** | **Size (bp)** | **Number** |
| N90 | 632,303 | 282 | 13,621,149 | 25 |
| N50 | 3,033,052 | 49 | 105,166,977 | 7 |
| Longest | 15,204,373 | - | 220,483,397 | - |
| Total Contigs | 1,595,075,802 | 1,753 | 1,844,831,445 | 1,298 |
| Short reads mapping rate | 99.56% | | 99.88% | |
| Short reads mapping coverage | 99.63% | | 99.94% | |

**Supplementary Table 3. Summary of *A. belladonna* chromosome level assembly[1].**

| Chromosome ID | Chromosome length (bp)[2] | Contig number |
|---|---|---|
| LG01 | 62,395,467 | 72 |
| LG02 | 59,507,079 | 47 |
| LG03 | 58,036,661 | 43 |
| LG04 | 56,934,839 | 82 |
| LG05 | 55,684,019 | 66 |
| LG06 | 54,894,198 | 41 |
| LG07 | 53,385,413 | 62 |
| LG08 | 50,506,356 | 45 |
| LG09 | 50,466,939 | 33 |
| LG10 | 50,122,489 | 65 |
| LG11 | 53,097,641 | 61 |
| LG12 | 49,824,043 | 45 |
| LG13 | 49,401,233 | 50 |
| LG14 | 48,641,380 | 50 |
| LG15 | 45,352,135 | 37 |
| LG16 | 45,222,944 | 39 |
| LG17 | 44,725,625 | 52 |
| LG18 | 45,055,520 | 49 |
| LG19 | 43,773,306 | 38 |
| LG20 | 43,206,624 | 41 |
| LG21 | 43,121,716 | 55 |
| LG22 | 42,483,668 | 47 |
| LG23 | 39,216,978 | 48 |
| LG24 | 36,786,454 | 57 |
| LG25 | 37,472,170 | 30 |
| LG26 | 36,826,767 | 38 |
| LG27 | 35,326,183 | 37 |
| LG28 | 35,136,437 | 38 |
| LG29 | 34,953,490 | 43 |
| LG30 | 34,387,992 | 55 |
| LG31 | 33,975,835 | 34 |
| LG32 | 33,856,500 | 31 |
| LG33 | 33,556,702 | 31 |
| LG34 | 30,880,715 | 43 |
| LG35 | 30,649,314 | 37 |
| LG36 | 28,304,355 | 39 |
| Total | 1,587,169,187 | 1681 |

[1]Sequence number and length was calculated with all clustered sequences > 1 Kb.

[2]Sequence length were calculated by seqkit (v 0.11.0)

**Supplementary Table 4. Summary of *D. stramonium* chromosome leveled assembly[1].**

| Chromosome ID | Chromosome length (bp)[2] | Contig number |
|---|---|---|
| LG01 | 236,938,339 | 6 |
| LG02 | 194,861,799 | 5 |
| LG03 | 177,732,037 | 8 |
| LG04 | 168,010,536 | 9 |
| LG05 | 163,681,163 | 8 |
| LG06 | 163,060,219 | 5 |
| LG07 | 144,717,188 | 7 |
| LG08 | 142,548,149 | 14 |
| LG09 | 114,882,833 | 4 |
| LG10 | 107,833,081 | 7 |
| LG11 | 100,158,972 | 4 |
| LG12 | 82,813,623 | 8 |
| Total | 1,797,237,939 | 85 |

[1]Sequence number and length was calculated with all clustered sequences > 1 Kb.

[2]Sequence length were calculated by seqkit[8]

**Supplementary Table 5. Prediction of protein-coding genes in the *Atropa belladonna* genome.**

| Method | Software | Species[1] | Total genes predicted | Average gene length (bp) | Average CDS length (bp) | Average exon number per gene | Average exon length (bp) | Average intron length (bp) |
|---|---|---|---|---|---|---|---|---|
| *De novo* | Augustus | - | 55,136 | 5,985.75 | 1,446.67 | 5.91 | 244.64 | 923.82 |
| | Genscan | - | 60,109 | 14,486.71 | 1,203.35 | 6.09 | 197.74 | 2612 |
| | Glimmerhmm | - | 123,788 | 11,805.47 | 767.41 | 4.64 | 165.34 | 3031.17 |
| Homolog | GeMoMa | *S. tuberosum* | 69,487 | 3,245.31 | 952.94 | 4.3 | 221.55 | 694.41 |
| | | *S. lycopersicum* | 60,646 | 4,508.61 | 1,200.29 | 5.62 | 213.66 | 716.41 |
| | | *C. annuum* | 64,245 | 4,217.97 | 1,153.48 | 5.37 | 214.99 | 702.01 |
| | | *N. tabacum* | 58,522 | 4,022.16 | 996.27 | 4.93 | 202.05 | 769.78 |
| | | *S. pennellii* | 58,390 | 4,603.42 | 1,229.16 | 5.72 | 214.66 | 713.98 |
| | | *A. thaliana* | 49,375 | 4,663.51 | 1,224.28 | 5.81 | 210.72 | 715.01 |
| | | *S. melongena* | 84,085 | 3,855.54 | 945.13 | 4.84 | 195.09 | 757.03 |
| RNA-seq | PASA | | 109,613 | 2,975.71 | 1,056.45 | 3.03 | 348.35 | 944.17 |
| Integration | EVM | | 70,209 | 4,801.59 | 1,155.56 | 5.32 | 288.83 | 756.68 |

[1]The protein sequences of species were downloaded from https://phytozome.jgi.doe.gov/pz/portal.html

**Supplementary Table 6. Prediction of protein coding genes in *Datura stramonium* genome.**

| Method | Software | Species[1] | Total genes predicted | Average gene length (bp) | Average CDS length (bp) | Average exon number per gene | Average exon length (bp) | Average intron length (bp) |
|---|---|---|---|---|---|---|---|---|
| *De novo* | Augustus | - | 33,310 | 3,896.97 | 1,096.35 | 4.84 | 226.64 | 426.09 |
| | Genscan | - | 32,418 | 31,598.31 | 1,144.71 | 5.72 | 200.07 | 6,449.88 |
| | Glimmerhmm | - | 33,447 | 1,984.55 | 839.59 | 3.3 | 254.47 | 497.85 |
| Homolog | GeMoMa | *S. tuberosum* | 33,322 | 3,994.70 | 1,230.43 | 4.77 | 258.00. | 733.4 |
| | | *S. lycopersicum* | 31,827 | 4,141.02 | 1,238.14 | 4.92 | 251.41 | 739.63 |
| | | *C. annuum* | 39,172 | 3,547.75 | 1,106.19 | 4.38 | 252.34 | 721.58 |
| | | *N. tabacum* | 45,037 | 3,317.43 | 1,106.89 | 4.19 | 264.42 | 693.82 |
| | | *S. pennellii* | 31,328 | 4,140.61 | 1,241.26 | 4.87 | 255 | 749.64 |
| | | *A. thaliana* | 24,021 | 4,530.75 | 1,253.64 | 5.31 | 236.01 | 760.3 |
| | | *S. melongena* | 36,022 | 3,329.72 | 1,003.87 | 4.06 | 247.45 | 760.86 |
| RNA-seq | PASA | | 98,258 | 3,431.51 | 1,568.81 | 3.78 | 319.69 | 807.05 |
| Integration | EVM | | 32,037 | 3,913.23 | 1,127.27 | 4.79 | 235.26 | 734.77 |

[1]The protein sequences of species were downloaded from https://phytozome.jgi.doe.gov/pz/portal.html

**Supplementary Table 7. Assessing *Atropa belladonna* genome and annotation completeness with Benchmarking Universal Single-Copy Orthologs (BUSCO) analysis[1].**

| | ***Atropa belladonna* (Genome)** | | ***Atropa belladonna* (Predicted CDS)** | |
|---|---|---|---|---|
| **BUSCO notation** | **Number** | **Percentage (%)** | **Number** | **Percentage (%)** |
| Complete BUSCOs | 1595 | 98.9 | 1584 | 98.1 |
| Complete and single-copy BUSCOs | 598 | 37.1 | 691 | 42.8 |
| Complete and duplicated BUSCOSs | 997 | 61.8 | 893 | 55.3 |
| Fragemented BUSCOs | 10 | 0.6 | 14 | 0.9 |
| Missing BUSCOs | 9 | 0.5 | 16 | 1 |
| Total | 1614 | 100 | 1614 | 100 |

[1]Analyzed by BUSCO v 5.3.2 set for embryophyta_odb10.

**Supplementary Table 8. Assessing *Datura stramonium* genome and annotation completeness with Benchmarking Universal Single-Copy Orthologs (BUSCO) analysis[1].**

| | *Datura stramonium* (Genome) | | *Datura stramonium* (Predicted CDS) | |
|---|---|---|---|---|
| **BUSCO notation** | **Number** | **Percentage (%)** | **Number** | **Percentage (%)** |
| Complete BUSCOs | 1596 | 98.9 | 1522 | 94.3 |
| Complete and single-copy BUSCOs | 1582 | 98 | 1499 | 92.9 |
| Complete and duplicated BUSCOSs | 14 | 0.9 | 23 | 1.4 |
| Fragmented BUSCOs | 8 | 0.5 | 60 | 3.7 |
| Missing BUSCOs | 10 | 0.6 | 32 | 2 |
| Total | 1614 | 100 | 1614 | 100 |

[1]Analyzed by BUSCO v 5.3.2 set for embryophyta_odb10.

**Supplementary Table 9. Comparison of gene space of the *A. belladonna* and *D. stramonium* with other genomes.**

| Species | Gene predicted | Average gene length_(bp) | Average CDS length (bp) | Average exons per gene | Average exon length (bp) | Average intron length (bp) |
|---|---|---|---|---|---|---|
| *Atropa belladonna* | 70,209 | 4,835.40 | 1,155.94 | 5.12 | 225.71 | 740.07 |
| *Datura stramonium* | 32,037 | 3913.06 | 1,127.27 | 4.79 | 235.6 | 734.78 |
| *Solanum lycopersicum* | 35,768 | 4006.57 | 1,027.10 | 4.67 | 218.96 | 596.33 |
| *Capsicum annuum* | 45,410 | 7,842.20 | 1,271.90 | 5.58 | 227.5 | 1126.15 |
| *Nicotiana tabacum* | 35,519 | 4,780.75 | 1,067.46 | 4.86 | 227.91 | 947.92 |
| *Solanum melongena* | 36,568 | 4,584.49 | 990.7 | 4.48 | 286.41 | 945.55 |
| *Arabidopsis thaliana* | 28,496 | 2344.06 | 1,230.60 | 5.89 | 261.35 | 163.48 |
| *Solanum tuberosum* | 56,215 | 2424.98 | 905.3 | 3.6 | 250.39 | 583.18 |

**Supplementary Table 10. Functional annotation of the predicted genes for *A. belladonna* and *D. stramonium*.**

| | | *Atropa belladonna* | | *Datura stramonium* | |
|---|---|---|---|---|---|
| | **Database** | **Number** | **Percentage (%)** | **Number** | **Percentage (%)** |
| Total | | 67,551 | 96.22 | 28,431 | 88.74 |
| | SwissProt | 50,584 | 72.05 | 22,048 | 68.82 |
| | KOG | 36,833 | 52.46 | 12,356 | 38.57 |
| Annotated | GO | 32,371 | 46.11 | 18,274 | 57.04 |
| | NR | 67,012 | 95.45 | 28,293 | 88.31 |
| | TrEMBL | 31,895 | 45.43 | 28,312 | 88.37 |
| | KEGG | 24,519 | 34.92 | 9494 | 29.63 |
| Unannotated | | 2,657 | 3.78 | 3,606 | 11.26 |

**Supplementary Table 11. Statistics for non-coding RNA genes in the *A. belladonna* and *D. stramonium* genome.**

| | *A. belladonna* | | *D. stramonium* | |
|---|---|---|---|---|
| **Non-coding RNAs** | **Number** | **Average length (bp)** | **Number** | **Average length (bp)** |
| tRNA | 2,344 | 74.45 | 4,880 | 75.44 |
| rRNA | 2,837 | 242.27 | 13,055 | 995.76 |
| snRNA | 3,715 | 114.31 | 9,326 | 107.94 |
| miRNA | 437 | 126.79 | 173 | 124.55 |
| All | 9,333 | - | 27,434 | - |

**Supplementary Table 12. Prediction of repetitive elements in the assembled genomes of *A. belladonna* and *D. stramonium*.**

| | ***Atropa belladonna*** | | ***Datura stramonium*** | |
|---|---|---|---|---|
| | **Length(bp)** | **Rate of genome** | **Length(bp)** | **Rate of genome** |
| RepeatMasker | 346,143,435 | 21.70% | 580,620,867 | 31.47% |
| RepeatModeler | 939,398,608 | 58.89% | 1,494,009,102 | 80.98% |
| RepeatProteinMasker | 258,378,381 | 16.20% | 416,911,816 | 22.60% |
| TRF[1] | 53,164,701 | 3.33% | 66,677,113 | 3.61% |
| Total | 1,032,396,375 | 64.72% | 1,530,898,092 | 82.98% |

[1]TRF: Tandem Repeat Finder

**Supplementary Table 13. Prediction of transposable element in the assembled *A. belladonna* genome.**

| Type/Subfamily | | | Length (bp) | % of repeats | % of genome |
|---|---|---|---|---|---|
| **Class I: DNA Transposon** | DNA elements | | 98,956,649 | 9.585 | 6.204 |
| | | DNA_CMC-EnSpm | 10039199 | 0.972 | 0.629 |
| | | DNA_MuDR | 13479645 | 1.306 | 0.845 |
| | | DNA_PIF-Harbinger | 7743647 | 0.75 | 0.485 |
| | | DNA_TcMar-Stowaway | 17106322 | 1.657 | 1.072 |
| | | DNA_hAT-Ac | 26803433 | 2.596 | 1.68 |
| | | DNA_hAT-Tip100 | 1,158,720 | 0.076 | 0.063 |
| | | DNA_other | 21,722,247 | 2.104 | 1.362 |
| **Class II: Retrotransposon** | LINEs | | 55,536,894 | 5.379 | 3.482 |
| | | LINE_L1 | 29,367,106 | 2.845 | 1.841 |
| | | LINE_L2 | 319,987 | 0.031 | 0.02 |
| | | LINE_other | 26,136,404 | 2.532 | 1.638 |
| | LTR elements | | 667,410,300 | 64.647 | 41.84 |
| | | LTR_Copia | 233,012,977 | 22.57 | 14.608 |
| | | LTR_Gypsy | 401,842,956 | 38.923 | 25.192 |
| | | LTR_other | 36,299,048 | 3.516 | 2.276 |
| | | Low_complexity | 2,255,259 | 0.218 | 0.141 |
| | SINEs | | 10,324,828 | 1 | 0.647 |
| | | Satellite | 843277 | 0.082 | 0.053 |
| | | Simple_repeat | 60,899,888 | 5.899 | 3.818 |
| **Unclassified** | | Small_RNA | 701,081 | 0.068 | 0.044 |
| Low_complexity | | Unclassified_ARTEFACT | 706 | 0 | 0 |
| Simple_repeat | | Unclassified_RC | 6,330 | 0.001 | 0 |
| Satellite | | Unclassified_RC/Helitron | 1,118,101 | 0.108 | 0.07 |
| | | Unclassified_RC/Helitron-2 | 173 | 0 | 0 |
| | | Unclassified_Retroposon | 296,985 | 0.029 | 0.019 |
| | | Unclassified_Unknown | 200,973,763 | 19.467 | 12.599 |
| **Tandem repeats** | | | 53,164,701 | 5.15 | 3.33 |
| **Total content** | | | 1,032,396,375 | 100 | 64.72 |

**Supplementary Table 14. Prediction of transposable element in the assembled *Datura stramonium* genome.**

| Type/Subfamily | | | Length (bp) | % of repeats | % of genome |
|---|---|---|---|---|---|
| **Class I: DNA Transposon** | DNA elements | | 42,042,227 | 2.746 | 2.279 |
| | | DNA_CMC-EnSpm | 8,447,558 | 0.552 | 0.458 |
| | | DNA_MuDR | 3,958,650 | 0.259 | 0.215 |
| | | DNA_PIF-Harbinger | 4,342,788 | 0.284 | 0.235 |
| | | DNA_TcMar-Stowaway | 4,388,154 | 0.287 | 0.238 |
| | | DNA_hAT-Ac | 8,703,349 | 0.569 | 0.472 |
| | | DNA_hAT-Tip100 | 1,158,720 | 0.076 | 0.063 |
| | | DNA_other | 11,537,326 | 0.754 | 0.625 |
| **Class II: Retrotransposon** | LINEs | | 24,043,698 | 1.571 | 1.303 |
| | | LINE_L1 | 15,734,784 | 1.028 | 0.853 |
| | | LINE_L2 | 89,922 | 0.006 | 0.005 |
| | | LINE_other | 8,252,314 | 0.539 | 0.447 |
| | LTR elements | | 1,220,541,842 | 79.727 | 66.16 |
| | | LTR_Copia | 79,156,214 | 5.171 | 4.291 |
| | | LTR_Gypsy | 1,116,178,734 | 72.91 | 60.503 |
| | | LTR_other | 37,344,123 | 2.439 | 2.024 |
| | | Low_complexity | 2,006,028 | 0.131 | 0.109 |
| | SINEs | | 3,019,618 | 0.197 | 0.164 |
| | | Satellite | 1,734,778 | 0.113 | 0.094 |
| | | Simple_repeat | 89,289,347 | 5.832 | 4.84 |
| **Unclassified** | | Small_RNA | 22,221,670 | 1.452 | 1.205 |
| Low_complexity | | Unclassified_Other | 1,263,675 | 0.083 | 0.068 |
| Simple_repeat | | Unclassified_RC | 891 | 0 | 0 |
| Satellite | | Unclassified_RC/Helitron | 2,515,175 | 0.164 | 0.136 |
| | | Unclassified_RC/Helitron-2 | 216 | 0 | 0 |
| | | Unclassified_Retroposon | 1,498,057 | 0.098 | 0.081 |
| | | Unclassified_Unknown | 268,030,558 | 17.508 | 14.529 |
| **Tandem repeats** | | | 66,677,113 | 4.36 | 3.61 |
| **Total content** | | | 1,530,898,092 | 100 | 82.98 |

**Supplementary Table 15. The statistics of full-length transcriptome sequences of *Lycium chinense*.**

| Samples | Total cDNA number | cDNA Size | Subreads bases | Subreads reads number | Subreads N50 | Subreads length |
|---|---|---|---|---|---|---|
| *Lycium chinense* | 25,777 | 1-10k | 86,564,797,274 | 47,683,640 | 2,314 | 1,764 |

**Supplementary Table 16. Functional annotation of the predicted genes for *L. chinense*.**

| | Annotated number | 0<=length <1000 | 1000<=length <2000 | 2000<=length <3000 | 3000<=length <6000 | length>=6000 |
|---|---|---|---|---|---|---|
| GO | 19,603 | 3,087 | 8,408 | 5,163 | 2,863 | 82 |
| KEGG | 13,567 | 2,107 | 5,691 | 3,533 | 2,157 | 79 |
| KOG | 11,387 | 1,387 | 4,805 | 3,204 | 1,935 | 56 |
| NR | 24,761 | 3,881 | 10,113 | 6,598 | 4,037 | 132 |
| NT | 24,925 | 3,945 | 10,133 | 6,665 | 4,052 | 130 |
| Swissprot | 20,800 | 2,941 | 8,583 | 5,723 | 3,442 | 111 |
| All_Annotated | 25,263 | 4,084 | 10,231 | 6,711 | 4,105 | 132 |

**Supplementary Table 17. Assessing *Lycium chinense* transcriptome completeness with Benchmarking Universal Single-Copy Orthologs (BUSCO) analysis[1].**

| | ***Lycium chinense* (Predicted CDS)** | |
|---|---|---|
| **BUSCO notation** | **Number** | **Percentage (%)** |
| Complete BUSCOs | 1163 | 72 |
| Complete and single-copy BUSCOs | 917 | 56.8 |
| Complete and duplicated BUSCOSs | 246 | 15.2 |
| Fragmented BUSCOs | 48 | 3 |
| Missing BUSCOs | 403 | 25 |
| Total | 1614 | 100 |

[1]Analyzed by BUSCO v 5.3.2 set for embryophyta_odb10.

**Supplementary Table 18. Plant genomes for phylogenetic and comparative genomics analyses.**

| Species | Annotation version | URL or reference |
|---|---|---|
| *Nicotiana tabacum* | Edwards2017 | https://solgenomics.net/ |
| *Capsicum annuum* | v 1.0 | BioProject ID: PRJNA186921 on NCBI |
| *Solanum melongena* | v 1.0 | http://www.eggplant-hq.cn/ |
| *Mimulus guttatus* | v 1.0 | BioProject ID: PRJNA13880 on NCBI |
| *Coffea canephora* | v 1.0 | http://coffee-genome.org/ |
| *Petunia axillaris* | v1.6.2 | https://solgenomics.net |
| *Oryza sativa* | IRGSP-1.0 | www.phytozome.net |
| *Solanum lycopersicum* | Tomato iTAG v3.2 | http://solgenomics.net/ |
| *Solanum tuberosum* | Potato v 4.03 | https://solgenomics.net |
| *Vitis vinifera* | Vitis Genoscope v2 | http://www.plantgdb.org/VvGDB/ |

**Supplementary Table 19. Summary of the clustering of the gene family among the 12 genomes (including 8 Solanaceae species and 4 other angiosperms).**

| Species | Total genes | Genes in families | Unclustered genes | Families | Unique families | Genes per family | Maximum gene family size |
|---|---|---|---|---|---|---|---|
| *A. belladonna* | 70,210 | 43,921 | 26,287 | 23,879 | 1,172 | 1.84 | 36 |
| *C. annuum* | 30,124 | 24,404 | 5,720 | 19,649 | 473 | 1.24 | 56 |
| *C. canephora* | 25,571 | 19,748 | 5,823 | 15,599 | 754 | 1.27 | 39 |
| *D. stramonium* | 32,037 | 26,109 | 5,928 | 20,328 | 657 | 1.28 | 71 |
| *M. guttatus* | 26,688 | 22,202 | 4,486 | 15,085 | 873 | 1.47 | 86 |
| *N. tabacum* | 55,685 | 45,845 | 9,840 | 22,136 | 1,111 | 2.07 | 111 |
| *O. sativa* | 24,359 | 15,429 | 8,930 | 9,858 | 1,627 | 1.57 | 38 |
| *P. axillaris* | 32,928 | 25,257 | 7,671 | 19,650 | 612 | 1.29 | 62 |
| *S. lycopersicum* | 25,365 | 23,224 | 2,141 | 20,125 | 130 | 1.15 | 41 |
| *S. melongena* | 36,567 | 25,998 | 10,569 | 20,818 | 849 | 1.25 | 82 |
| *S. tuberosum* | 28,053 | 24,509 | 3,544 | 20,617 | 199 | 1.19 | 35 |
| *V. vinifera* | 25,015 | 21,706 | 3,309 | 15,355 | 676 | 1.41 | 54 |
| *All* | 412,600 | 318,352 | 94,248 | 37,515 | - | 8.49 | - |

**Supplementary Table 20. KEGG enrichment analysis of the expanded gene families in *A. belladonna*[1].**

| Map ID | Map title | # of enriched genes | # of genes in the background | *P*_value[2] | Adjusted *P*_value[3] |
|---|---|---|---|---|---|
| A09140 | Cellular Processes | 50 | 1070 | 0.00766 | 0.037001 |
| A09140 | Transport and catabolism | 50 | 1070 | 0.00766 | 0.037001 |
| A09180 | Brite Hierarchies | 583 | 16861 | 0.00657 | 0.036294 |
| A09140 | Phagosome | 16 | 243 | 0.00662 | 0.034899 |
| A09120 | Spliceosome | 30 | 564 | 0.00673 | 0.033923 |
| A09180 | Translation factors | 25 | 436 | 0.00514 | 0.029827 |
| A09150 | Plant-pathogen interaction | 32 | 576 | 0.00275 | 0.017739 |
| A09100 | Energy metabolism | 55 | 1140 | 0.00276 | 0.016873 |
| A09130 | MAPK signaling pathway - plant | 27 | 453 | 0.0022 | 0.015016 |
| A09140 | Endocytosis | 32 | 493 | 0.00021 | 0.00152 |
| A09130 | Environmental Information Processing | 70 | 1344 | 8.7E-05 | 0.000674 |
| A09130 | Plant hormone signal transduction | 50 | 850 | 5.2E-05 | 0.000429 |
| A09180 | Exosome | 81 | 1567 | 3.1E-05 | 0.000276 |
| A09120 | RNA transport | 34 | 484 | 2.9E-05 | 0.000276 |
| A09130 | Signal transduction | 69 | 1252 | 1.7E-05 | 0.00018 |
| A09120 | Protein processing in endoplasmic reticulum | 43 | 633 | 6E-06 | 0.0000697 |
| A09100 | Ether lipid metabolism | 13 | 69 | 3.2E-07 | 0.00000467 |
| A09100 | Oxidative phosphorylation | 37 | 450 | 3.3E-07 | 0.0000042 |
| A09180 | Cytoskeleton proteins | 38 | 450 | 1.2E-07 | 0.0000019 |
| A09180 | Transcription factors | 78 | 1214 | 1E-08 | 0.000000198 |
| A09180 | Protein families: genetic information processing | 484 | 11749 | 1.9E-13 | 4.37E-12 |
| A09120 | Translation | 190 | 2030 | 1E-16 | 1E-16 |
| A09120 | Ribosome | 138 | 923 | 1E-16 | 1E-16 |
| A09180 | Ribosome | 140 | 979 | 1E-16 | 1E-16 |
| A09120 | Genetic Information Processing | 269 | 4637 | 1E-16 | 1E-16 |

[1]Associated with Supplementary Fig. 6

[2]The *P*-value was estimated by two-sided hypergeometric test.

[3]The adjusted *P*-value was estimated by Benjaminiand Hochberg (BH) method.

**Supplementary Table 21. KEGG enrichment analysis of the expanded gene families in *D. stramonium*[1].**

| Map ID | Map title | # of enriched genes | # of genes in the background | *P*_value[2] | Adjusted *P*_value[3] |
|---|---|---|---|---|---|
| A09100 | Oxidative phosphorylation | 45 | 170 | 2.22E-16 | 1.31E-14 |
| A09100 | Energy metabolism | 45 | 382 | 4.44E-16 | 1.31E-14 |
| A09100 | Metabolism | 103 | 2745 | 7.77E-16 | 1.53E-14 |
| A09180 | Mitochondrial biogenesis | 29 | 363 | 2.87E-11 | 3.39E-10 |
| A09100 | Pentose and glucuronate interconversions | 18 | 129 | 2.66E-11 | 3.92E-10 |
| A09100 | Sesquiterpenoid and triterpenoid biosynthesis | 9 | 45 | 1.20E-07 | 1.18E-06 |
| A09100 | Flavonoid biosynthesis | 9 | 57 | 1.00E-06 | 8.45E-06 |
| A09180 | Prenyltransferases | 8 | 55 | 7.71E-06 | 5.68E-05 |
| A09100 | Metabolism of terpenoids and polyketides | 17 | 264 | 9.32E-06 | 6.11E-05 |
| A09100 | Zeatin biosynthesis | 8 | 60 | 1.50E-05 | 8.82E-05 |
| A09100 | Biosynthesis of unsaturated fatty acids | 5 | 25 | 8.75E-05 | 4.69E-04 |
| A09100 | Stilbenoid, diarylheptanoid and gingerol biosynthesis | 6 | 48 | 2.60E-04 | 0.001280461 |
| A09100 | Fatty acid biosynthesis | 5 | 55 | 0.003634 | 0.01531278 |
| A09100 | Fructose and mannose metabolism | 5 | 68 | 0.008956 | 0.033025171 |
| A09120 | Protein processing in endoplasmic reticulum | 10 | 221 | 0.008899 | 0.03500133 |
| A09100 | Biosynthesis of other secondary metabolites | 13 | 340 | 0.011903 | 0.041311675 |
| A09100 | Carbohydrate metabolism | 24 | 819 | 0.019289 | 0.056901116 |
| A09150 | Organismal Systems | 10 | 249 | 0.019213 | 0.062977249 |
| A09150 | Environmental adaptation | 10 | 249 | 0.019213 | 0.062977249 |
| A09180 | Lipid biosynthesis proteins | 5 | 93 | 0.030774 | 0.086459701 |

[1]Associated with Supplementary Fig. 6

[2]The *P*-value was estimated by two-sided hypergeometric test.

[3]The adjusted *P*-value was estimated by Benjaminiand Hochberg (BH) method.

**Supplementary Table 22. enzymatic kinetic of PPAR from *A. belladonna*, *D. stramonium*, *P. axillaris*, *C. annuum* and *S. lycopersicum*.**

| Enzymes | Species | $K_m$ (mM) | *Vmax* (nmol·s$^{-1}$·mg$^{-1}$) | $K_{cat}$ (s$^{-1}$) |
|---|---|---|---|---|
| AbPPAR | *A. belladonna* | 2.08469 ± 0.46131 | 0.23113 ± 0.01745 | 0.00980 ± 0.00074 |
| DsPPAR | *D. stramonium* | 0.54819 ± 0.06783 | 0.38352 ± 0.01877 | 0.01626 ± 0.00080 |
| CaPPAR | *C. annuum* | 3.01672 ± 0.48493 | 0.05862 ± 0.00578 | 0.00249 ± 0.00025 |
| SlPPAR | *S. lycopersicum* | 1.38010 ± 0.13005 | 0.79137 ± 0.05970 | 0.03355 ± 0.00253 |
| PaPPAR | *P. axillaris* | 2.64959 ± 0.37406 | 0.07847 ± 0.00870 | 0.00332 ± 0.00037 |

**Supplementary Table 23. The IDs and positions of mTAs biosynthetic genes on the chromosomes of *A. belladonna*.**

| Gene_name | Gene_ID | Chromosome | Start | End |
|---|---|---|---|---|
| AbPYKS.6 | EVM0002550.1 | LG01 | 2907005 | 2909807 |
| AbPYKS.1 | EVM0041167.2 | LG01 | 3093338 | 3098402 |
| AbPMT.2 | EVM0050126.2 | LG01 | 38944397 | 38948424 |
| AbMPO.2 | EVM0024232.2 | LG01 | 50584624 | 50588761 |
| AbH6H.2 | EVM0054624.2 | LG01 | 52354798 | 52360324 |
| AbHAR.1 | EVM0014492.2 | LG01 | 6333802 | 6338378 |
| AbTRI.2 | EVM0070209.1 | LG02 | 4263963 | 4267905 |
| AbTRI.1 | EVM0068410.1 | LG02 | 4269103 | 4272780 |
| AbTRI.4 | EVM0033773.2 | LG04 | 10598287 | 10605977 |
| AbPMT.1 | EVM0048770.2 | LG05 | 19204089 | 19208512 |
| AbH6H.3 | EVM0058768.1 | LG05 | 6115492 | 6121759 |
| AbMPO.1 | EVM0017027.2 | LG05 | 7897425 | 7905032 |
| AbPYKS.4 | EVM0042081.1 | LG07 | 3853171 | 3854887 |
| AbHAR.3 | EVM0005196.2 | LG07 | 8157154 | 8161449 |
| AbPMT.4 | EVM0001688.2 | LG10 | 36447907 | 36454327 |
| AbUGT.1 | EVM0039076.2 | LG10 | 36914763 | 36936802 |
| AbCYP80F1.1 | EVM0010440.2 | LG14 | 45277773 | 45280111 |
| AbPPAR.1 | EVM0020147.2 | LG17 | 19508844 | 19512115 |
| AbAT4.5 | EVM0046113.2 | LG17 | 19610548 | 19610940 |
| AbCYP82M3.1 | EVM0057083.2 | LG19 | 3746581 | 3749381 |
| AbTRI.3 | EVM0007093.2 | LG19 | 3771284 | 3775098 |
| AbCYP80F1.2 | EVM0054063.2 | LG20 | 40982085 | 40988094 |
| AbPMT.3 | EVM0056752.1 | LG21 | 15764770 | 15769397 |
| AbH6H.1 | EVM0035644.2 | LG21 | 4980656 | 4983978 |
| AbPYKS.3 | EVM0020110.2 | LG22 | 2579990 | 2581908 |
| AbSCPL.1 | EVM0063957.1 | LG24 | 26003758 | 26010341 |
| AbAT4.4 | EVM0068655.2 | LG24 | 27877573 | 27883353 |
| AbPYKS.2 | EVM0056449.2 | LG24 | 4819790 | 4822919 |
| AbPPAR.3 | EVM0006691.2 | LG26 | 11433082 | 11439135 |
| AbPYKS.5 | EVM0046717.2 | LG26 | 2254198 | 2256154 |
| AbPYKS.7 | EVM0054253.2 | LG26 | 2277661 | 2278521 |
| AbHAR.2 | EVM0023344.2 | LG30 | 2113711 | 2117010 |
| AbHAR.4 | EVM0068664.1 | LG30 | 2121209 | 2121910 |
| AbAT4.3 | EVM0058190.2 | LG31 | 11714225 | 11720048 |
| AbCYP80F1.3 | EVM0050976.2 | LG32 | 19471478 | 19473527 |
| AbCYP82M3.2 | EVM0005713.1 | LG34 | 25319287 | 25321750 |
| AbMPO.3 | EVM0068072.1 | LG11 | 42117601 | 42125714 |

**Supplementary Table 24. The IDs and positions of mTAs biosynthetic genes on the chromosomes of *D. stramonium*.**

| Gene_name | Gene_ID | Chromosome | Start | End |
|---|---|---|---|---|
| DsCYP82M3 | DstT020340.1 | LG2 | 3080365 | 3082629 |
| DsTRI.3 | DstT020341.1 | LG2 | 3096449 | 3098758 |
| DsTRI.2 | DstT020342.1 | LG2 | 3102977 | 3105550 |
| DsTRI.1 | DstT020343.1 | LG2 | 3109138 | 3113619 |
| DsMPO.3 | DstT005821.1 | LG3 | 155497551 | 155504981 |
| DsPYKS.1 | DstT006869.1 | LG3 | 3940161 | 3941142 |
| DsHDH.1 | DstT006643.1 | LG3 | 9829402 | 9833430 |
| DsPMT.3 | DstT004167.1 | LG7 | 108853807 | 108854870 |
| DsLS.1 | DstT004733.1 | LG7 | 12690926 | 12697497 |
| DsLS.2 | DstT004731.1 | LG7 | 12794533 | 12847935 |
| DsPYKS.2 | DstT027385.1 | LG7 | 2799186 | 2800143 |
| DsAT4.2 | DstT004990.1 | LG7 | 6365775 | 6370559 |
| DsPPAR | DstT013938.1 | LG8 | 16346905 | 16348919 |
| DsMPO.4 | DstT008907.1 | LG9 | 25411343 | 25417813 |
| DsMPO.1 | DstT009327.1 | LG9 | 89372023 | 89378652 |
| DsH6H.2 | DstT009494.1 | LG9 | 91495185 | 91498879 |
| DsH6H.1 | DstT009495.1 | LG9 | 91502455 | 91504801 |
| DsMPO.2 | DstT013422.1 | LG10 | 101602434 | 101607703 |
| DsPMT.1 | DstT013423.1 | LG10 | 101632464 | 101635075 |
| DsPMT.2 | DstT013425.1 | LG10 | 101693062 | 101696000 |
| DsUGT.1 | DstT013443.1 | LG10 | 102044005 | 102045463 |
| DsAT4.1 | DstT013249.1 | LG10 | 94355928 | 94364000 |
| DsCYP80F1.1 | DstT019520.1 | LG12 | 11279267 | 11281192 |

**Supplementary Table 25. RNA-seq datasets used in this study.**

| Species | Tissue | SRA | Using |
|---|---|---|---|
| *A. belladonna* | The leaves of mature *A. belladonna* | SRR21736115 | Genome survey |
| | The secondary roots of mature *A. belladonna* | SRR21397100 | Transcriptome |
| | The primary roots of mature *A. belladonna* | SRR21397101 | Transcriptome |
| | The leaves of mature *A. belladonna* | SRR21397102 | Transcriptome |
| | The primary roots of *A. belladonna* at development stages 5 | SRR16072209 | Transcriptome |
| | The primary roots of *A. belladonna* at development stages 4 | SRR16072210 | Transcriptome |
| | The primary roots of *A. belladonna* at development stages 3 | SRR16072211 | Transcriptome |
| | The primary roots of *A. belladonna* at development stages 2 | SRR16072212 | Transcriptome |
| | The primary roots of *A. belladonna* at development stages 1 | SRR16072213 | Transcriptome |
| | The young leaves of *A. belladonna* at development stages 5 | SRR16072214 | Transcriptome |
| | The young leaves of *A. belladonna* at development stages 4 | SRR16072215 | Transcriptome |
| | The young leaves of *A. belladonna* at development stages 3 | SRR16072217 | Transcriptome |
| | The young leaves of *A. belladonna* at development stages 2 | SRR16072228 | Transcriptome |
| | The young leaves of *A. belladonna* at development stages 1 | SRR16072229 | Transcriptome |
| | The steams of mature *A. belladonna* | SRR16072216 | Transcriptome |
| | The flowers of mature *A. belladonna* | SRR16072218 | Transcriptome |
| | The fruits of mature *A. belladonna* | SRR16072219 | Transcriptome |
| | The secondary roots of *A. belladonna* at development stages 8 | SRR16072220 | Transcriptome |
| | The secondary roots of *A. belladonna* at development stages 7 | SRR16072221 | Transcriptome |
| | The secondary roots of *A. belladonna* at development stages 6 | SRR16072222 | Transcriptome |
| | The secondary roots of *A. belladonna* at development stages 5 | SRR16072223 | Transcriptome |
| | The secondary roots of *A. belladonna* at development stages 4 | SRR16072224 | Transcriptome |
| | The secondary roots of *A. belladonna* at development stages 3 | SRR16072225 | Transcriptome |
| | The secondary roots of *A. belladonna* at development stages 2 | SRR16072226 | Transcriptome |
| | The secondary roots of *A. belladonna* at development stages 1 | SRR16072227 | Transcriptome |
| *D. stramonium* | The leaves of mature *D. stramonium* | SRR16068630 | Genome survey |
| | The leaves of mature *D. stramonium* | SRR16070409 | Transcriptome |
| | The secondary roots of mature *D. stramonium* | SRR16070407 | Transcriptome |
| | The primary roots of mature *D. stramonium* | SRR16070406 | Transcriptome |
| | The fruits of mature *D. stramonium* | SRR16070405 | Transcriptome |
| | The flowers of mature *D. stramonium* | SRR16070404 | Transcriptome |
| | The stems of mature *D. stramonium* | SRR16070408 | Transcriptome |
| *L. chinense* | The full-length transcriptome | SRR16248231 | full-length transcriptome |

**Supplementary Table 26. Primers for functional characterization of MPO.**

| Primer name | Purpose | Sequence (5' to 3') |
|---|---|---|
| qAbMPO1-F | Forward primer for quantification of *AbMPO1* | AGTTGTTCCAGATGTTCAG |
| qAbMPO1-R | Reverse primer for quantification of *AbMPO1* | CTCTTCTCTTCATTGCTTCTA |
| qAbMPO2-F | Forward primer for quantification of *AbMPO2* | CCTCCAATGGACGCTGCT |
| qAbMPO2-R | Reverse primer for quantification of *AbMPO2* | GATAAGAGGTTTTCCTAGACG |
| CaMV35S-F | Forward primer for genomic PCR detection | CGTAAGGGATGACGCACAATCCC |
| AbMPO1-R | Reverse primer for genomic PCR detection of *AbMPO1* | GTGGCGAGCGGCCCATATTAAT |
| AbMPO2-R | Reverse primer for genomic PCR detection of *AbMPO2* | CTGCTTTCTCCATTGAAACTC |
| Ri-AbMPO1-F1 | Forward primer for construction of RNAi interference plasmid of *AbMPO1* | TGGGTTCGAAATCGATAAGCTTGTGGCGAGCGGCCCATATTAAT |
| Ri-AbMPO1-R1 | Reverse primer for construction of RNAi interference plasmid of *AbMPO1* | CATTAAAGCAGGACTCTAGAGCTCGCCTTGGAGGAAGTCTAG |
| Ri-AbMPO1-F2 | Forward primer for construction of RNAi interference plasmid of *AbMPO1* | TCCTTACCAATTGGGGTACCGTGGCGAGCGGCCCATATTAAT |
| Ri-AbMPO1-R2 | Reverse primer for construction of RNAi interference plasmid of *AbMPO1* | GAGAGGACACGCTCGAGGCTCGCCTTGGAGGAAGTCTAG |
| Ri-AbMPO2-F1 | Forward primer for construction of RNAi interference plasmid of *AbMPO2* | GCAAGCTTCTGCTTTCTCCATTGAAACTC |
| Ri-AbMPO2-R1 | Reverse primer for construction of RNAi interference plasmid of *AbMPO2* | GCTCTAGAACCAAAACAGCCTCGACA |
| Ri-AbMPO2-F2 | Forward primer for construction of RNAi interference plasmid of *AbMPO2* | GCGGTACCCTGCTTTCTCCATTGAAACTC |
| Ri-AbMPO2-R2 | Reverse primer for construction of RNAi interference plasmid of *AbMPO2* | GCCTCGAGACCAAAACAGCCTCGACA |

**Supplementary Table 27. Primers used for *PPAR*s cloning and constructing protein expression plasmids.**

| Primer name | Purpose | Sequence (5' to 3') |
|---|---|---|
| AbPPAR-F | Forward primer for cloning *AbPPAR* | ATGGCTGATTGCAGGAGCTATTCT |
| AbPPAR-R | Reverse primer for cloning *AbPPAR* | TTACCAAGAAGGGAGTGTAGAATTTGGC |
| AbPPAR-28-F | Forward primer for constructing protein expression plasmid of *AbPPAR* | AATGGGTCGCGGATCCATGGCTGATTGCAGGAGCT |
| AbPPAR-28-R | Reverse primer for constructing protein expression plasmid of *AbPPAR* | GGTGGTGGTGCTCGAGCCAAGAAGGGAGTGTAGAATTTGGCA |
| DsPPAR-F | Forward primer for cloning *DsPPAR* | ATGGGTGATTGCAGGAGCTATT |
| DsPPAR-R | Reverse primer for cloning *DsPPAR* | GTCCAAATACTCCATAAAGGGTTTAGTGGT |
| DsPPAR-28-F | Forward primer for constructing protein expression plasmid of *DsPPAR* | AATGGGTCGCGGATCCATGGGTGATTGCAGGAGCT |
| DsPPAR-28-R | Reverse primer for constructing protein expression plasmid of *DsPPAR* | GGTGGTGGTGCTCGAGGTCCAAATACTCCATAAAGGGTT |
| CaPPAR-F | Forward primer for cloning *CaPPAR* | ATGGTAAATCAATCTTCCAAGGCAACTAGT |
| CaPPAR-R | Reverse primer for cloning *CaPPAR* | ATCATATTGCTTGATAGCAGTAATCAAGGGT |
| CaPPAR-28-F | Forward primer for constructing protein expression plasmid of *CaPPAR* | AATGGGTCGCGGATCCATGGTAAATCAATCTTCCAA |
| CaPPAR-28-R | Reverse primer for constructing protein expression plasmid of *CaPPAR* | GGTGGTGGTGCTCGAGATCATATTGCTTGATAGCAG |
| PaPPAR-F | Forward primer for cloning *PaPPAR* | ATGGGGGAACTATTTGATGAACAGC |
| PaPPAR-R | Reverse primer for cloning *PaPPAR* | GTCCAGGTGGGTGACTGGT |
| PaPPAR-28-F | Forward primer for constructing protein expression plasmid of *PaPPAR* | AATGGGTCGCGGATCCATGGGGGAACTATTTGATGAACAG |
| PaPPAR-28-R | Reverse primer for constructing protein expression plasmid of *PaPPAR* | GGTGGTGGTGGTGGTGCTCGAGGTCCAGGTGGGTGACTGGTGA |
| SlPPAR-F | Forward primer for cloning *SlPPAR* | ATGAGCGAAGCTAAAAGCGAATTG |
| SlPPAR-R | Reverse primer for cloning *SlPPAR* | GTCATCCAAAACTGGAGAAAGCACAG |
| SlPPAR-28-F | Forward primer for constructing protein expression plasmid of SlPPAR | AATGGGTCGCGGATCCATGAGCGAAGCTAAAAGCGAATTG |
| SlPPAR-28-R | Reverse primer for constructing protein expression plasmid of *SlPPAR* | GGTGGTGGTGCTCGAGGTCATCCAAAACTGGAGAAAGCACAG |

**Supplementary Table 28. Primers used for *LS*s cloning and constructing overexpression plasmids.**

| Primer name | Purpose | Sequence (5' to 3') |
|---|---|---|
| DsLS-F | Forward primer for cloning *DsLS* | ATGAGGAAAAAGAAAATGAGGAATTCAATGTTGAAC |
| DsLS-R | Reverse primer for cloning *DsLS* | CTATAGAGATTCATTAGATAACCATCTCTTGAGCATGGCC |
| DsLS-inpEAQ-F | Forward primer for constructing overexpression plasmid of *DsLS* | CTGCCCAAATTCGCGACCGGTATGAGGAAAAAGAAAATGAGGAATTC |
| DsLS-inpEAQ-R | Reverse primer for constructing overexpression plasmid of *DsLS* | ACCAGAGTTAAAGGCCTCGAGCTATAGAGATTCATTAGATAACCAT |
| AbLS-inpEAQ-F | Forward primer for constructing overexpression plasmid of *AbLS* | CTGCCCAAATTCGCGACCGGTATGAAGAAAACAATTGTGGTTCC |
| AbLS-inpEAQ-R | Reverse primer for constructing overexpression plasmid of *AbLS* | ACCAGAGTTAAAGGCCTCGAGTTATAGAGGTTGATAATATATCCA |
| AbUGT1-inpEAQ-F | Forward primer for constructing overexpression plasmid of *AbUGT1* | ATGGAAGAATCAAAAGTGTCCATGATGAATTG |
| AbUGT1-inpEAQ-R | Reverse primer for constructing overexpression plasmid of *AbUGT1* | CAAATTCGCGACCGGTATGGGCTTAGGACATGTCAATCCA |
| LcLS-F | Forward primer for cloning *DsLS* | ATGGCGAAAACAGAACTATGTTATTTGCT |
| LcLS-R | Reverse primer for cloning *DsLS* | TTACAAAGGCTTGTAAGACATCCACCT |
| LcLS-inpEAQ-F | Forward primer for constructing overexpression plasmid of *LcLS* | CAAATTCGCGACCGGTATGGCGAAAACAGAACTATGTTATTTGCT |
| LcLS-inpEAQ-R | Reverse primer for constructing overexpression plasmid of *LcLS* | AGTTAAAGGCCTCGAGTTACAAAGGCTTGTAAGACATCCACCTT |

**Supplementary Table 29. Primers used for *TRI*s cloning and constructing protein expression plasmids.**

| Primer name | Purpose | Sequence (5' to 3') |
|---|---|---|
| AbTRI-F | Forward primer for cloning *AbTRI* | ATGGAAGAATCAAAAGATAACATGAATGGCAAC |
| AbTRI-R | Reverse primer for cloning *AbTRI* | AAACCCACCATTAGCTGTGAATCC |
| AbTRI-28-F | Forward primer for constructing protein expression plasmid of AbTRI | AATGGGTCGCGGATCCATGGAAGAATCAAAAGATAACATGAATGGCAAC |
| AbTRI-28-R | Reverse primer for constructing protein expression plasmid of AbTRI | GGTGGTGGTGCTCGAGAAACCCACCATTAGCTGTGAATCCC |
| DsTRI-F | Forward primer for cloning *DsTRI* | ATGGAAGAATCAAAAGTGTCCATGATGAATTG |
| DsTRI-R | Reverse primer for cloning *DsTRI* | AAACCCACCATTAGCTGTGAATCC |
| DsTRI-28-F | Forward primer for constructing protein expression plasmid of DsTRI | AATGGGTCGCGGATCCATGGAAGAATCAAAAGTGTCCATG |
| DsTRI-28-R | Reverse primer for constructing protein expression plasmid of DsTRI | GGATTCACAGCTAATGGTGGGTTTCTCGAGCACCACCACC |
| CaTRI-F | Forward primer for cloning *CaTRI* | ATGGAAGAATCAAAAGTTTGCATGAATGT |
| CaTRI-R | Reverse primer for cloning *CaTRI* | AAATCCACCATTAGCAGTAAATCCACCATC |
| CaTRI-28-F | Forward primer for constructing protein expression plasmid of CaTRI | AATGGGTCGCGGATCCATGGAAGAATCAAAAGTTTGCATG |
| CaTRI-28-R | Reverse primer for constructing protein expression plasmid of CaTRI | GGTGGTGGTGCTCGAGAAATCCACCATTAGCAGTAAATCC |
| StTRI-F | Forward primer for cloning *StTRI* | ATGGCAGAATTGAGAGAAAAATGGAGT |
| StTRI-R | Reverse primer for cloning *StTRI* | AAACCCACCATTAGCTGTAAATCCACCAT |
| StTRI-28-F | Forward primer for constructing protein expression plasmid of StTRI | AATGGGTCGCGGATCCATGGCAGAATTGAGAGAAAAATGGAGTCT |
| StTRI-28-R | Reverse primer for constructing protein expression plasmid of StTRI | GGTGGTGGTGGTGCTCGAGAAACCCACCATTAGCTGTAAATCC |
| StTRI-L159V-F | Forward primer for constructing protein expression plasmid of StTRI-L159V | CATTACCTTCTGTATCTCTATACTCTGCTTCCAAAG |
| StTRI-L159V-R | Reverse primer for constructing protein expression plasmid of StTRI-L159V | CTGAAAATCCACCAATAGAAGAAACAAAAATAATATTTCCAT |
| SlTRI-F | Forward primer for cloning *SlTRI* | ATGGCAGAATTAAGAGAAAAATGGAGTCTTGA |
| SlTRI-R | Reverse primer for cloning *SlTRI* | AAACCCACCATTAGCTGTAAATCCAC |
| SlTRI-28-F | Forward primer for constructing protein expression plasmid of SlTRI | AATGGGTCGCGGATCCATGGCAGAATTAAGAGAAAAATGGA |
| SlTRI-28-R | Reverse primer for constructing protein expression plasmid of SlTRI | GGTGGTGGTGCTCGAGAAACCCACCATTAGCTGTAAATCCAC |
| SlTRI-L159V-F | Forward primer for constructing protein expression plasmid of SlTRI-L159V | CCCTCTGTATCTCTTTATTCCGC |
| StTRI-L159V-R | Reverse primer for constructing protein expression plasmid of SlTRI-L159V | TAACGCTGAAAATCCAGCAATAG |

**Supplementary Table 30. Primers used for *CYP82M3*s cloning and constructing overexpression plasmids.**

| Primer name | Purpose | Sequence (5' to 3') |
|---|---|---|
| CaCYP80M3-inpQ-F | Forward primer for constructing overexpression plasmid of *CaCYP82M3* | CCAAATTCGCGACCGGTATGTTTGATGATATTTCTTT |
| CaCYP80M3-inpQ-R | Reverse primer for constructing overexpression plasmid of *CaCYP82M3* | GAGTTAAAGGCCTCGAGTTAAAGTTCATAAAGCGTAG |
| SlyCYP82M3-inpQ-F | Forward primer for constructing overexpression plasmid of *SlCYP82M3* | CCAAATTCGCGACCGGTATGTTTGATAATTTTTATTT |
| SlyCYP82M3-inpQ-R | Reverse primer for constructing overexpression plasmid of *SlCYP82M3* | GAGTTAAAGGCCTCGAGCTAAAGTTCATAAAGCATAG |

**Supplementary Table 31. Primers used for *EVM0022661.2* cloning and constructing overexpression and CRIPSR plasmids.**

| Primer name | Purpose | Sequence (5' to 3') |
|---|---|---|
| BamHI-pBI121-EVM0022661.2 -F | Forward primer for constructing *EVM0022661.2* overexpression plasmid | TAGAGGATCCATGGATTTTCATATTCTTTCCCATG |
| SacI-pBI121-EVM0022661.2 -R | Reverse primer for constructing *EVM0022661.2* overexpression plasmid | TTCGAGCTCTTAAACCTCATAATACACAGGAATCAT |
| Cas9N-EVM0022661.2 -F | Forward primer for constructing *EVM0022661.2* CRISPR plasmid | GATTGAGAAGTGGAGAGTAGCATCA |
| Cas9N-EVM0022661.2 -R | Reverse primer for constructing *EVM0022661.2* CRISPR plasmid | AAACTGATGCTACTCTCCACTTCTC |
| qPCR-EVM0022661.2 -F | Forward primer for quantification of *EVM0022661.2* | GTGGAGAGTAGCATCAAGGA |
| qPCR-EVM0022661.2 -R | Reverse primer for quantification of *EVM0022661.2* | CACCAATCATCTTCACCGTTAT |
| EVM0022661.2 -knock-F | Forward primer for genotyping and sequencing | GTCCGCCAAGGAATGTTCACA |
| EVM0022661.2 -knock-R | Reverse primer for genotyping and sequencing | CGTAAGTTCATCCGAACCCAT |

**Supplementary Reference:**


1. Gu, Z., Eils, R. & Schlesner, M. Complex heatmaps reveal patterns and correlations in multidimensional genomic data. *Bioinformatics* **32**, 2847–2849 (2016).
2. Frazer, K. A., Pachter, L., Poliakov, A., Rubin, E. M. & Dubchak, I. VISTA: computational tools for comparative genomics. *Nucleic Acids Res.* **32**, 273–279 (2004).
3. Qin, C. *et al.* Whole-genome sequencing of cultivated and wild peppers provides insights into *Capsicum* domestication and specialization. *Proc. Natl Acad. Sci. USA* **111**, 5135–5140 (2014).
4. Xu, X. *et al.* Genome sequence and analysis of the tuber crop potato. *Nature* **475**, 189–195 (2011).
5. Kumar, V. *et al.* Identification of tomato root growth regulatory genes and transcription factors through comparative transcriptomic profiling of different tissues. *Physiol. Mol. Biol. Plants* **27**, 1173–1189 (2021).
6. Bodenhofer, U., Bonatesta, E., Horejš-Kainrath, C. & Hochreiter, S. Msa: an R package for multiple sequence alignment. *Bioinformatics* **31**, 3997–3999 (2015).
7. Zeng, L. *et al.* Development of *Atropa belladonna* L. plants with high-yield hyoscyamine and without its derivatives using the CRISPR/Cas9 system. *Int. J. Mol. Sci.* **22**, 1731 (2021).
8. Shen, W., Le, S., Li, Y. & Hu, F. SeqKit: a cross-platform and ultrafast toolkit for FASTA/Q file manipulation. *PLoS One* **11**, e0163962 (2016).
