# Supplementary material for: Revealing evolution of tropane alkaloid biosynthesis by analyzing two genomes in the Solanaceae family
Source: Nat Commun. 2023 Mar 15;14:1446. doi: 10.1038/s41467-023-37133-4 (PMC10017790; doi:10.1038/s41467-023-37133-4)
Supplement: Supplementary file 3 — Description of Additional Supplementary Files [file 41467_2023_37133_MOESM3_ESM.pdf]

Description of Additional Supplementary Files

File name: Supplementary Data 1

Description: Gene ontology (GO) enrichment analysis of expanded gene families in *Atropa belladonna*.

File name: Supplementary Data 2

Description: Gene ontology (GO) enrichment analysis of the expanded gene families in *Datura stramonium*.

File name: Supplementary Data 3

Description. The information on genes in the “black” module generated by WGCNA analysis.

File name: Supplementary Data 4

Description. The information on genes in the “blue” module generated by WGCNA analysis.

File name: Supplementary Data 5

Description. The information on genes in the “brown” module generated by WGCNA analysis.

File name: Supplementary Data 6

Description. The information on genes in the “green” module generated by WGCNA analysis.

File name: Supplementary Data 7

Description. The information on genes in the “red” module generated by WGCNA analysis.

File name: Supplementary Data 8

Description. The information on genes in the “turquoise” module generated by WGCNA analysis.

File name: Supplementary Data 9

Description. The information on genes in the “yellow” module generated by WGCNA analysis.
