# Supplementary material for: Revealing evolution of tropane alkaloid biosynthesis by analyzing two genomes in the Solanaceae family
Source: Nat Commun. 2023 Mar 15;14:1446. doi: 10.1038/s41467-023-37133-4 (PMC10017790; doi:10.1038/s41467-023-37133-4)
Supplement: Supplementary file 13 — Reporting Summary [file 41467_2023_37133_MOESM13_ESM.pdf]

nature portfolio

Corresponding author(s): Zhihua Liao

Last updated by author(s): Feb 27, 2023

Reporting Summary

Nature Portfolio wishes to improve the reproducibility of the work that we publish. This form provides structure for consistency and transparency in reporting. For further information on Nature Portfolio policies, see our Editorial Policies and the Editorial Policy Checklist.

Statistics

For all statistical analyses, confirm that the following items are present in the figure legend, table legend, main text, or Methods section.

n/a	Confirmed	
☐	☒	The exact sample size (*n*) for each experimental group/condition, given as a discrete number and unit of measurement
☐	☒	A statement on whether measurements were taken from distinct samples or whether the same sample was measured repeatedly
☐	☒	The statistical test(s) used AND whether they are one- or two-sided<br>*Only common tests should be described solely by name; describe more complex techniques in the Methods section.*
☒	☐	A description of all covariates tested
☐	☒	A description of any assumptions or corrections, such as tests of normality and adjustment for multiple comparisons
☐	☒	A full description of the statistical parameters including central tendency (e.g. means) or other basic estimates (e.g. regression coefficient) AND variation (e.g. standard deviation) or associated estimates of uncertainty (e.g. confidence intervals)
☐	☒	For null hypothesis testing, the test statistic (e.g. *F*, *t*, *r*) with confidence intervals, effect sizes, degrees of freedom and *P* value noted<br>*Give P values as exact values whenever suitable.*
☒	☐	For Bayesian analysis, information on the choice of priors and Markov chain Monte Carlo settings
☒	☐	For hierarchical and complex designs, identification of the appropriate level for tests and full reporting of outcomes
☒	☐	Estimates of effect sizes (e.g. Cohen's *d*, Pearson's *r*), indicating how they were calculated

Our web collection on statistics for biologists contains articles on many of the points above.

Software and code

Policy information about availability of computer code

Data collection	sequencing data were collected form Oxford Nanopore and Illumina sequencing machines. GRIDION X5 v.9.4.1; GUPPY v.3.2.2 - nanopore base calling; PacBio Sequel2 platform with circular consensus sequencing model was used for PacBio long reads sequencing; The short reads were filtered using fastp v0.19.4.
Data analysis	Jellyfish v2.2.10 and GenomeScope were used for estimating the genome size; ONT reads were corrected and trimmed using Nextdenovo (v 2.0-beta.1), and subsequently the corrected ONT reads were directly assembled using SMARTDENOVO (v 1.0.0). The assembled contigs were polished four times by PILON (v 1.18) and NextPolish (v 1.0.5). BUSCO (v 5.3.2), Assembly and annotation evaluation; BWA (v 0.7.10-r789) and SAMtools (v 1.9)- alignment and SAM/BAM format conversion. Iso-Seq2 (v5.1.0), full-length transcriptome assemble. Hi-C Pro (v 2.11.4), LACHESIS (v 1.0.0), SLR (v 1.0.0), SALSA (v 2.2), scaffold assemble; Tandem Repeats Finder (v 4.0.9), RepeatMasker (v.4.0.5), RepeatProteinMasker (v 1.36), RepeatModeler (v 1.0.9), LTR_FINDER (v 1.06), LTRharvest (v 1.5.10), LTR_retriever (v 3.0), RepeatMasker (v 4.0.5), repeat identification and annotation; MAKER (v 2.31.9), GeMoMa (v 1.3.1), GeneWise (v 2.4.1), homology-based predictions; Augustus (v3.2.1), GlimmerHMM (v3.0.4), SNAP (v 2006-07-28), de novo prediction; seqclean, PASA (v 2.1.0), TransDecoder (v 3.0.0), transcriptome-based predictions. EVidenceModeler (v1.1.1), annotation integration; INFERNAL (v.1.1.2), tRNAscan-SE (v 2.0), ncRNAs annotation; Blast2GO pipeline (v 3.1.3), GO annotation. BLASTP (v 2.2.29), OrthoMCL (v.2.0.9), MCL (v 14.137), single-copy homologous genes extraction; MAFFT (v7.407), Gblocks (v 0.91b), RAxML (v.8.1.13), genomic phylogenetic tree construction; PAML (v4.9e), divergence time estimation. CAFE (v 3.1), identify expansions and contractions of gene families; MCScanX (v 11-13-2012), WGD analysis; CIRCOS (v 0.69.6), visualization; StringTie (v 1.3.3b), HISAT2 (v 2.0.5), DESeq2 (v 1.27.12), sva (v 3.11), TRIMMOMATIC (v 0.39), WGCNA (v.1.69), RNA-seq analysis. HMMER (v3.3), domain identification. Cytoscape (v 3.5.1), display the network of WGCNA results. R (v 3.1), run R package. OriginPro (v 8.0), regression of the Michaelis–Menten equation.

For manuscripts utilizing custom algorithms or software that are central to the research but not yet described in published literature, software must be made available to editors and reviewers. We strongly encourage code deposition in a community repository (e.g. GitHub). See the Nature Portfolio guidelines for submitting code & software for further information.

Data

Policy information about availability of data

All manuscripts must include a data availability statement. This statement should provide the following information, where applicable:

- Accession codes, unique identifiers, or web links for publicly available datasets
- A description of any restrictions on data availability
- For clinical datasets or third party data, please ensure that the statement adheres to our policy

The genomic sequencing and transcriptome data generated in this study have been deposited in the NCBI Sequence Read Archive (SRA) database under accession code PRJNA766188 [https://www.ncbi.nlm.nih.gov/bioproject/PRJNA766188/] for Atropa belladonna and PRJNA765895 for Datura stramonium [https://www.ncbi.nlm.nih.gov/bioproject/PRJNA765895/]. The full-length transcriptome data of Lycium chinense have been deposited in the NCBI Sequence Read Archive (SRA) database under accession code PRJNA769498 [https://www.ncbi.nlm.nih.gov/bioproject/PRJNA769498/]. The genome assembly and annotation of A. belladonna and D. stramonium were also deposited in Genome Warehouse at the National Genomics Data Center (https://ngdc.cncb.ac.cn/) under accession number GWHBOWM00000000 [https://ngdc.cncb.ac.cn/bioproject/browse/PRJCA012583] and GWHBOZL00000000 [https://ngdc.cncb.ac.cn/bioproject/browse/PRJCA012583], respectively. The source data underlying Figs. 3b, 3d, 4e, 4f, 5c, 6b, 6d, 6g, and 6h, Supplementary Figures 11-23, 39, 46, 47, and 49 are provided as a Source Data file.
Those database are used in this study:
Solanaceae Genomics Network, https://solgenomics.net/;
Genome database of eggplant, http://www.eggplant-hq.cn/;
Genome database of coffee, http://coffee-genome.org/;
phytozome database, www.phytozome.net;
plant genome database, http://www.plantgdb.org/VvGDB/.

Human research participants

Policy information about studies involving human research participants and Sex and Gender in Research.

Reporting on sex and gender	N/A
Population characteristics	N/A
Recruitment	N/A
Ethics oversight	N/A

Note that full information on the approval of the study protocol must also be provided in the manuscript.

Field-specific reporting

Please select the one below that is the best fit for your research. If you are not sure, read the appropriate sections before making your selection.

☒ Life sciences ☐ Behavioural & social sciences ☐ Ecological, evolutionary & environmental sciences

For a reference copy of the document with all sections, see nature.com/documents/nr-reporting-summary-flat.pdf

Life sciences study design

All studies must disclose on these points even when the disclosure is negative.

Sample size	One individual plant is sufficient for the genome sequencing for each species. We have described the sample size and the statistical method in each individual experiment.
Data exclusions	no data exclusions
Replication	To avoid affecting genome assembly, we selected one Atropa belladonna plant and one Datura stramonium plant for genome sequencing, and therefore no replication was performed. All replication of other experiments, such as metabolites analysis, qRT-PCR, the phylogenetic tree constructing, enzymatic kinetic analysis, were successful, and we have described the number of replication in each individual experiment.
Randomization	There were no filed or lab experiment that might require randomization.
Blinding	This is a study on plants, therefore the question is not of relevance.

Reporting for specific materials, systems and methods

We require information from authors about some types of materials, experimental systems and methods used in many studies. Here, indicate whether each material, system or method listed is relevant to your study. If you are not sure if a list item applies to your research, read the appropriate section before selecting a response.

Materials & experimental systems

n/a	Involved in the study
☒	☐ Antibodies
☒	☐ Eukaryotic cell lines
☒	☐ Palaeontology and archaeology
☒	☐ Animals and other organisms
☒	☐ Clinical data
☒	☐ Dual use research of concern

Methods

n/a	Involved in the study
☒	☐ ChIP-seq
☒	☐ Flow cytometry
☒	☐ MRI-based neuroimaging
